# Supplementary material for: Uncovering the Worldwide Diversity and Evolution of the Virome of the Mosquitoes Aedes aegypti and Aedes albopictus
Source: Microorganisms. 2021 Aug 3;9(8):1653. doi: 10.3390/microorganisms9081653 (PMC8398489; doi:10.3390/microorganisms9081653)
Supplement: Supplementary file 1 [file microorganisms-09-01653-s001.zip › Supplementary Files_proof.pdf]

Supplementary File 1: Annotations and mapping coverage of novel and updated *Aedes* viruses

Formosus virus

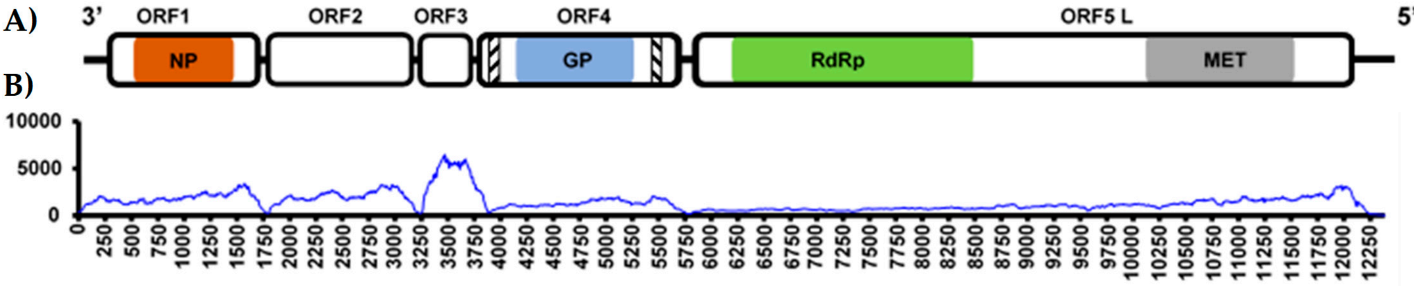

Figure S1. Genome organization and mapping coverage of Formosus virus. **A**) Coloured boxes correspond to predicted protein domains, and boxes with dashed lines represent predicted TM domains. NP, nucleoprotein; GP, glycoprotein; RdRp, RNA-dependent RNA polymerase; MET, methyltransferase. **B**) Sub-genomic transcription profile shown through mapped RNA-Seq data from *Ae. aegypti* samples originating from Bundibugyo, Uganda (BioProject Accession: PRJNA233429) (1).

Table S1. Genome features and annotation of Formosus virus

Open Reading frame	Genomic location	Size of protein (aa)	Gene name	Closest virus protein by BLASTp; Accession number	Coverage (%); Identity (%)	E-value	Predicted protein domain; location	Score; E-value	Notes
ORF1	35- 1657	540	NP	nucleoprotein [New Kent County virus]; AUW34396	85%; 26.12%	6.00e-38	**pfam00945** Rhabdovirus nucleocapsid protein; 72..417	190; 4e-54	
ORF2	1794 - 3110	438	ORF2	-	-	-	-	-	
ORF3	3177 - 3686	169	ORF3	-	-	-	-	-	
ORF4	3714 - 5516	600	GP	glycoprotein [Merida-like virus Turkey] ; ATW01053	62%; 29%	2.00e-42	**pfam00974** Rhabdovirus spike glycoprotein.	141; 5e-36	TM domains: 41-63 508-530
ORF5	5650 - 12051	2133	L / RdRP	large polymerase protein [Isfahan virus]; YP_007641386	99%; 39%	0	**pfam00946** Mononegavirales RNA dependent RNA polymerase; 52..1066<br><br>**pfam14314** Virus-capping methyltransferase; 1363..2023<br><br>**pfam14318** Mononegavirales mRNA-capping region V; 1092..1323	**pfam00946** 701; 0.0<br><br>**pfam14314** 375; 7e-112<br><br>**pfam14318** 156, 2e-41	

San Gabriel virus

A)

B)

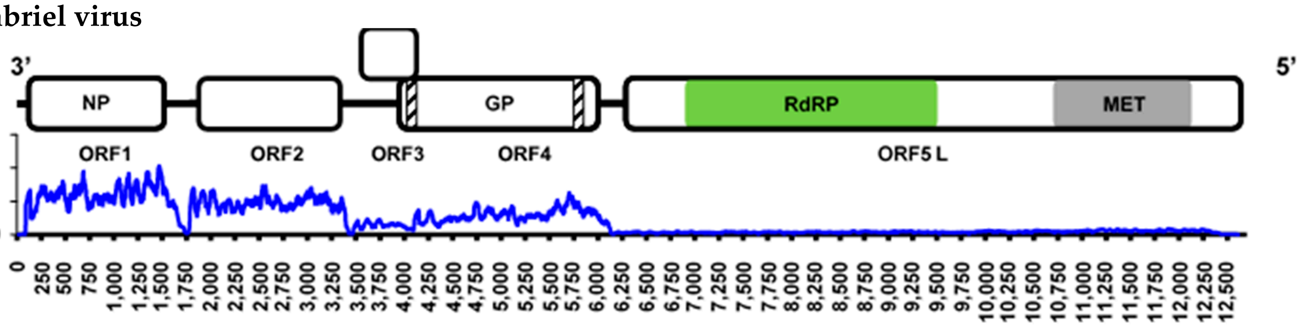

Figure S2. Genome organization and mapping coverage of San Gabriel virus. A) Coloured boxes correspond to predicted protein domains, and boxes with dashed lines represent predicted TM domains NP, nucleoprotein; GP, glycoprotein; RdRp, RNA-dependent RNA polymerase; MET, methyltransferase. **B**) Sub-genomic transcription profile shown through mapped RNA-Seq data from *Ae. albopictus* samples originating from San Gabriel, USA (BioProject Accession: PRJNA563095) (4).

Table S2. Genome features and annotation of San Gabriel virus.

Open Reading frame	Genomic location	Size of protein (aa)	Gene name	Closest virus protein by BLASTp; Accession number	Coverage (%); Identity (%)	E-value	Predicted protein domain(s); location	Score; E-value	Notes
ORF1	129-1619	496	NP	hypothetical protein 1 [Hubei rhabdo-like virus 1]; YP_009336980	78% ; 27.27%	2e-37	**PF03216** Rhabdovirus nucleoprotein; 181..383	**PF03216;** 1e-13	-
ORF2	1851 - 3311	486	ORF2	-	-	-	-	-	-
ORF3	3841 - 3927	148	ORF3	-	-	-	-	-	-
ORF4	3878 - 5941	687	GP	envelope glycoprotein [Lasius neglectus virus 2]; AYW51542	47%, 22.75%	6e-14	-		TM domain: 556-578
ORF5	6194 - 12438	2081	L / RdRP	RNA-dependent RNA polymerase [Wuhan Ant Virus]; YP_009304559	97%, 39.34%	0	**pfam00946** Mononegavirales RNA dependent RNA polymerase; 194..1041 **pfam14314** Virus-capping methyltransferase; 1062..1299 **pfam14318** Mononegavirales mRNA-capping region V; 1660..1840	**pfam00946;** 411; 1.4e-138 **pfam14314** 146; 5.6e-39 **pfam14318** 131; 9.5e-10	-

Longgang mosquito virus

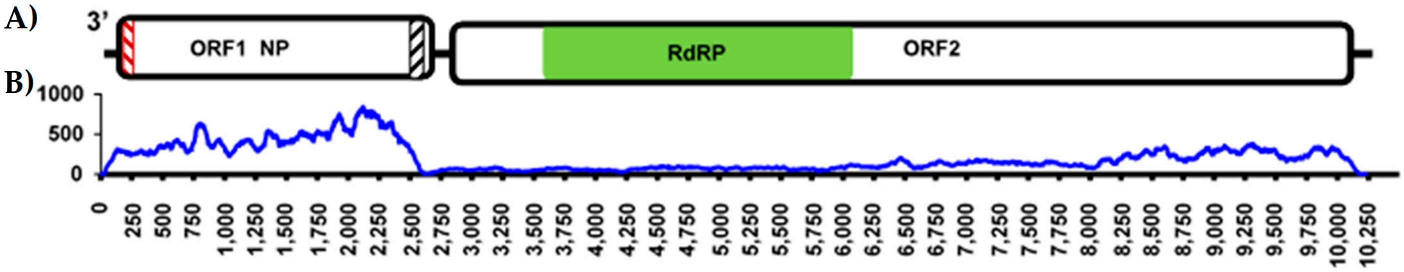

Figure S3. Genome organization and mapping coverage of Longgang mosquito virus. A) Coloured boxes correspond to predicted protein domains, black boxes with dashed lines represent predicted TM domains and red boxes with dashed lines indicate predicted signal peptides. NP, nucleoprotein; RdRp, RNA-dependent RNA polymerase. **B)** Sub-genomic transcription profile shown through mapped RNA-Seq data from *Ae. albopictus* mosquitoes originating from Longgang District, Shenzhen, China (BioProject: PRJNA475859) (5)

Table S3. Genome features and annotation of Longgang mosquito virus

Open Reading frame	Genomic location	Size of protein (aa)	Gene name	Closest virus protein by BLASTp; Accession number	Coverage (%); Identity (%)	E-value	Predicted protein domain(s); location	Score; E-value	Notes
ORF1	71-2605	844	NP	putative nucleoprotein [Scaldis River bee virus]; APT68158	32%, 23.24%	8e-07	**pfam05505** Ebola_NP, Ebola nucleoprotein; 537..718	**pfam05505** 37.1; 0.058	Signal peptide cleavage site: position: 38: (LG-DV) Score: 0.477 TM domain: 824-843
ORF2	2640 - 9980	2446	L / RdRP	polymerase [Shayang Fly Virus 1]; YP_009300663	59%; 35.55%	0	**pfam00946**: Mononegavirales RNA dependent RNA polymerase; 303..1084 **pfam14318**: Mononegavirales mRNA-capping region V; 1106..1275	**pfam00946** 207; 5e-54 **pfam14318** 38.5; 0.033	-

Rabai virus

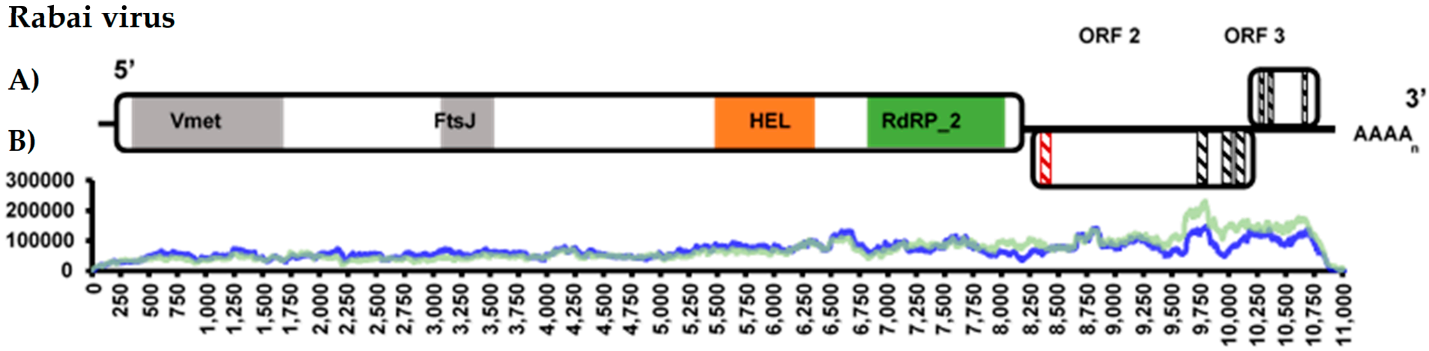

Figure S4. Genome organization and mapping coverage of Rabai virus. A) Coloured boxes correspond to predicted protein domains, black boxes with dashed lines represent predicted TM domains and red boxes with dashed lines indicate predicted signal peptides. Vmet, viral methyltransferase; FtsJ, methyltransferase; HEL, helicase; RdRp_2, RNA-

dependent RNA polymerase. **B)** Mapping coverage of RNA-Seq from *Ae. aegypti* samples from colonies established from Rabai, Kenya (K2; Green, K10, Blue) (BioProject Accession: PRJNA233429) (1).

Table S4. Genome features and annotation of Rabai virus

Open Reading frame	Genomic location	Size of protein (aa)	Gene name	Closest virus protein by BLASTp; Accession number	Coverage (%); Identity (%)	E-value	Predicted protein domain(s); location	Score; E-value	Notes
ORF1	94 - 8328	2744	RdRp	RNA-dependent RNA polymerase [Yongsan negev-like virus 1]; AXV43886	98%; 41.26%	0.0	**pfam01660** Viral methyltransferase; 60..320 **pfam01728** FtsJ-like methyltransferase; 982..1130 **pfam01443** Viral_helicase1; 1814..2101 **pfam00978** RNA dependent RNA polymerase 2; 2262..2676	**pfam01660** 88.5; 4e-18 **pfam01728** 44.5; 2e-04 **pfam01443** 78.2; 4e-15 **pfam00978** 214, 4e-59	-
ORF2	8277 - 10199	640	ORF2	hypothetical protein [Culex negev-like virus 1]; ASA47387	50%; 26.22%	4e-30	**-**	-	Signal peptide cleavage site: position 16-17 (IDA-QY) Score : 0.923 TM domains: 490-512 575-597 607-629
ORF3	10150 - 10818	222	ORF3	hypothetical protein [Culex negev-like virus 1]; YP_009388587	96%; 52.07%	2e-75	**-**	-	TM domains: 47-66 92-111 139-161 174-196

Aedes binegev-like virus 1 (AeBNV-1)

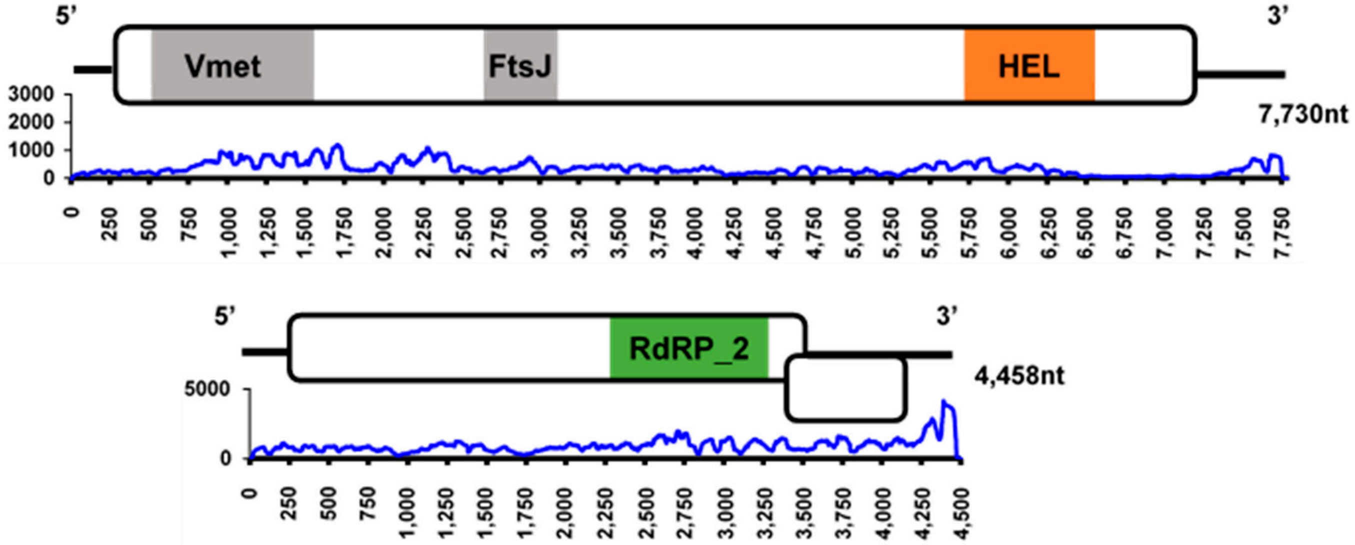

Figure S5. Genome organization and mapping coverage of Aedes binegev-like virus 1. Coloured boxes correspond to predicted protein domains. Vmet, viral methyltransferase; FtsJ, methyltransferase; HEL, helicase; RdRp_2, RNA-dependent RNA polymerase. Sequencing data taken from the Miami, USA *Ae. aegypti* colony originating from pooled soma and ovary libraries (BioProject Accession: PRJNA386859) (5).

Table S5. Genome features and annotation of Aedes binegev-like virus 1

Open Reading frame	Genomic location	Size of protein (aa)	Gene name	Closest virus protein by BLASTp; Accession number	Coverage (%); Identity (%)	E-value	Predicted protein domain(s); location	Score; E-value	Notes
**Segment 1 7,730nt**									
ORF1	68-6775	2236	ORF1	RdRp [Hubei virga-like virus 2]; YP_009337412	30%; 35.73%	1e-50	**pfam01660** Viral methyltransferase; 323..509 **pfam01728** FtsJ-like methyltransferase; 1031..1178 **pfam01443** Viral_helicase1 RNA helicase; 1796..2051	**pfam01660** 40.3; 0.010 **pfam01728** 50.3; 2e-06 **pfam01443:** 102; 3e-23	tblastn top hit: SsRNA virus-like 6 genomic sequence KX148585 Query length: 69% Identity: 39.85% E-value: 1e-132
**Segment 2 4,458nt**									
ORF1	107-3739	1211	RdRp	putative polyprotein [Blackford virus]; AMO03220	42.96%; 44%	1e-126	**pfam00978** RNA dependent RNA polymerase 2; 746..1191	**pfam00978** 277; 4e-82	tblastn top hit: SsRNA virus-like 5 genomic sequence KX148584 Query length: 51.55% Identity: 39% E-value: 4e-155
ORF2	3663 - 4193	177	ORF2	hypothetical protein [Hubei virga-like virus 12]; YP_009337823	100%; 43.26%	2e-42	-	-	-

Aedes binegev-like virus 2 (AeBNV-2)

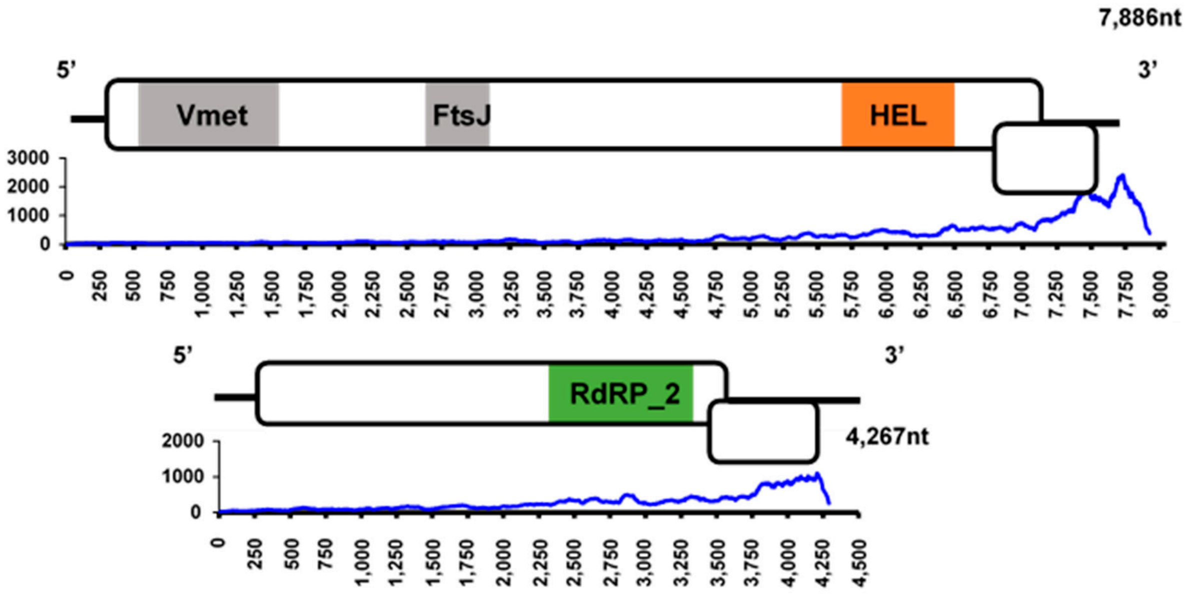

Figure S6. Genome organization and mapping coverage of Aedes binegev-like virus 2. Coloured boxes correspond to predicted protein domains. Vmet, viral methyltransferase; FtsJ, methyltransferase; HEL, helicase; RdRp_2, RNA-dependent RNA polymerase. Mapping coverage of virus segments from RNA-Seq of *Ae. albopictus* samples originating from Longgang district, Shenzhen, China (BioProject: PRJNA475859) (6).

Table S6. Genome features and annotation of Aedes binegev-like virus 2

Open Reading frame	Genomic location	Size of protein (aa)	Gene name	Closest virus protein by BLASTp; <u>Accession number</u>	Coverage (%); Identity (%)	E-value	Predicted protein domain(s); location	Score; E-value	Notes
**Segment 1 7,866nt**									
ORF1	96-7112	2338	ORF1	RdRp [Hubei virga-like virus 2]; <u>YP_009337412</u>	29%; 34.49%	1e-51	**pfam01660** Viral methyltransferase; 340..643 **pfam01728** FtsJ-like methyltransferase; 1048..1196 **pfam01443** Viral_helicase1; 1830..2085	**pfam01660** 45.4; 3e-04 **pfam01728** 48.7; 5e-06 **pfam01443** 95.5; 9e-21	<u>tblastn top hit:</u> SsRNA virus-like 6 genomic sequence <u>KX148585</u> Query length: 80% Identity: 40.70% E-value: 2e-137
ORF2	7623-6829	264	ORF2	-	-	-	-	-	-
**Segment 2 4,267nt**									
ORF1	99-3725	1208	RdRp	putative polyprotein [Blackford virus]; <u>AMO03220</u>	47%; 42.91%	4e-132	**pfam00978** RNA dependent RNA polymerase; 742..1188	**pfam00978** 268; 6e-79	<u>tblastn top hit:</u> SsRNA virus-like 5 genomic sequence <u>KX148584</u> Query length: 39% Identity: 52.08% E-value: 1e-154
ORF2	3646 - 4179	177	ORF2	hypothetical protein [Hubei virga-like virus 12]; <u>YP_009337823</u>	94%; 43.79%	2e-45	-	-	-

Aedes orthomyxo-like virus 2 (AOLV-2)

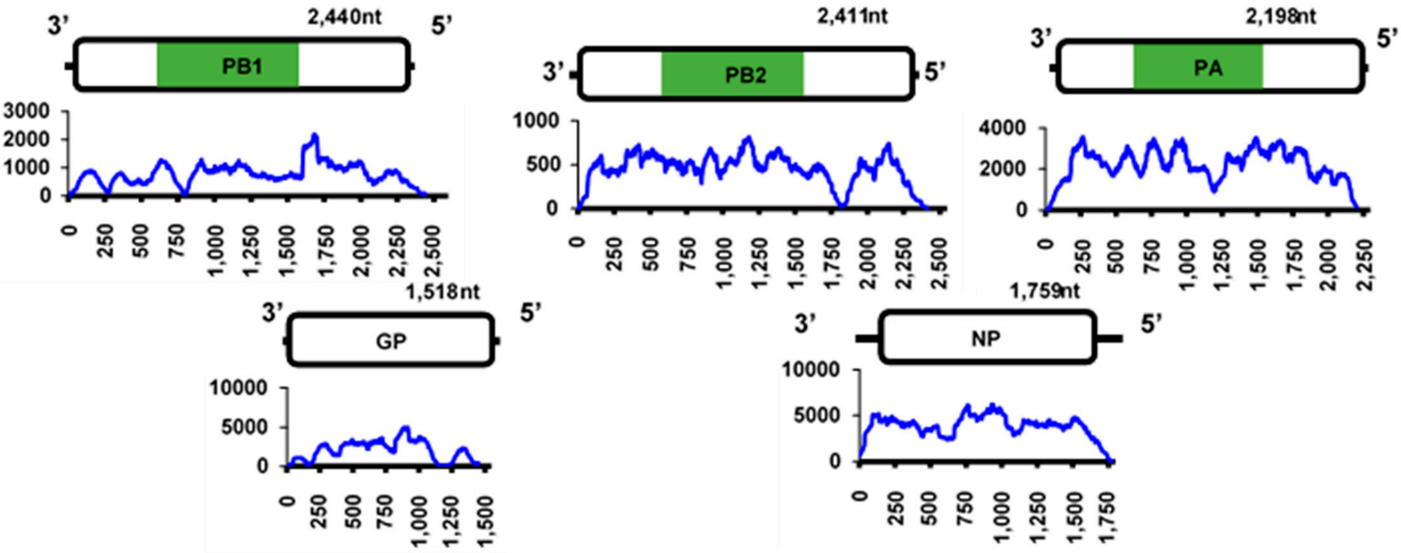

Figure S7. Genome organization and mapping coverage of Aedes orthomyxo-like virus 2 Coloured boxes correspond to predicted protein domains. PB1, RNA-dependent RNA polymerase basic subunit 1; PB2, RNA-dependent RNA polymerase basic subunit 2; PA, RNA-dependent RNA polymerase acidic subunit; NP, Nucleoprotein. Mapping

coverage of virus segments from *Ae. albopictus* RNA-Seq samples from Australia: Torres Strait Islands (BioProject accession: PRJNA450281) (7, 8).

Table S7. Genome features and annotation of Aedes orthomyxo-like virus 2

Open Reading frame	Genomic location	Size of protein (aa)	Gene name	Closest virus protein by BLASTp; Accession number	Coverage (%); Identity (%)	E-value	Predicted protein domain(s); location	Score; E-value	Notes
**Segment 1 PB1 2450nt**									
ORF1	36 - 2396	787	PB1 protein	PB1 [Whidbey virus]; AQU42764	96%; 76.25%	0.00	**pfam00602** Influenza RNA-dependent RNA polymerase subunit PB1; 33..734	185; 3e-49	-
**Segment 2 PB2 2411nt**									
ORF1	20 - 2392	791	PB2 protein	PB2 [Whidbey virus]; AQU42765	94%; 64.26%	0.00	-	-	-
**Segment 3 PA 2198nt**									
ORF1	31 - 2160	709	PA protein	PA [Whidbey virus]; AQU42766	100%; 65.22%	0.00	**pfam00603** Influenza RNA-dependent RNA polymerase subunit PA; 346..706	34.5; 0.22	Predicted monopartite (NLS): Position: 73 Motif: PPPLKRSRVE Score:10 Predicted bipartite NLS: Location: 536 Motif: DKIPIITVEMLNQDDGV KYSSFIKKAHV Score: 5
**Segment 4 NP 1759nt**									
ORF1	191 - 1642	484	NP protein	NP [Whidbey virus]; AQU42767	87%; 76%	0.0	-	-	-
**Segment 5 GP 1518nt**									
ORF1	37 - 1401	454	GP protein	-	-	-	-	-	Predicted bipartite NLS: Location: 37 Motif: EAKKPVALKTLHSVTSM ESKLVETVKATKVI Score: 5.9

Aedes orbi-like virus 1 (AOLV)

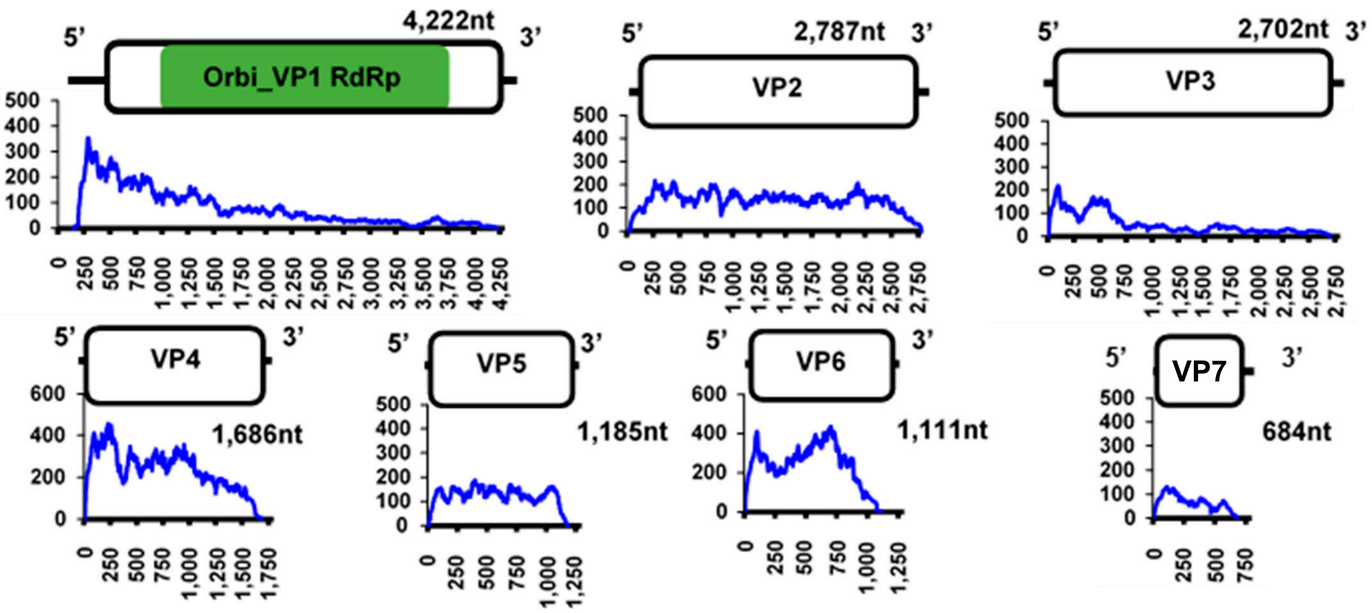

Figure S8. Genome organization and mapping coverage of Aedes orbi-like virus 1. Coloured boxes correspond to predicted protein domains. Orbvirus VP1 RdRp RNA-dependent RNA polymerase. Mapping coverage of each segment from RNA-Seq data from *Ae. aegypti* samples originating from Cairns, Australia (Sample B_8DPI_18_GAATTCGT_L003_R1_001) (10).

Table S8. Genome features and annotation of Aedes orbivirus 1

Open Reading frame	Genomic location	Size of protein (aa)	Gene name	Closest virus protein by BLASTp; Accession number	Coverage (%); Identity (%)	E-value	Predicted protein domain(s); location	Score; E-value
**VP1 4,222nt**								
ORF1	327 - 4169	1281	VP1	truncated putative RNA-dependent RNA polymerase-like protein [uncultured virus]; AGW51764	59%; 82.96%	0.0	**pfam05788** Orbivirus RNA-dependent RNA polymerase (VP1); 304..922	140; 3e-33
**VP2 2,781nt**								
ORF1	37-2703	889	VP2	hypothetical protein 1 [Hubei odonate virus 15]; APG79145	84%; 30.01%	6e-72	-	-
**VP3 2,702nt**								
ORF1	43 – 2676	878	VP3	hypothetical protein 2 [Hubei odonate virus 15]; APG79146	99%; 22.68%	4e-51	-	-
**VP4 1,686nt**								
ORF1	61 – 1581	507	VP4	-	-	-	-	-
**VP5 1,185nt**								
ORF1	28 – 1122	365	VP5	hypothetical protein 4 [Hubei odonate virus 15]; APG79148	92%; 30.32%	5e-41	-	-
**VP6 1,111nt**								
ORF1	7 - 1005	333	VP6	-	-	-	-	-
**VP7 684nt**								
ORF1	16 - 652	213	VP7	-	-	-	-	-

Aedes partiti-like virus 1 (APLV-1)

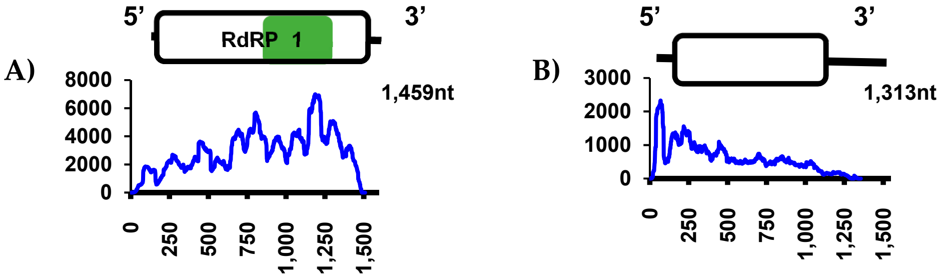

Figure S9. Genome organization and mapping coverage of Aedes partiti-like virus 1. Coloured green box corresponds to predicted protein domain RdRP 1, RNA-dependent RNA polymerase Mapping coverage of the genome. RNA-Seq data from whole mosquito Galveston, USA *Ae. aegypti* colony (BioProject Accession: PRJNA399504) (11).

Table S9. Genome features and annotation of Aedes partiti-like virus 1

Open Reading frame	Genomic location	Size of protein (aa)	Gene name	Closest virus protein by BLASTp; Accession number	Coverage (%); Identity (%)	E-value	Predicted protein domain(s); location	Score; E-value
**Segment 1: 1,459**								
ORF1	39 - 1453	472	RdRP	RdRp [Hubei partiti-like virus 34]; APG78322	99%; 52.77%	6e-174	**pfam00680** RNA dependent RNA polymerase 1; 84..376	98.2; 7e-22
**Segment 2: 1,313**								
ORF1	59-1126	355	Capsid	putative capsid protein [Atrato Partiti-like virus 3]; QHA33900	99%; 39.66%	3e-72		

Tiger mosquito bi-segmented tombus-like virus (TMBTV)

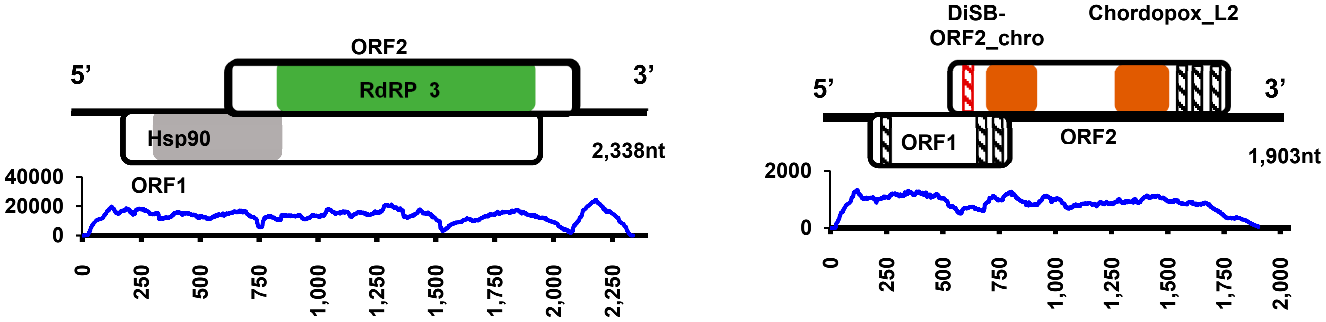

Figure S10. Genome organization and mapping coverage of Tiger mosquito bi-segmented tombus-like virus. Coloured boxes correspond to predicted protein domains, and predicted TM domains and signal peptides are depicted as black and red boxes with dashed lines. RdRP_3, RNA-dependent RNA polymerase 3; Hsp90, viral heat shock homologue. Mapping coverage of sub-genomic gene transcription profile. RNA-Seq data originating from whole *Ae. albopictus* Manassas, USA samples (BioProject accession: PRJNA158021) (3).

Table S10. Genome features and annotation of Tiger mosquito bi-segmented tombus-like virus

Open Reading frame	Genomic location	Size of protein (aa)	Gene name	Closest virus protein by BLASTp; Accession number	Coverage (%); Identity (%)	E-value	Predicted protein domain(s); location	Score; E-value	Notes
**Segment 1 2,338nt**									
ORF1	45-1886	614	Hsp90	hypothetical protein 2 [Hypsignathus monstrosus tombus-like virus 1]; AZS32326	56%; 54.05%	3e-98	**PF03225** Viral heat shock protein Hsp90 homologue 93..144	Score: 0.14	
ORF2	884-1999	373	RdRp	hypothetical protein 1 [Hypsignathus monstrosus tombus-like virus 1]; AZS32325	99%; 76.08%	0.0	**pfam00998** Viral RNA dependent RNA polymerase 3; 6..241	116; 1e-28	
**Segment 2 1,903nt**									
ORF1	83-547	155		hypothetical protein [Culex mosquito virus 1]; AXQ04816	83%; 33.08%	3e-14	-	-	TM domains: 20-39 82-104 124-146
ORF2	525-1649	375	Coat	hypothetical protein [Culex mosquito virus 1]; AXQ04817	83%; 26.56%	1e-28	**PF16506** Putative virion glycoprotein of insect viruses; 89..109 **PF05803** Chordopoxvirus L2 protein; 306..366	**PF16506** Score: 0.059 **PF05803:** Score: 0.0091	Signal peptide cleavage site: Position: 23/24 (AAA-QL) Score: 0.927 TM domains: 292-314 326-348 352-374

Updated viruses

Liverpool tombus-like virus (LTLV), formerly Mosquito nodavirus

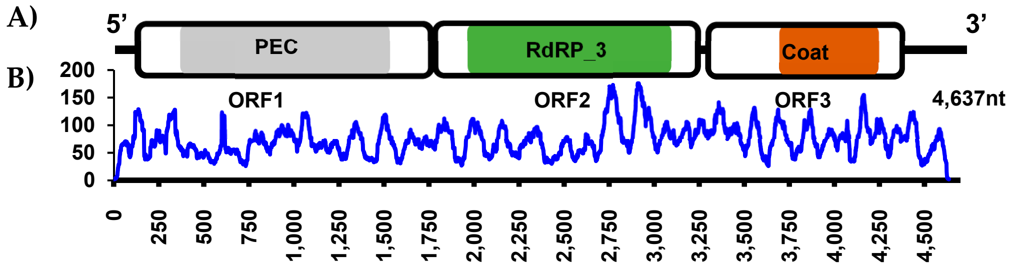

Figure S11. Genome organization and mapping coverage of Liverpool tombus-like virus. A) Coloured boxes correspond to predicted protein domains. PEC, Probable pectinesterase; RdRP_3, RNA-dependent RNA polymerase 3; Capsid, Nodavirus capsid protein. B) Mapping coverage of Liverpool virus from RNA-Seq data originating from *Ae. aegypti* samples from the Liverpool strain (BioProject: PRJNA232599) (14)

Table S11. Genome features and annotation of Liverpool tombus-like virus

Open Reading frame	Genomic location	Size of protein (aa)	Gene name	Closest virus protein by BLASTp; Accession number	Coverage (%); Identity (%)	E-value	Predicted protein domain(s); location	Score; E-value
OR1	51-1241	397	ORF1	hypothetical protein 1 [Yongsan tombus-like virus 1]; YP_009553260	85%; 69.21%	2e-158	**PLN02217** Probable pectinesterase 93..204	46.2; 2e-05
ORF2	1281-2717	479	ORF2	hypothetical protein 2 [Yongsan tombus-like virus 1]; YP_009553261	99%; 77.82%	0.0	**pfam00998:** RdRP_3, Viral RNA dependent RNA polymerase. 93..457	198; 7e-57
ORF3	2736 - 3959	408	ORF3	coat protein [Mosquito nodavirus MNV-1]; ACY74430	90%; 97.02%	0.0	**pfam11729:** Capsid-VNN, nodavirus capsid protein. 3..230	97.2; 2e-22

Supplementary File 1: References

1. McBride CS, Baier F, Omondi AB, Spitzer SA, Lutomiah J, Sang R, Ignell R, Vosshall LB. 2014. Evolution of mosquito preference for humans linked to an odorant receptor. Nature 515:222-7.
2. Poelchau MF, Reynolds JA, Denlinger DL, Elsik CG, Armbruster PA. 2013. Transcriptome sequencing as a platform to elucidate molecular components of the diapause response in the Asian tiger mosquito *Aedes albopictus*. Phys Entomol 38:173-181.
3. Poelchau MF, Reynolds JA, Elsik CG, Denlinger DL, Armbruster PA. 2013. Deep sequencing reveals complex mechanisms of diapause preparation in the invasive mosquito, *Aedes albopictus*. Proc Biol Sci 280:20130143.
4. Gamez S, Antoshechkin I, Mendez-Sanchez SC, Akbari OS. 2020. The Developmental Transcriptome of *Ae. albopictus*, a Major Worldwide Human Disease Vector. G3 10: 1051-1062.
5. Lewis SH, Quarles KA, Yang Y, Tanguy M, Frézal L, Smith SA, Sharma PP, Cordaux R, Gilbert C, Giraud I. 2017. Pan-arthropod analysis reveals somatic piRNAs as an ancestral defence against transposable elements. Nature Ecol Evol 2: 174-181.
6. Xu JB, Su XH, Bonizzoni M, Zhong DB, Li YJ, Zhou GF, Nguyen H, Tong S, Yan GY, Chen XG. 2018. Comparative transcriptome analysis and RNA interference reveal CYP6A8 and SNPs related to pyrethroid resistance in *Aedes albopictus*. PLoS Negl Trop Dis 12:e0006828.
7. Vedururu RK, Neave MJ, Sundaramoorthy V, Green D, Harper JA, Gorry PR, Duchemin JB, Paradkar PN. 2019. Whole Transcriptome Analysis of *Aedes albopictus* Mosquito Head and Thorax Post-Chikungunya Virus Infection. Pathogens 8: 132.
8. Vedururu RK, Neave MJ, Tachedjian M, Klein MJ, Gorry PR, Duchemin JB, Paradkar PN. 2019. RNASeq Analysis of *Aedes albopictus* Mosquito Midguts after Chikungunya Virus Infection. Viruses 11: 513.
9. Lombardo F, Salvemini M, Fiorillo C, Nolan T, Zwiebel LJ, Ribeiro JM, Arca B. 2017. Deciphering the olfactory repertoire of the tiger mosquito *Aedes albopictus*. BMC Genomics 18: 770.
10. Koh C, Allen SL, Herbert RI, McGraw EA, Chenoweth SF. 2018. The Transcriptional Response of *Aedes aegypti* with Variable Extrinsic Incubation Periods for Dengue Virus. Genome Biol Evol 10:3141-3151.
11. Etebari K, Hegde S, Saldana MA, Widen SG, Wood TG, Asgari S, Hughes GL. 2017. Global Transcriptome Analysis of *Aedes aegypti* Mosquitoes in Response to Zika Virus Infection. mSphere 2: e00456-17.
12. Xu Y, Dong Y, Xu Y, Lai Z, Jin B, Hao Y, Gao Y, Sun Y, Chen XG, Gu J. 2019. Differentiation of Long Non-Coding RNA and mRNA Expression Profiles in Male and Female *Aedes albopictus.* Front Genet 10:975.
13. Huang X, Poelchau MF, Armbruster PA. 2015. Global Transcriptional Dynamics of Diapause Induction in Non-Blood-Fed and Blood-Fed *Aedes albopictus*. PLoS Negl Trop Dis 9: e0003724.
14. Choi YJ, Aliota MT, Mayhew GF, Erickson SM, Christensen BM. 2014. Dual RNA-seq of parasite and host reveals gene expression dynamics during filarial worm-mosquito interactions. PLoS Negl Trop Dis 8:e2905.

Supplementary File 2: Annotations of the RNAi response in *Aedes* commensal viruses

Renna virus

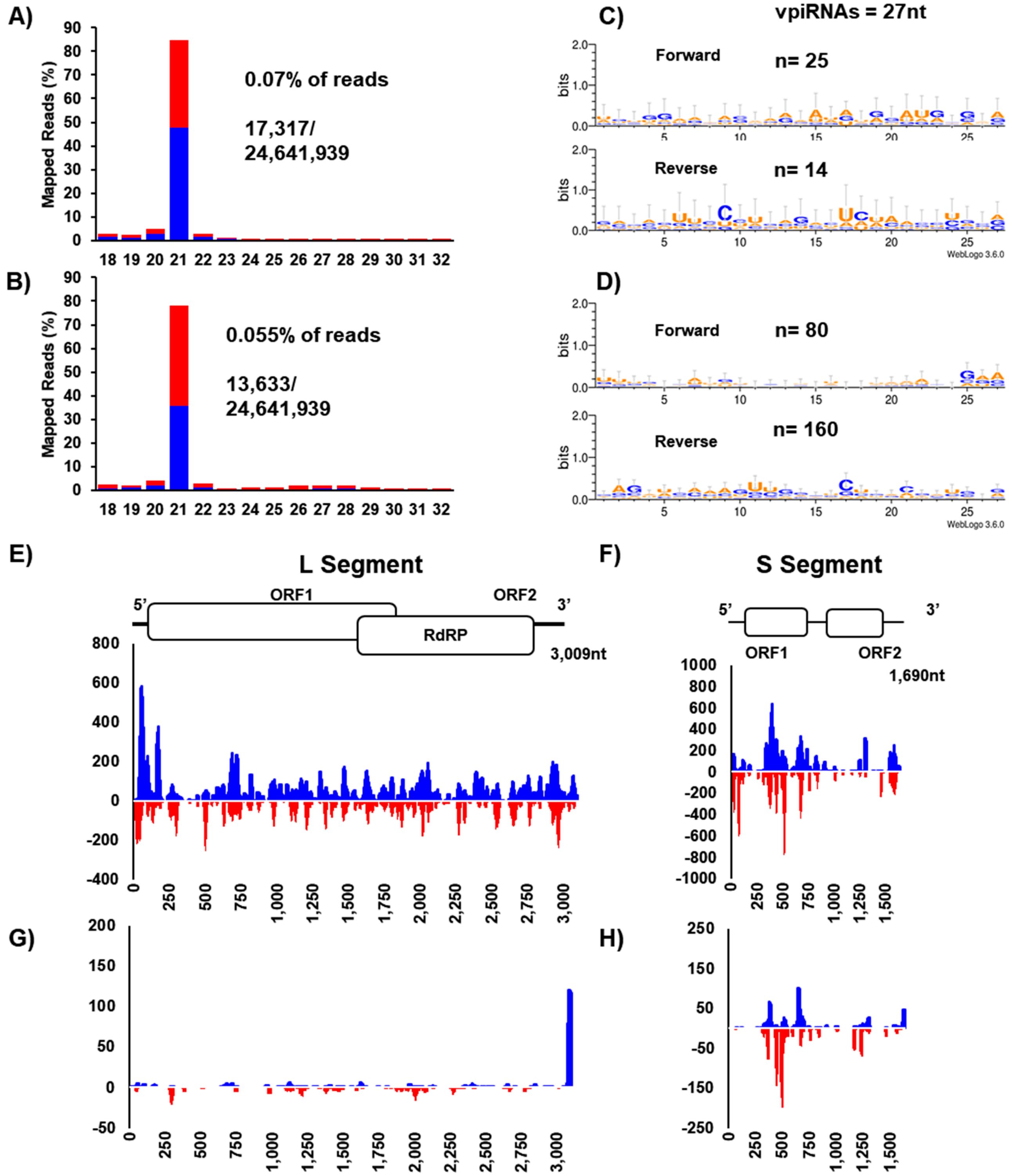

Figure S12. Virus-derived small RNA profiles and mapping coverage plots of Renna virus in *Ae. aegypti in vivo*. The bar graph represents the small-RNA reads mapping to the **A)** L segment and **B)** S segment of Renna virus Miami laboratory strain with genomic sense indicated in blue and anti-genomic reads mapped indicated in red. Representation of piRNA signature logos within 27-nt reads extracted mapping in the genome and anti-genome direction of both Renna virus L segment **C)** and **D)** S segment indicating that Renna virus does not produce vpiRNA length viral derived RNAs with a classical vpiRNA signature. Mapping profile of viral short interfering RNAs of 21nt in length to the **E)** L segment and **F)** S segment. Mapping profile of vpiRNAs 24-29nt mapping to the **G)** L segment and **H)** S segment. Small RNA sequencing data are taken from the Miami, USA *Ae. aegypti* colony originating from pooled soma and ovary libraries (BioProject Accession: PRJNA386859) (2).

Wenzhou Sobemo-like virus 4 (WSBV-4)

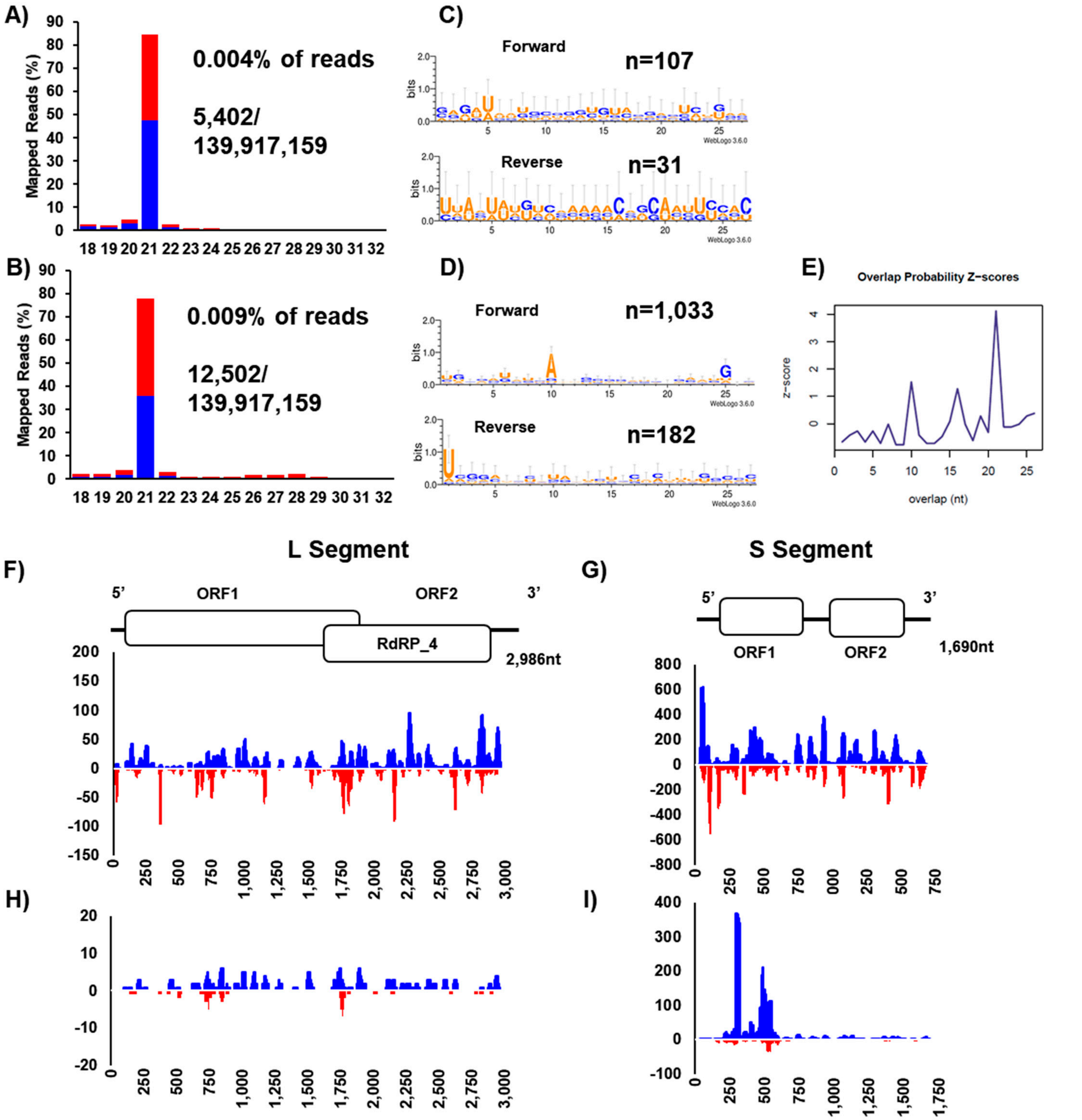

Figure S13. Virus-derived small RNA profiles and mapping coverage plots of Wenzhou Sobemo-like virus 4 (WSBLV-4) in *Ae. albopictus in vivo*. The bar graph represents the small-RNA reads mapping to the **A)** L segment and **B)** S segment of WSBLV-4 RML-12 cell line strain with genomic sense indicated in blue and anti-genomic reads mapped indicated in red. Representation of piRNA signature logos within 27-nt reads extracted mapping in the genome and anti-genome direction of both WSBLV-4 L segment **C)** and **D)** S segment indicating that WSBLV-4 produces vpiRNA length virus-derived RNAs with a classical vpiRNA signature for the S segment. **E)** Graphic representation of the position of identified 24-29nt overlapping small RNA pairs and associated z-scores. Mapping profile of vsiRNAs of 21nt in length to the **F)** L segment and **G)** S segment. Mapping profile of vpiRNAs 24-29nt mapping to the **H)** L segment and **I)** S segment. Small RNA sequencing data are taken from whole adult mosquitoes originating from the Phu Hoa, Ben Cat District, Binh Duong Province, Vietnam *Ae. albopictus* colony (BioProject accession: PRJNA293817) (3).

Wenzhou Sobemo-like virus 4 C-710 cells

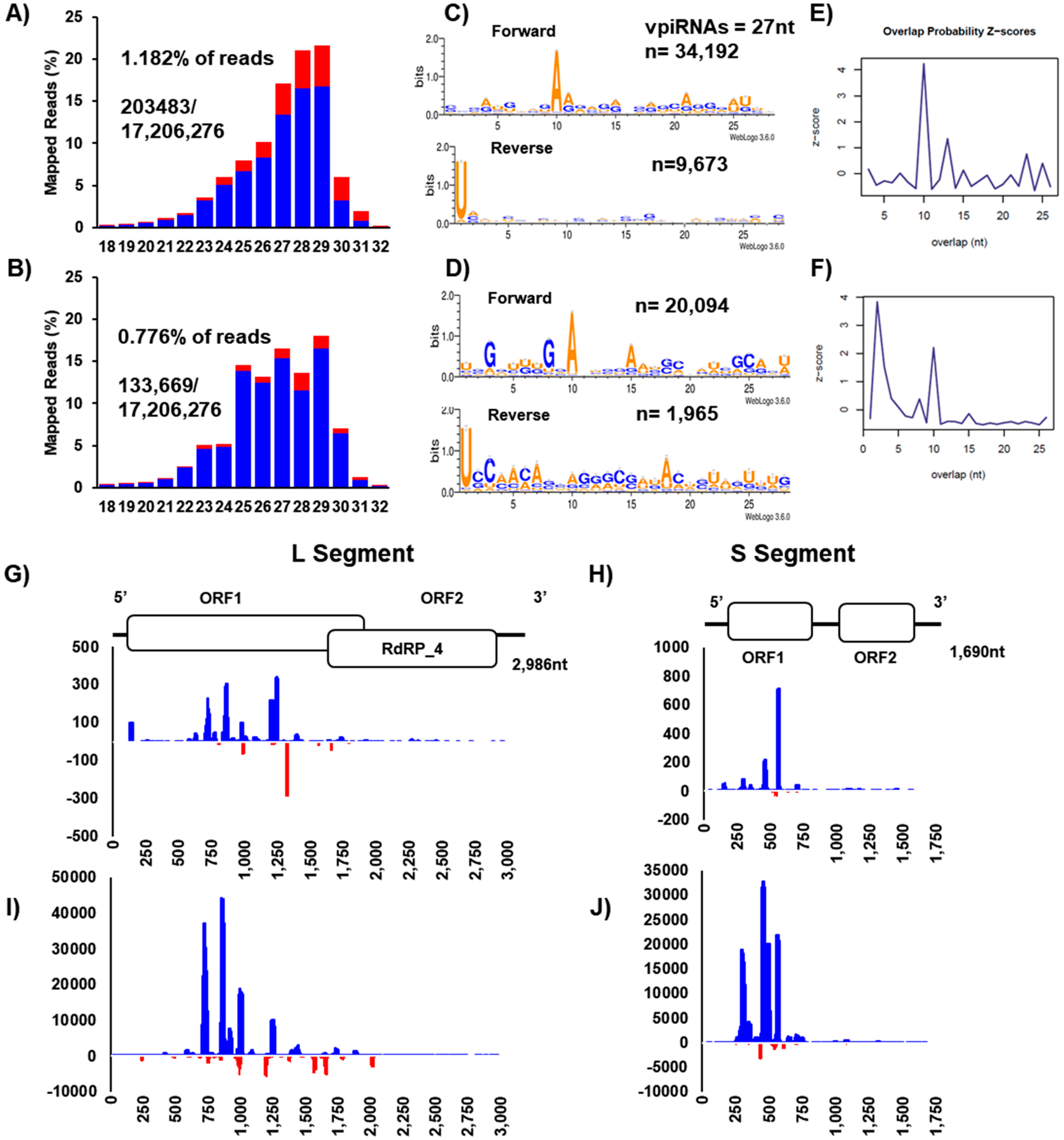

Figure S14. Virus-derived small RNA profiles and mapping coverage plots of Wenzhou Sobemo-like virus 4 (WSBLV-4) in *Ae. albopictus* C7-10 cells. The bar graph represents the small-RNA reads mapping to the **A)** L segment and **B)** S segment of WSBLV-4 RML-12 cell-line strain with genomic sense indicated in blue and anti-genomic reads mapped indicated in red. Representation of piRNA signature logos within 27-nt reads extracted mapping in the genome and anti-genome direction of both WSBLV-4 L segment **C)** and **D)** S segment indicating that WSBLV-4 produces vpiRNA length virus-derived RNAs with a classical vpiRNA signature for both segments. Graphic representation of the position of identified 24-29nt overlapping small RNA pairs and associated z-scores of the **E)** L segment and **F)** S segment. Mapping profile of vsiRNAs of 21nt in length to the **G)** L segment and **H)** S segment. Mapping profile of vpiRNAs 24-29nt mapping to the **I)** L segment and **J)** S segment. Small RNA sequencing data are taken from the *Ae. albopictus* C7-10 cell line (BioProject accession: PRJNA147359, SRA run: SRR346391) (1).

Aedes partiti-like virus 1 (APLV-1)

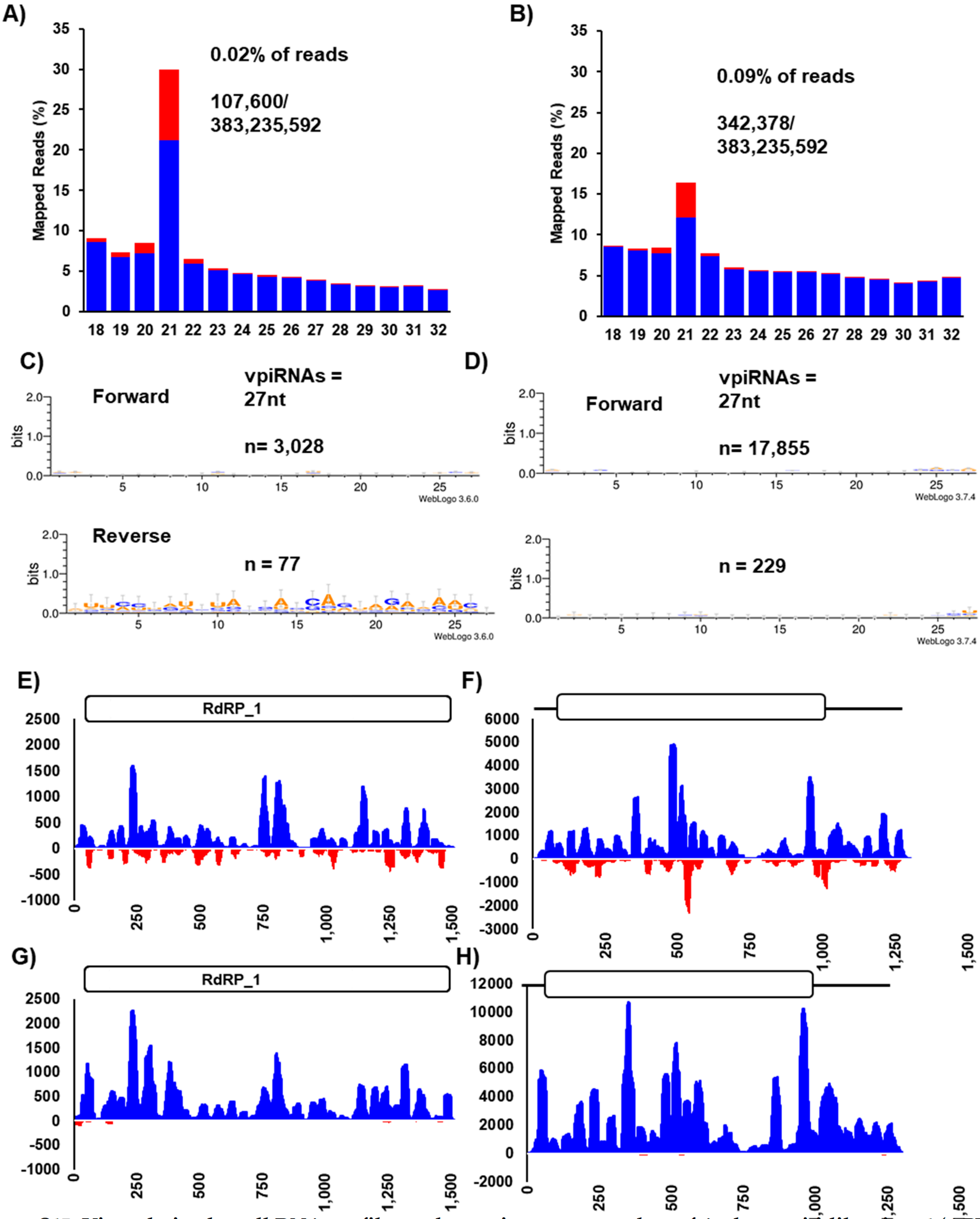

Figure S15. Virus-derived small RNA profiles and mapping coverage plots of Aedes partiti-like virus 1 (APLV-1) in *Ae. aegypti in vivo*. The bar graph represents the small-RNA reads mapping to the APLV-1 Galveston Strain with genomic sense indicated in blue and anti-genomic reads mapped shown in red against **A)** segment one and **B)** segment two. Representation of piRNA signature logos within 27-nt reads extracted mapping in the genome and anti-genome direction of **C)** segment one and **D)** segment 2. This indicates that APLV-1 does not produce vpiRNA length virus-derived RNAs with a classical vpiRNA signature. Mapping profile of vsiRNAs of 21nt in size against **E)** segment one and **F)** segment two. vpiRNAs 24-29nt mapping against **G)** segment one and **H)** segment two. Small RNA sequencing data are taken from the Galveston, USA *Ae. aegypti* colony from whole mosquitoes (BioProject accession: PRJNA382162) (4)

Verdadero virus

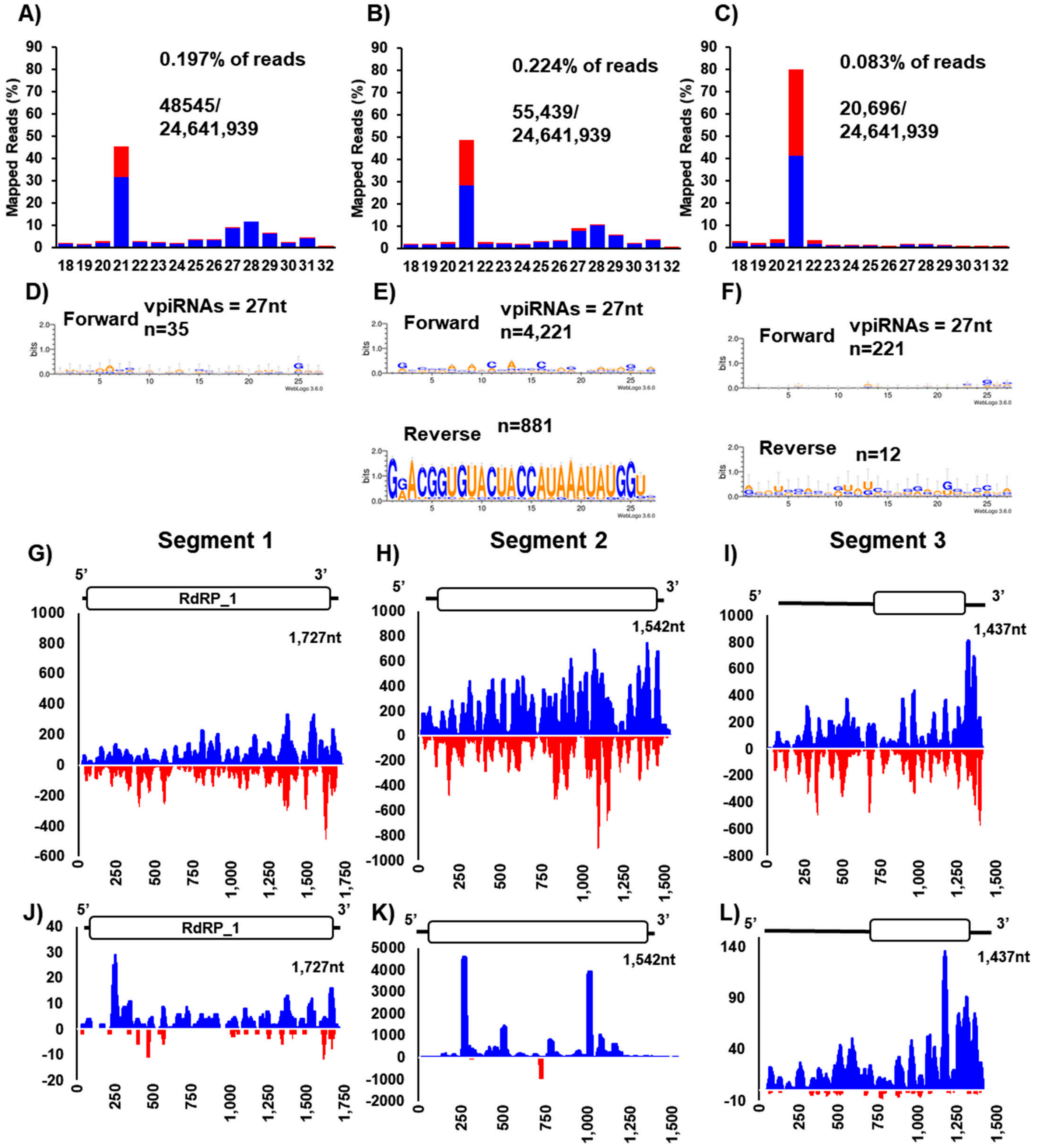

Figure S16. Virus-derived small RNA profiles and mapping coverage plots of Verdadero virus in *Ae. aegypti in vivo*. The bar graph represents the small-RNA reads mapping to the Verdadero virus Miami strain **A)** segment A **B)** segment B, and **C)** segment C with genomic sense indicated in blue and anti-genomic reads mapped indicated in red. **D-F)** Representation of piRNA signature logos within 27-nt reads extracted mapping in the genome and anti-genome direction indicating that Verdadero virus does not produce vpiRNA length virus-derived RNAs with a classical vpiRNA signature. **G-I)** Mapping profile of vsiRNAs of 21nt in size and **J-L)** vpiRNAs 24-29nt. Small RNA sequencing data are taken from the Miami, USA *Ae. aegypti* colony originating from pooled soma and ovary libraries (BioProject Accession: PRJNA386859) (2).

Aedes aegypti toti-like virus 1 (ATLV-1)

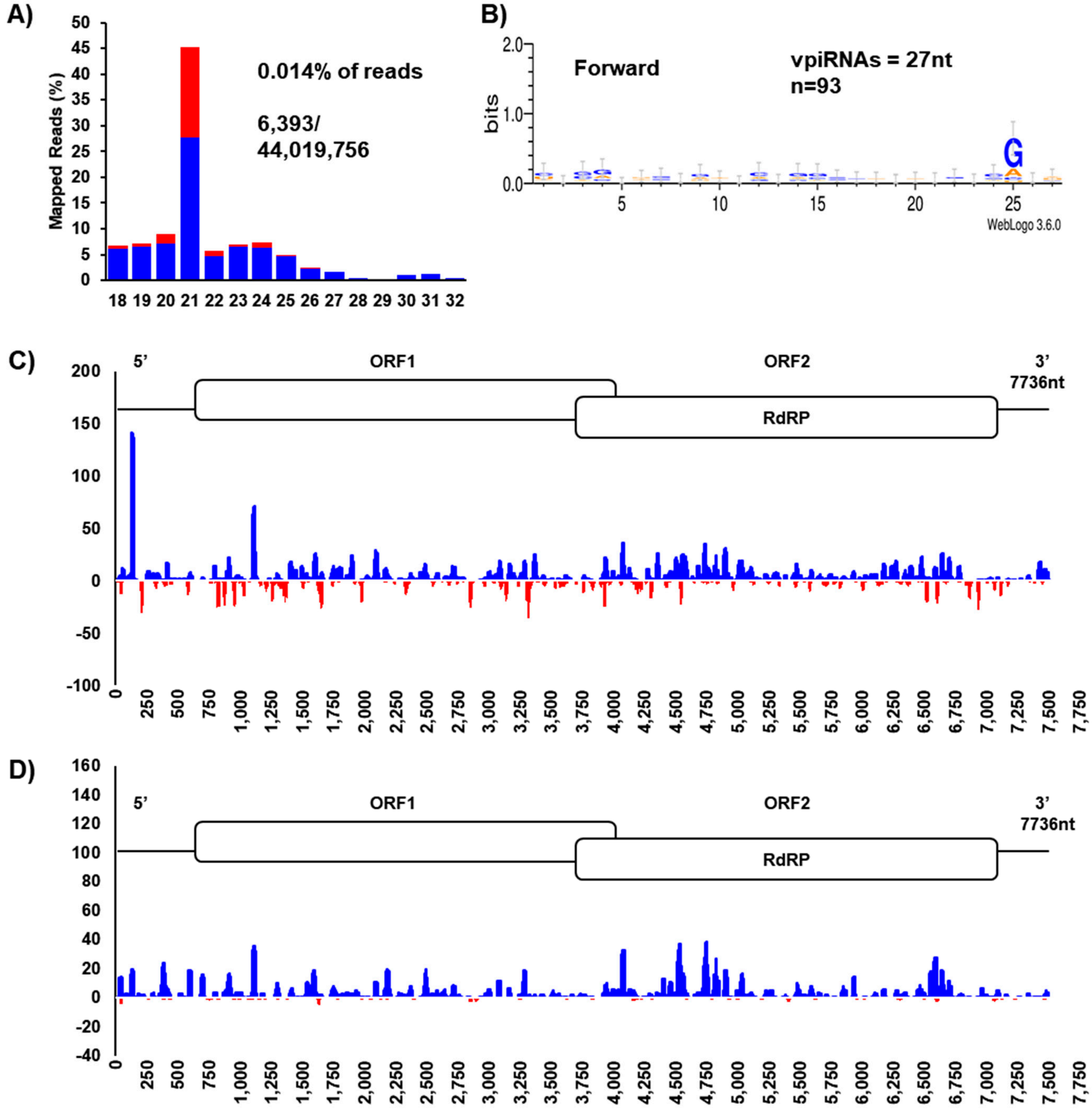

Figure S17. Virus-derived small RNA profiles and mapping coverage plots of Aedes aegypti toti-like virus 1 (ATLV-1) in *Ae. aegypti in vivo*. The bar graph represents the small-RNA reads mapping to the ATLV-1 Liverpool strain **A)** with genomic sense indicated in blue and anti-genomic reads mapped indicated in red. **B)** Representation of piRNA signature logos within 27-nt reads extracted mapping in the genome direction indicating that ATLV-1 does not produce vpiRNA length virus-derived RNAs with a classical vpiRNA signature. **C)** Mapping profile of vsiRNAs of 21nt in length and **D)** vpiRNAs 24-29nt. Small RNA sequencing data are taken from the Liverpool, *Ae. aegypti* colony originating from pooled life stages (BioProject Accession: PRJNA232374) (5).

Aedes aegypti toti virus (ATV)

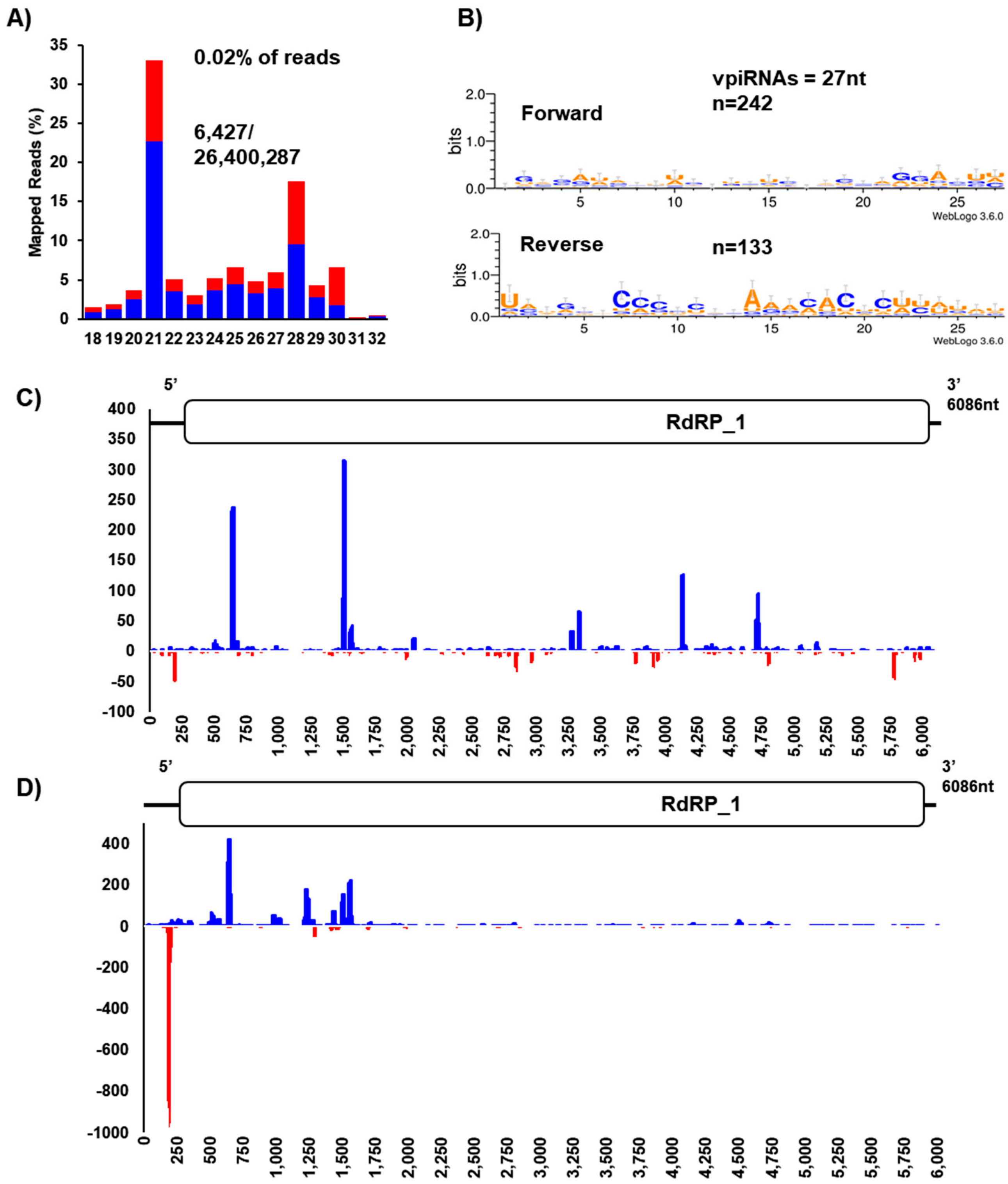

Figure S18. Virus-derived small RNA profiles and mapping coverage plots of Aedes aegypti toti virus (ATV) in *Ae. aegypti in vivo*. The bar graph represents the small-RNA reads mapping to the ATV Cairns, Australia strain **A)** with genomic sense indicated in blue and anti-genomic reads mapped indicated in red. **B)** Representation of piRNA signature logos within 27-nt reads extracted mapping in the genome and anti-genome direction indicating that ATLV-2 does not produce vpiRNA length virus-derived RNAs with a classical vpiRNA signature. **C)** Mapping profile of vsiRNAs of 21nt in length and **D)** vpiRNAs 24-29nt. Small RNA sequencing data are taken from whole adult mosquitoes originating from the Cairns, Australia *Ae. aegypti* colony (BioProject accession: PRJNA255495) (6).

Tiger mosquito bi-segmented tombus-like virus (TMTLV)

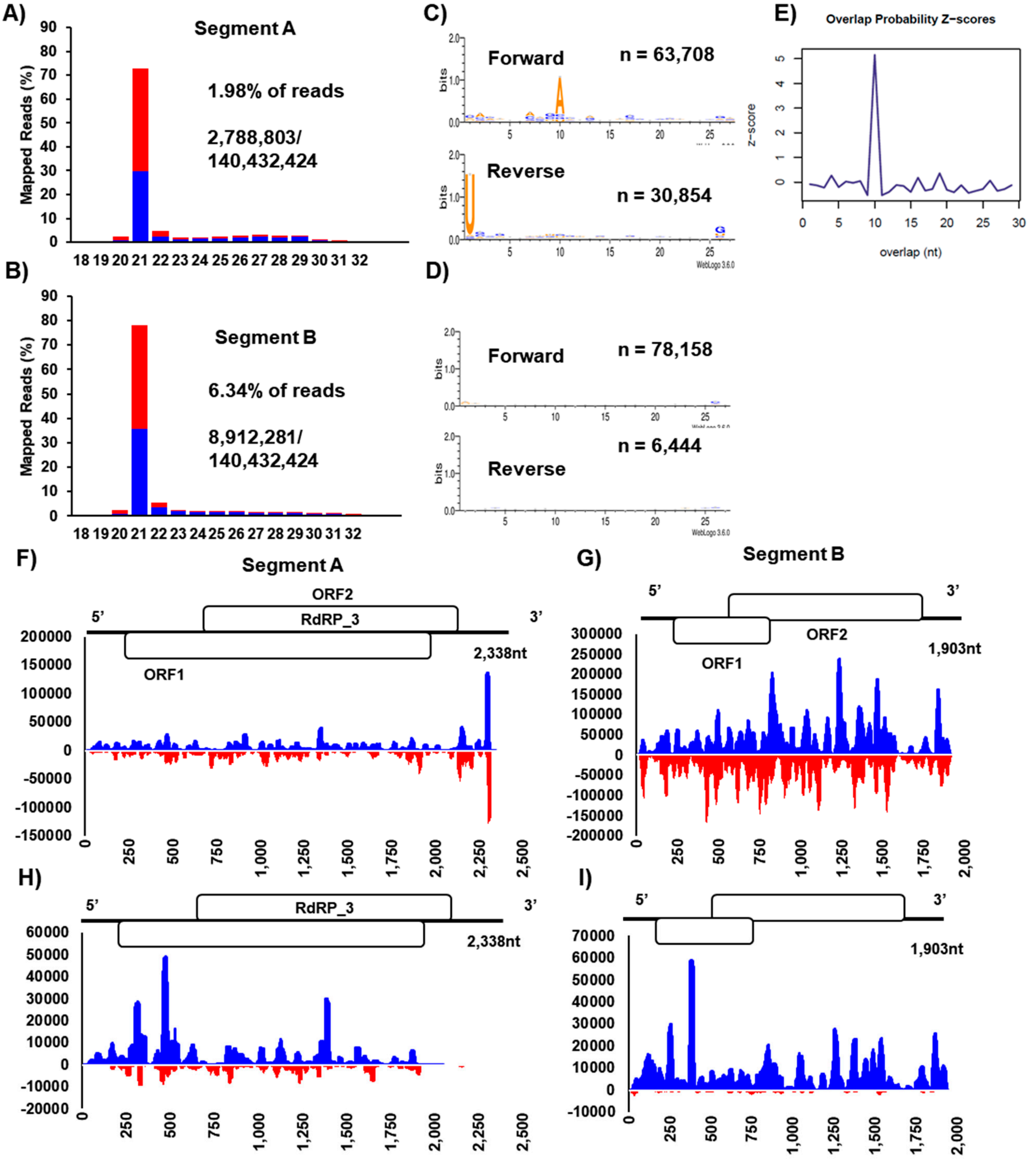

Figure S19. Virus-derived small RNA profiles and mapping coverage plots of Tiger mosquito bi-segmented tombus-like virus (TMTLV) in *Ae. albopictus in vivo*. The bar graph represents the small-RNA reads mapping to the **A)** A segment and **B)** B segment of TMTLV Phu Hoa strain with genomic sense indicated in blue and anti-genomic reads mapped indicated in red. Representation of piRNA signature logos within 27-nt reads extracted mapping in the genome and anti-genome direction of both TMTLV A segment **C)** and **D)** B segment indicating that TMTLV produces vpiRNA length virus-derived RNAs with a classical vpiRNA signature for the A segment. **E)** Graphic representation of overlap probability of identified 24-29nt small RNA pairs and associated z-scores for the A segment. Mapping profile of vsiRNAs of 21nt in length to the **F)** A segment and **G)** B segment. Mapping profile of vpiRNAs 24-29nt mapping to the **H)** A segment and **I)** B segment. Small RNA sequencing data are taken from whole adult mosquitoes originating from the Phu Hoa, Ben Cat District, Binh Duong Province, Vietnam *Ae. albopictus* colony (BioProject accession: PRJNA293817) (3).

Aedes orbi-like virus (AOLV)

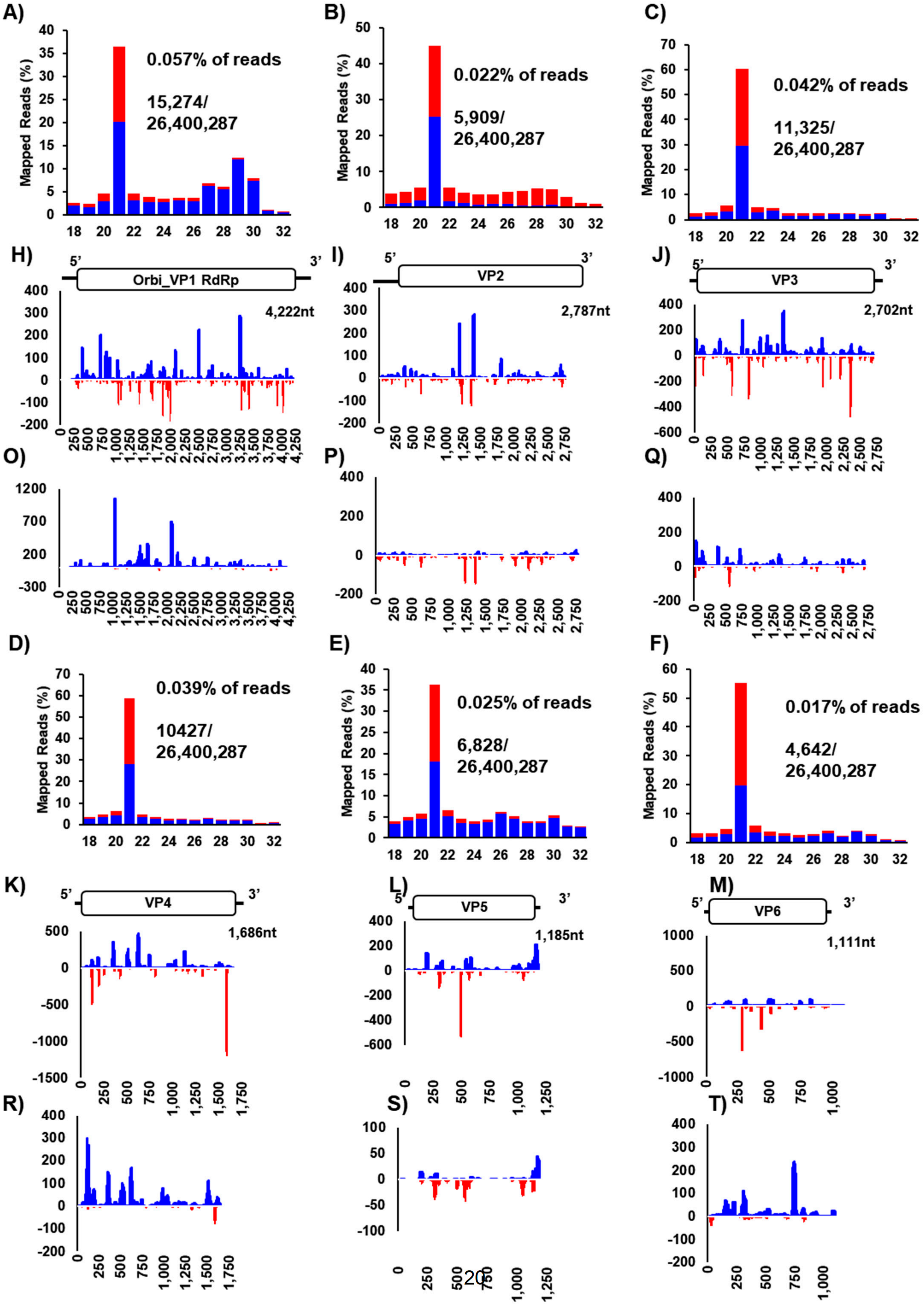

Aedes orbi-like (AOLV) Continued

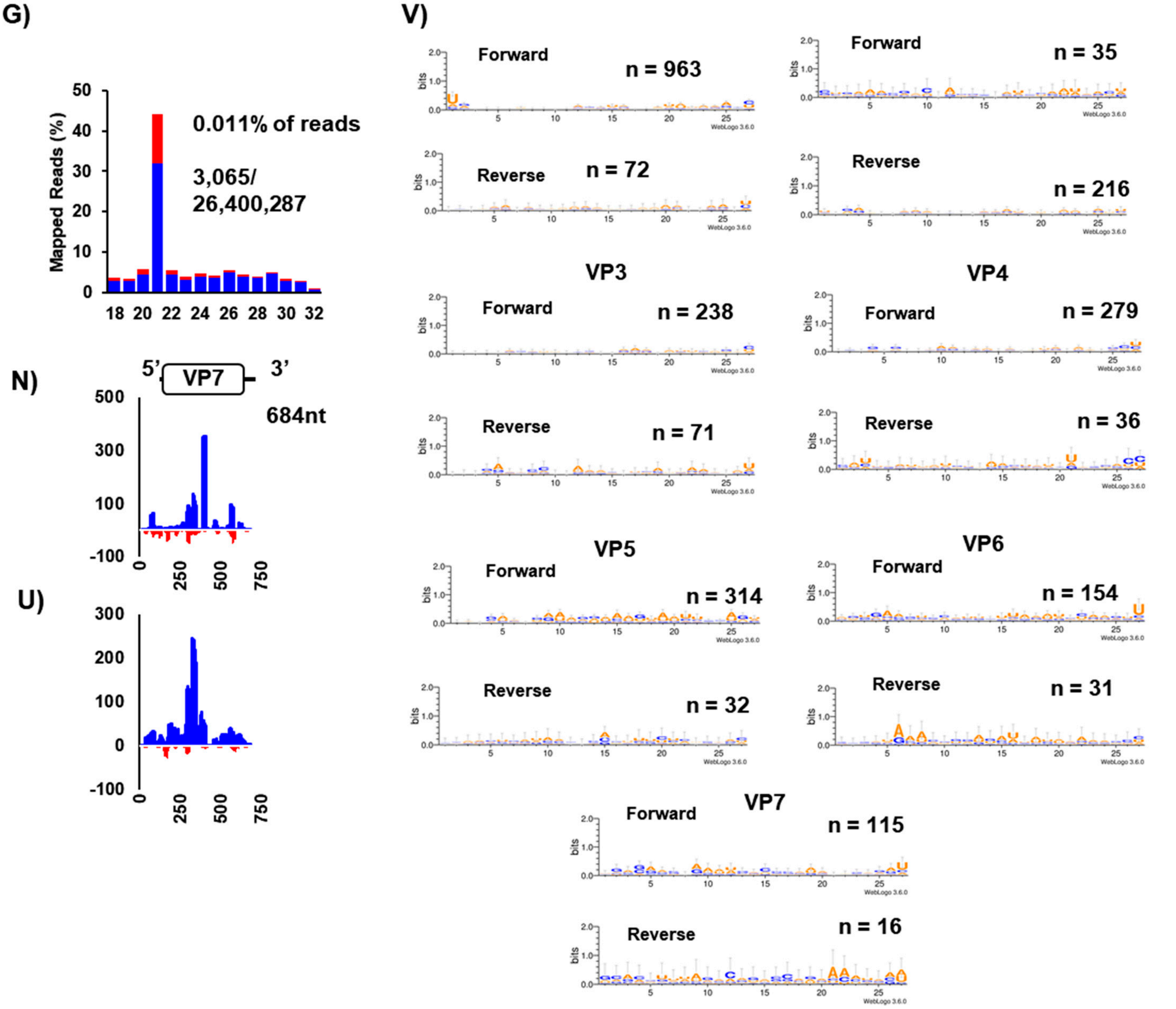

Figure S20. Virus-derived small RNA profiles and mapping coverage plots of Aedes orbi-like virus (AOLV) in *Ae. aegypti in vivo*. The bar graph represents the small-RNA reads mapping to the AOLV Cairns, Australia strain **A)** with genomic sense indicated in blue and anti-genomic reads mapped indicated in red to all 7 segments A-G. Mapping profile of vsiRNAs of 21nt in length **H-N)** vpiRNAs 24-29nt. **V)** Representation of piRNA signature logos within 27-nt reads extracted mapping in the genome and anti-genome direction indicating that AOV01 does not produce vpiRNA length virus-derived RNAs with a classical vpiRNA signature. Small RNA sequencing data are taken from the Cairns, Australia *Ae. aegypti* colony originating from whole blood-fed adult mosquitoes (BioProject accession: PRJNA255495) (6).

Aedes binegev-like virus 1 (AeBNV-1)

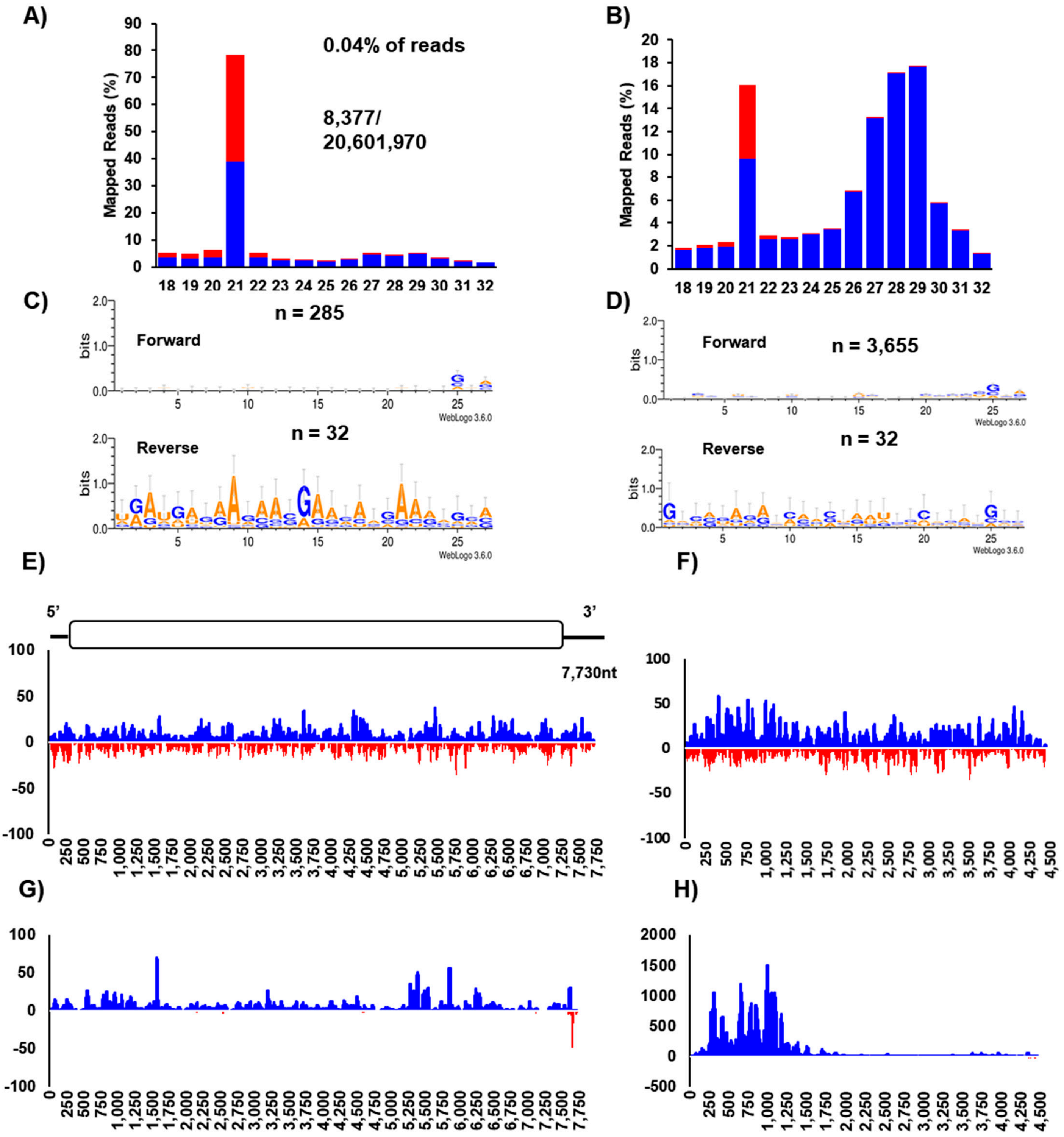

Figure S21. Virus-derived small RNA profiles and mapping coverage plots of Aedes binegev like virus 1 (AeBNV-1) in *Ae. aegypti in vivo*. The bar graph represents the small-RNA reads mapping to the **A)** L segment and **B)** S segment of AeBNV-1 Miami laboratory strain with genomic sense indicated in blue and anti-genomic reads mapped indicated in red. Representation of piRNA signature logos within 27-nt reads extracted mapping in the genome and anti-genome direction of both AeBNV-1 L segment **C)** and **D)** S segment indicating that AeBNV-1 does not produce vpiRNA length virus-derived RNAs with a classical vpiRNA signature. Mapping profile of vsiRNAs of 21nt in length to the **E)** L segment and **F)** S segment. Mapping profile of vpiRNAs 24-29nt mapping to the **G)** L segment and **H)** S segment. Small RNA sequencing data are taken from the Miami, USA *Ae. aegypti* colony originating from pooled soma and ovary libraries (Bioproject Accession: PRJNA386859) (2).

Liverpool tombus-like virus (previously Mosquito Nodavirus)

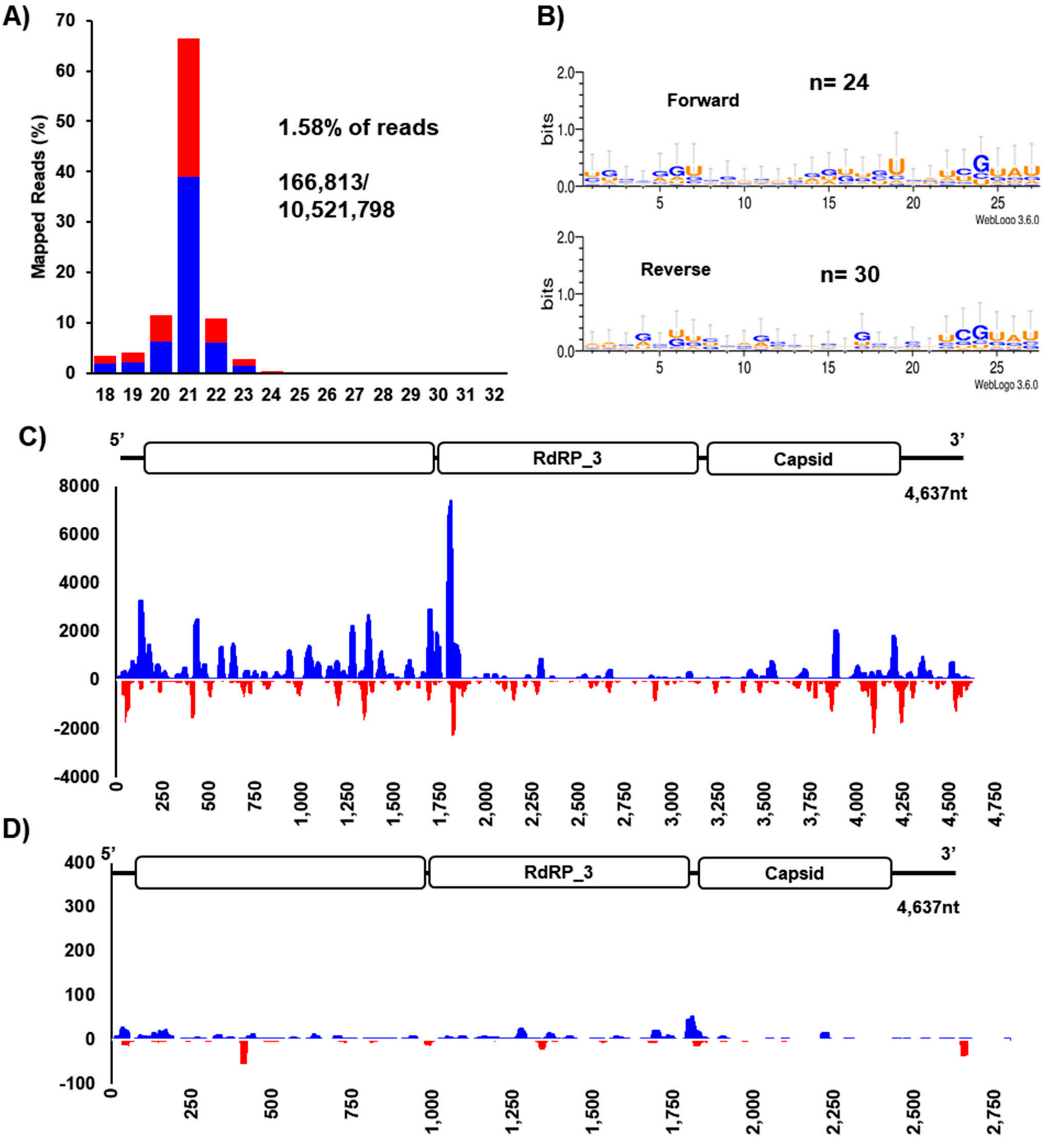

Figure S22. Virus-derived small RNA profiles and mapping coverage plots of Liverpool tombus-like virus (LPTV) in *Ae. aegypti in vivo*. The bar graph represents the small-RNA reads mapping to the LPTV genome **A)** with genomic sense indicated in blue and anti-genomic reads mapped indicated in red. **B)** Representation of piRNA signature logos within 27-nt reads extracted mapping in the genome direction indicating that LPTV does not produce vpiRNA length virus-derived RNAs with a classical vpiRNA signature. **C)** Mapping profile of vsiRNAs of 21nt in length, and **D)** vpiRNAs 24-29nt. Small RNA sequencing data are taken from the Liverpool, *Ae. aegypti* colony originating adult samples (BioProject accession: PRJNA150789)(7).

Yongsan tombus-like virus 1 (YTLV) - Foshan Strain

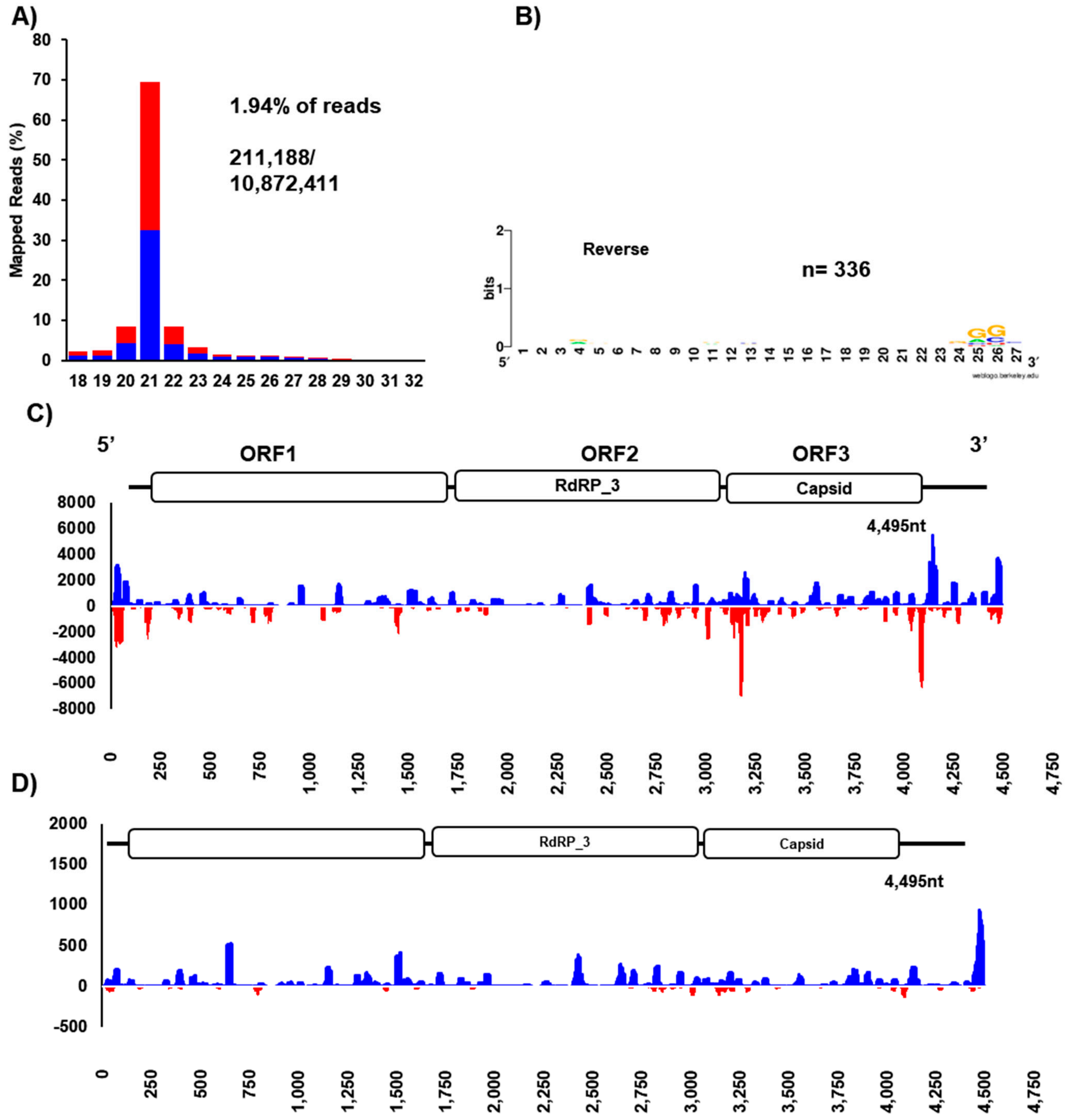

Figure S23. Virus-derived small RNA profiles and mapping coverage plots of Yongsan tombus-like virus 1 (YTLV) in *Ae. albopictus in vivo*. The bar graph represents the small-RNA reads mapping to the YTLV genome. **A)** with genomic sense indicated in blue and anti-genomic reads mapped indicated in red. **B)** Representation of piRNA signature logos within 27-nt reads extracted mapping in the anti-genome (reverse) direction indicating that YTLV does not produce vpiRNA length virus-derived RNAs with a classical vpiRNA signature. **C)** Mapping profile of vsiRNAs of 21nt in length, and **D)** vpiRNAs 24-29nt. Small RNA sequencing data are taken from the Foshan, *Ae. albopictus* colony (BioProject accession: PRJNA236401)(8).

Aedes phasma virus (APV)

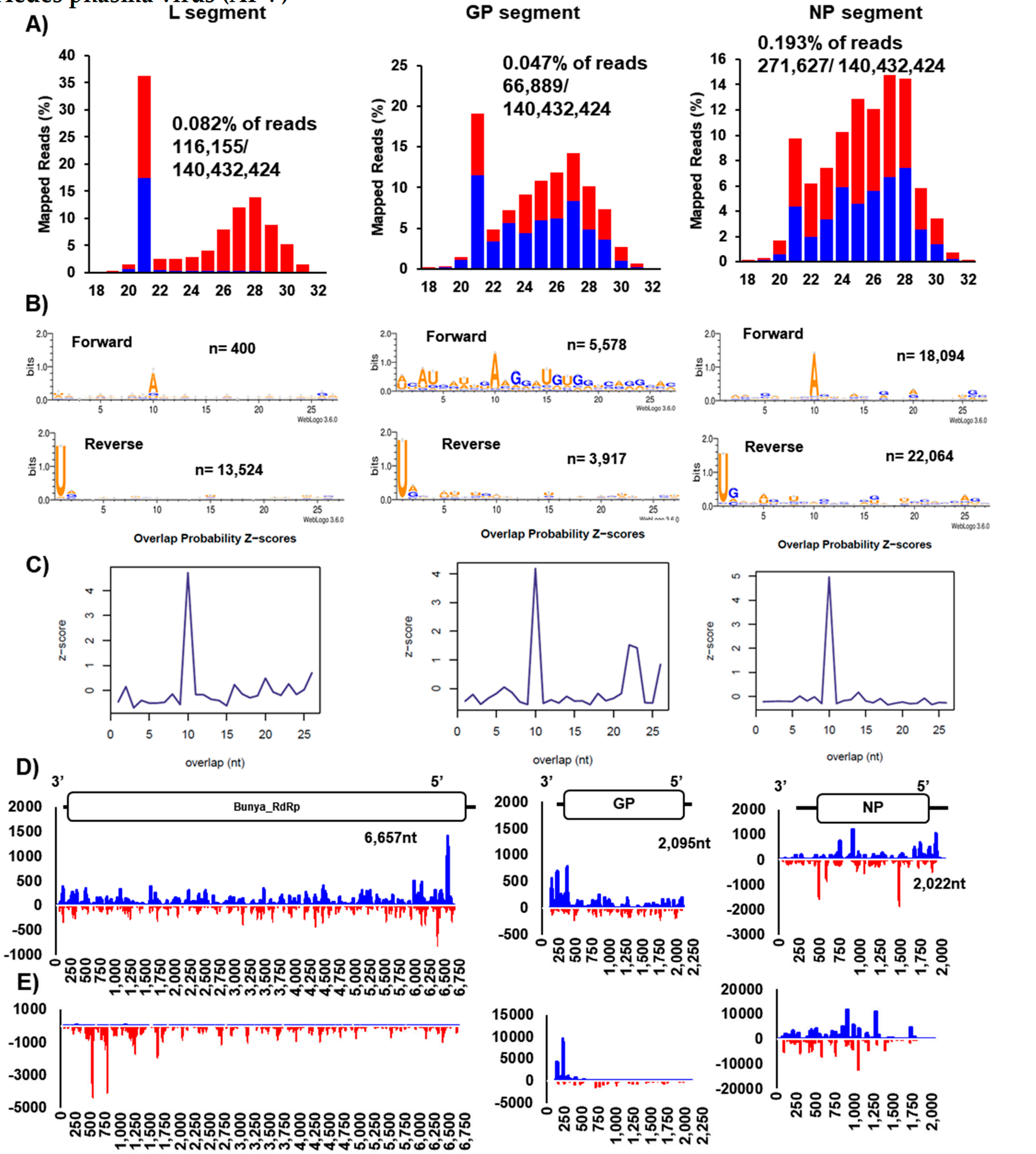

Figure S24. Virus-derived small RNA profiles and mapping coverage plots of Aedes phasma virus (APV) in *Ae. albopictus in vivo*. The bar graph represents the small-RNA reads mapping to **A)** the three genome segments of APV Phu Hoa strain with genomic sense indicated in blue and anti-genomic reads mapped shown in red. Representation of piRNA signature logos **B)** within 27-nt reads extracted mapping in the genome and anti-genome direction of the three segments of APV. Graphic representation of overlap probability **C)** of identified 24-29nt small RNA pairs and associated z-scores for the three segments. **D)** Mapping profile of vsiRNAs of 21nt in length to the three genome segments. Mapping profile of vpiRNAs (24-29nt) **E)** mapping to the three segments of APV. The small RNA sequencing data are taken from whole adult mosquitoes originating from the Phu Hoa, Ben Cat District, Binh Duong Province, Vietnam *Ae. albopictus* colony (BioProject accession: PRJNA293817) (3).

Guadeloupe mosquito quaranja-like virus 1 (GMQLV-1)

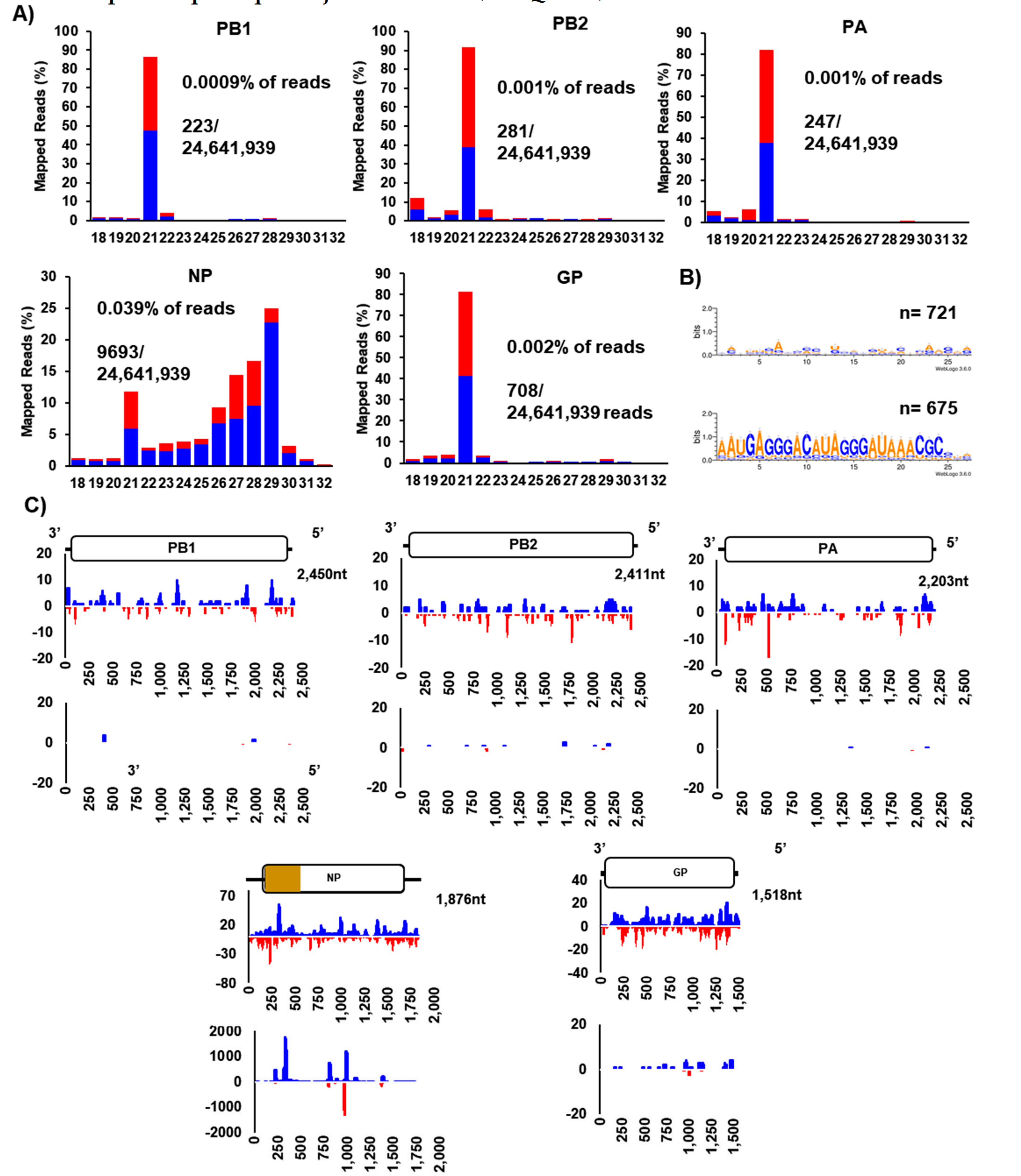

Figure S25. Virus-derived small RNA profiles and mapping coverage plots of Guadeloupe mosquito quaranja-like virus 1 (GMQLV-1) in *Ae. aegypti in vivo*. A) The bar graph represents the small-RNA reads mapping to the GMQLV-1 Miami, Laboratory strain with genomic sense indicated in blue and anti-genomic reads mapped indicated in red to all 5 segments PB1, PB2, PA, NP, GP **B)** Representation of piRNA signature logos within 27-nt reads extracted mapping in the genome, and anti-genome direction of the NP segment indicates that GMQLV-1 does not produce vpiRNA length virus-derived RNAs with a classical vpiRNA signature. **C)** Mapping profile of vsiRNAs of 21nt in length and vpiRNAs 24-29nt. Small RNA sequencing data are taken from the Miami, USA *Ae. aegypti* colony originating from pooled soma and ovary libraries (BioProject Accession: PRJNA386859) (2).

Culex pipiens pallens densovirus (CppDNV)

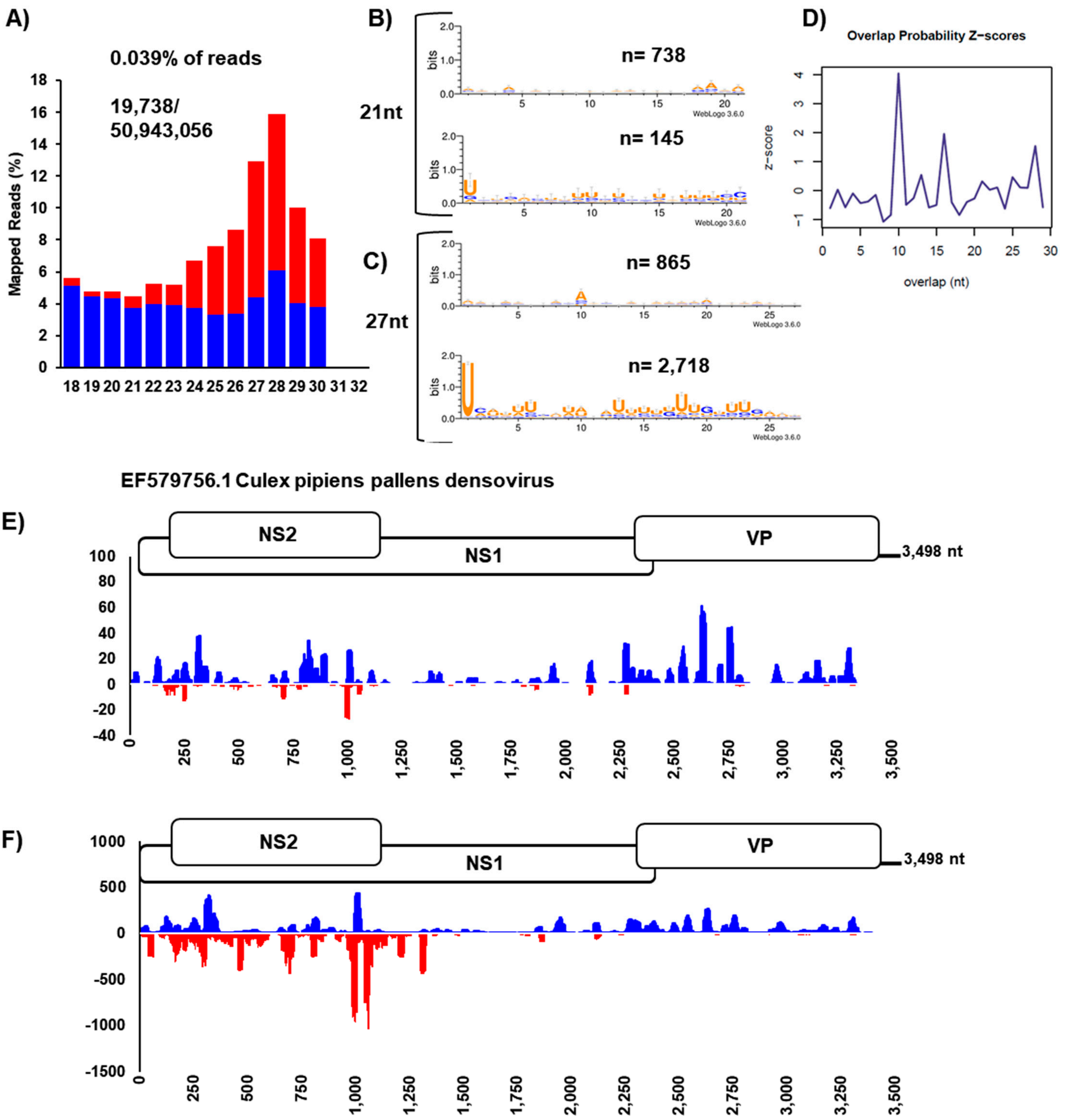

Figure S26. Virus-derived small RNA profiles and mapping coverage plots of Culex pipiens pallens densovirus (CppDNV) in *Ae. albopictus* C6/36 cells. A) The bar graph represents the small-RNA reads mapping to the CppDNV strain available on Genbank EF579756.1 with genomic sense indicated in blue and anti-genomic reads mapped indicated in red. Representation of piRNA signature logos within extracted **B)** 21nt and **C)** 27-nt reads mapping to the genome and anti-genome direction of the genome indicating that CppDNV produces vpiRNA length virus-derived RNAs with a classical vpiRNA signature. **D)** Graphical representation of the position of identified 21-29nt overlapping small RNA pairs and associated z-scores. **E)** Mapping profile of vsiRNAs of 21nt in length. **F)** Mapping profile of vpiRNAs 24-29nt mapping to the genome. Small RNA sequencing data are taken from the *Ae. albopictus* C6/36 cell line (BioProject accession: PRJNA374578)(9).

Supplementary File 2: References

1. Morazzani EM, Wiley MR, Murreddu MG, Adelman ZN, Myles KM. 2012. Production of virus-derived ping-pong-dependent piRNA-like small RNAs in the mosquito soma. PLoS Pathog 8:e1002470.
2. Lewis SH, Quarles KA, Yang Y, Tanguy M, Frézal L, Smith SA, Sharma PP, Cordaux R, Gilbert C, Giraud I. 2017. Pan-arthropod analysis reveals somatic piRNAs as an ancestral defence against transposable elements. Nat Ecol Evol:2: 174-181.
3. Goic B, Stapleford KA, Frangeul L, Doucet AJ, Gausson V, Blanc H, Schemmel-Jofre N, Cristofari G, Lambrechts L, Vignuzzi M, Saleh MC. 2016. Virus-derived DNA drives mosquito vector tolerance to arboviral infection. Nat Commun 7:12410.
4. Saldana MA, Etebari K, Hart CE, Widen SG, Wood TG, Thangamani S, Asgari S, Hughes GL. 2017. Zika virus alters the microRNA expression profile and elicits an RNAi response in Aedes aegypti mosquitoes. PLoS Negl Trop Dis 11:e0005760.
5. Hu W, Criscione F, Liang S, Tu Z. 2015. MicroRNAs of two medically important mosquito species: *Aedes aegypti* and *Anopheles stephensi.* Insect Mol Biol 24:240-52.
6. Etebari K, Osei-Amo S, Blomberg SP, Asgari S. 2015. Dengue virus infection alters post-transcriptional modification of microRNAs in the mosquito vector *Aedes aegypti*. Sci Rep 5:15968.
7. Adelman ZN, Anderson MA, Liu M, Zhang L, Myles KM. 2012. Sindbis virus induces the production of a novel class of endogenous siRNAs in *Aedes aegypti* mosquitoes. Insect Mol Biol 21:357-68.
8. Liu YX, Zhou YH, Wu JY, Zheng PM, Li YJ, Zheng XY, Puthiyakunnon S, Tu ZJ, Chen XG. 2015. The expression profile of *Aedes albopictus* miRNAs is altered by dengue virus serotype-2 infection. Cell Biosci 5: 16.
9. Avila-Bonilla RG, Yocupicio-Monroy M, Marchat LA, De Nova-Ocampo MA, del Angel RM, Salas-Benito JS. 2017. Analysis of the miRNA profile in C6/36 cells persistently infected with dengue virus type 2. Virus Res 232:139-151.
