# Supplementary material for: Uncovering the Worldwide Diversity and Evolution of the Virome of the Mosquitoes Aedes aegypti and Aedes albopictus
Source: Microorganisms. 2021 Aug 3;9(8):1653. doi: 10.3390/microorganisms9081653 (PMC8398489; doi:10.3390/microorganisms9081653)
Supplement: Supplementary file 1 [file microorganisms-09-01653-s001.zip › Supplementary Tables_proof.pdf]

Table S1. All *Ae. aegypti* samples analysed in this study and associated publications

Colony designation, City and country of original catch and Accession number	Date of original catch/<br>Date and generation (g) of sequencing	Wild-caught/ Field derived strain/<br>Laboratory strain	Total-RNA or sRNA sequencing	Tissues sequenced	REF
**African Samples**					
Bakoumba, Gabon<br>BioProject accession: PRJNA386453	Wild-caught: ~2014<br>Deposited in SRA: 2017-05-12	Field derived strain	**mRNA, polyA enriched**	Individual midgut libraries or whole mosquitoes	(1)
Rabai, Kenya (K2, K4, K14, K18, K19, K27)<br>BioProject accession: PRJNA233429	Collected: January 2009<br>Sequencing: 2010-2011, g5/g8<br>Deposited in SRA: 2014-01-04	Field derived strain	**mRNA, polyA enriched**<br>Illumina TruSeq RNA Sample Prep Kit v. 2 and mRNA Sequencing Sample Prep Kit.	Antenna	(2)
Rabai, Kenya (GP1, GP2, HP1, HP2)<br>BioProject accession: PRJNA233429	Collected: January 2009<br>Sequenced: 2010-2011, F2 of K27(G5) and K14(G5) cross<br>Deposited in SRA: 2014-01-04	Field derived strain	**mRNA, polyA enriched**<br>As above	Antenna	(2)
Shauri Moyo (Moyo-S/R), Mombasa, Kenya<br>BioProject accession: PRJNA396016	Collected: ~1980s<br>Deposited in SRA: 2017-07-27	Lab strain	**sRNA sequencing**<br>Epicentre ScriptMiner kit (Cat. No. SMMP101212)	Second, third, fourth instar larvae and adults	(3)
Bundibugyo (U30), Uganda<br>BioProject accession: PRJNA233429	Collected: January 2009<br>Sequenced: 2010-2011, g3<br>Deposited in SRA: 2014-01-04	Field derived strain	**mRNA, polyA enriched**<br>As above	Antenna	(2)
Wakiso, Uganda<br>BioProject accession: PRJNA556544	Collected: 2016<br>Deposited in SRA: 2019-07-25	Mosquito homogenates passaged on c6/36 cells.	**ViralRNA-** RNA extracted using QIAamp Viral RNA Mini Kit (Qiagen)<br>Library preparation using Nextera XT DNA Kit (Illumina)	Cell culture isolated virus from a laboratory colony of *Aedes aegypti* homogenised and passaged in c6/36 cells 2 passages.	(4)
**Australian / Asian Samples**					
Cairns, Queensland, Australia<br>BioProject accession: PRJNA413709	Collected: March-June 2014<br>Deposited in SRA: 2017-10-09	Wild pools	**Total-RNA, Ribosomal-RNA depleted**	Pooled whole mosquitoes	(5)
Cairns, Queensland, Australia<br>Available on FigShare: https://doi.org/10.26180/5bb0d5ae69035	Collected in Cairns in March 2014, continually maintained	Field derived strain	**mRNA, polyA enriched**<br>Illumina TruSeq Stranded mRNA-Seq	Pooled whole mosquitoes	(6)
North Queensland Strain, Australia<br>BioProject accession: PRJNA255495	Origin: Cairns<br>Collected prior to 2013<br>Deposited in GEO: 2014-07-17	Field derived strain	**sRNA sequencing**<br>Illumina TruSeq Small RNA	Pooled whole mosquitoes	(7)
Innisfail, North Queensland, Australia<br>BioProject accession: PRJNA630779	Collected: 2016-04<br>Deposited in SRA: 2020-05-06	Field derived strain	Poly(A) mRNA isolation & Ribo-Zero rRNA removal Kit (Illumina)	Female adult bodies with wings and legs removed (n=6)	(8)
Innisfail North Queensland, Australia<br>BioProject accession: PRJNA545086	Wild collected April 2017, and the F3-5 generations were used for the experiments.<br>Deposited in SRA: May 28, 2019	Field derived strain	RNA depletion using Ribo-Zero Gold rRNA Removal Kit (Illumina, USA) followed by library preparation using TruSeq 2 Library Preparation Kit	Pooled female adult mosquitoes.	(9)
Townsville, Queensland, Australia<br>GEO accession: GSE100917	Wild Collected: March 2015<br>Sequenced: June/July 2015<br>Deposited in GEO: 2017-07-07	Field derived strain	**sRNA sequencing**<br>Illumina TruSeq Small RNA	Pooled whole mosquitoes	(10)
Kampong Cham, Cambodia<br>BioProject: PRJNA556544	Single lab colony female: 2013<br>Single wild-caught female: 2013<br>Cell culture homogenates: 2015<br>Deposited in SRA: 2019-07-25	Mosquito homogenates passaged on c6/36 cells, and single mosquitoes used for sequencing.	ViralRNA<br>RNA extracted using QIAamp Viral RNA Mini Kit (Qiagen)<br>Library preparation using Nextera XT DNA Kit (Illumina)	Cell culture isolated virus from a laboratory colony of *Aedes aegypti* after 3 passages.<br>Two individual mosquitoes: one wild-caught and one from the colony	(4)
Phnom Penh, Cambodia<br>BioProject: PRJNA556544<br>SRA accession: SRR9830742	Cell culture homogenates: 2015<br>Deposited in SRA: 2019-07-25	Mosquito homogenates passaged on c6/36 cells		Cell culture isolated virus from laboratory colony of *Aedes aegypti* after 2 passages.	
Fuzhou, Fujian province, China<br>BioProject accession: PRJNA476690	Samples collected 2015,<br>Deposited in SRA: 2018-06-19	Unknown origin	Total-RNA	Midgut	(11)
Bora-Bora, French Polynesia<br>BioProject accession: PRJEB15531	~Caught in 1990,<br>Deposited in SRA: 2016-12-29	Field derived strain	TruSeq Stranded mRNA sample Prep kit	Whole non-blood fed adult females	(12)
Bora-Bora, French Polynesia<br>BioProject accession: PRJEB1859	~Caught in 1990<br>RNA-Seq produced ~2010<br>Deposited in SRA: 2013-09-02	Field derived strain	Total-RNA	Larval midguts	(13)
Bora-Bora, French Polynesia<br>BioProject accession: PRJEB1859	~Caught in 1990<br>RNA-Seq produced ~2013<br>Deposited in SRA: 2013-09-02	Field derived strain	Total-RNA, polyA enriched	G_{11} larvae obtained from three independent egg batches Selected for insecticide resistance	(14)
Bora-Bora, French Polynesia<br>BioProject accession: PRJEB32622	~Caught in 1990<br>Deposited in GEO: 2019-05-17	Field derived strain	Total-RNA, polyA enriched	Whole larval pooled samples	(15)
Toahotu, Tahiti, French Polynesia<br>BioProject accession: PRJNA382618	Collected 2014-2015<br>Sequencing: ~2016, 9th-14th generation<br>Deposited in SRA: 2017-04-12	Field derived strain	Total-RNA	Pooled saliva samples	(16)
New Delhi India<br>Sequencing available from Figshare URL: https://figshare.com/s/ce9f231e8a4dde617722	Unknown	Unknown	Total-RNA	Whole mosquitoes	(17)
Xayaboury, Luang Prabang, Vientiane Capital, Saravane, Attapeu<br>Laos<br>BioProject accession: PRJEB37991	Collected: 2014<br>Fifth generation lab colony subjected to sequencing<br>Deposited in SRA: 2020-05-01	Field derived strain	Oligo-dT selection of total-RNA<br>NEBNext® UltraTM II directional RNA library Prep Kit for Illumina (New England Biolabs)	Whole female 3-days old adult non-blood fed	(18)
Jinjang, Selangor, Malaysia<br>BioProject accession: PRJNA320356	Field caught 1975<br>Deposited in SRA: 2016-05-19	Lab strain	Total-RNA, Poly-A enriched	Germline	(19)
Bangkok, Thailand<br>BioProject accession: PRJNA413709	Wild-caught: June 2015<br>Deposited in SRA: 2017-10-09	Wild pools	Total-RNA, Ribosomal-RNA depleted	Whole mosquitoes	(5)
Bangkok, Thailand<br>BioProject accession: PRJNA612326	Wild-caught: 2009<br>Deposited in SRA: 2020-03-12	Lab strain	PolyA enriched<br>NEBNext Poly(A) mRNA Magnetic Isolation kit	Female spermathecae of blood fed and non-blood fed adults.	(20)

Kamphaeng Phet, Thailand<br>BioProject accession: PRJNA310013	Wild-caught in 2011, supplemented with mosquitoes every 2-3 years<br>Sequencing conducted in October 2013 and January 2014<br>Deposited in SRA: 2016-01-27	Lab strain	Total-RNA	Female reproductive organs	(21)
Kamphaeng Phet, Thailand<br>BioProject accession: PRJNA487089	As above:<br>Sequencing conducted: June and August of 2017<br>Deposited in SRA: 2018-08-21	Lab strain	Total-RNA	Male reproductive organs	(22)
Kamphaeng Phet Province, Thailand<br>BioProject accession: PRJNA482553	Deposited in SRA: 2018-07-12	Field derived strain (26 generations)	mRNA	Embryos, engorged female mosquitoes	(23)
Phetchaburi, Thailand<br>BioProject accession: PRJEB15531	Collected during 2014-2015<br>Sequenced: Using G2<br>Deposited in SRA: 2016-12-29	Field derived strain	Total-RNA, polyA enriched	Whole non-blood fed adult females	(12)
Nakhonsawan, Thailand<br>BioProject accession: PRJEB15531	Collected during 2014-2015<br>G2 used for sequencing<br>Deposited in SRA: 2016-12-29	Field derived strain	Total-RNA, polyA enriched	Whole non-blood fed adult females	(12)
Nakhon Nayok Province, Thailand<br>BioProject accession: PRJNA276135	Wild collected 2008<br>Deposited in SRA: 2015-02-23	Wild pools	Total-RNA PolyA depleted, Ribosomal-RNA depleted	Whole mosquitoes	(24)
Thep Na Korn (KPP), Thailand<br>BioProject accession: PRJNA386455	Wild-caught in 2013<br>Sequencing conducted within ten generations<br>Deposited in SRA: 2017-05-12	Field derived strain	Total-RNA, polyA enriched	Midguts	(25)
Thep Na Korn (KPP), Thailand<br>BioProject accession: PRJNA540058	Wild-caught in 2013<br>Collected: 2018<br>Deposited in SRA: 2019-04-27	Field derived strain	ViralRNA<br>RNA extracted using QIAamp Viral RNA Mini Kit (Qiagen)<br>Library preparation using Nextera XT DNA Kit (Illumina)	Mosquito homogenates passaged on c6/36 cells and single mosquitoes used for sequencing.	(26)
Rayong (T32), Thailand<br>BioProject accession: PRJNA233429	Wild-caught: January 2009<br>Sequenced: 2010-2011, G_4<br>Deposited in SRA: 2014-01-04	Field derived strain	mRNA, polyA enriched<br>Illumina TruSeq RNA Sample Prep Kit v. 2 and mRNA Sequencing Sample Prep Kit.	Antenna	(2)
Ang Mo Kio, Singapore<br>Kindly made available to us via Milly Choy, October Sessions, and Ooi Eng Eong.	Established in 2010	Field derived strain	mRNA	Total mosquitoes	(27)
Ang Mo Kio, Singapore<br>BioProject accession: PRJNA533031	Colony established in 2010<br>Samples collected: 2016-12-31<br>Deposited in SRA: 2019-04-16	Lab strain	rRNA removal	Salivary glands from adult Female mosquitoes	(28)
**American Samples**					
Humaita, Tubiacanga and Belford Roxo, Rio de Janeiro, Brazil<br>BioProject accession: PRJNA277466	Wild-caught 2013<br>Deposited in SRA: 2015-02-13	Field derived strain	**Poly-A enriched and Ribosomal-RNA depleted**<br>TruSeq Stranded Total RNA kit<br>**sRNA sequencing**	Whole pools of mosquitoes	(29)
Humaita, Tubiacanga and Belford Roxo, Rio de Janeiro, Brazil<br>BioProject accession: PRJNA401335	Wild-caught 2013<br>F1 and F2 generations<br>Deposited in SRA: 2015-02-13	Field derived strain	**sRNA sequencing**<br>TruSeq Small RNA Library Prep Kit	Whole-body, Midgut and Carcass	(30)
Nova Iguaçu<br>Rio de Janeiro, Brazil<br>BioProject accession: PRJNA327841	Collected: Unknown<br>Deposited in SRA: 2016-07-05	Field derived strain	**sRNA sequencing**<br>TruSeq Small RNA Sample Prep Kit 7A (Illumina, USA)	Whole adult females	(31)
St-Georges & Cayenne, French Guiana<br>BioProject accession: PRJEB15531	Collected during 2014-2015<br>G2 used for sequencing<br>Deposited in SRA: 2016-12-29	Field derived strain	**mRNA -stranded**<br>TruSeq Stranded mRNA sample Prep kit	Whole non-blood fed adult females	(12)
Les Abymes & Petit-Bourg Guadeloupe<br>BioProject accession: PRJNA515586	Collected Les Abymes (2016, 2017), Petit-Bourg (2017)	Wild-caught	Viral RNA and DNA enrichment using published NetoVIR protocol	Multiple pools of adult male and female mosquitoes	(32)
Chetumal (CTM), Mexico<br>BioProject accession: PRJNA192626, PRJNA174376, PRJNA147583	CTM derived from the Yucatan Peninsula in Mexico, 2005, Sequenced at different times: during 2011-2012	Lab strain	**polyA enriched mRNA**<br>cDNA prepared with random primers	Whole blood-fed and sugar-fed female mosquitoes	(33-35)
Chetumal (CTM), Mexico<br>BioProject accession: PRJNA533980	Lab adapted CTM colony<br>Collection date: 2015<br>Deposited in SRA: 2019-04-21	Lab strain	**sRNA sequencing**<br>TruSeq Small RNA Sample Prep Kit (Illumina, San Diego CA)	Midgut tissues	(36)
Chiapas, Mexico<br>BioProject accession: PRJNA505498	Wild-caught August 2016	Wild-caught	**RNAse H/RT rRNA depletion**	Whole male and female mosquitoes	(37)
Cuernavaca, Mexico<br>BioProject accession: PRJNA301038	Caught in Morelos 2004<br>Samples collected: Apr-2013<br>Deposited in SRA: 2015-11-03	Lab strain	**Total-RNA, mRNA**<br>TruSeq RNA Sample Preparation Kit	Midgut tissue	(38)
Poza Rica, Mexico<br>BioProject accession: PRJNA645199	Collected in 2011<br>Black Lab at Colorado State University<br>Deposited in SRA: 2020-07-09	Lab strain	**Total-RNA random amplification**<br>KAPA HyperPrep kit	Total mosquitoes	(39)
Tapachula, Mexico<br>BioProject accession: PRJNA615972	Collected Jan-2016<br>F5 generation used for RNA-Seq	Field derived strain	**PolyA mRNA enriched**<br>KAPA Stranded mRNA-Seq Kit for Illumina.	Midgut of mosquitoes	(40)
Puerto Rico, (wild pools)*<br>BioProject accession: PRJNA307706	Wild-caught June 2014	Wild-caught	TruSeq Stranded Total RNA	Whole mosquitoes	(41)
Curepe, Trinidad<br>BioProject accession: PRJNA476553	Wild-caught November 2015<br>Deposited in SRA: 2018-06-18	Field derived strain	Total-RNA, Poly-A enriched	Whole female mosquitoes	(42)
Curepe, Trinidad<br>BioProject accession: PRJNA396016	Wild-caught November 2015<br>Deposited in SRA: Jul 27, 2017	Field derived strain	**sRNA sequencing**<br>Epicentre ScriptMiner kit (Cat. No. SMMP101212)	Second, third, fourth instar larvae and adults	(3)
Galveston, Texas, USA<br>BioProject accession: PRJNA382162, PRJNA399504	Wild-caught in Summer, 2003<br>Deposited in SRA: 2017-04-07	Lab strain	Total-RNA, PolyA enriched; sRNA	Whole mosquitoes	(43, 44)
Rexville D (REX-D) Red-eye strain, Puerto Rico<br>BioProject accession: PRJNA401335	Wild-caught: 2013<br>Deposited in SRA: 2017-09-04	Lab strain	**sRNA sequencing**<br>TruSeq Small RNA Library Prep Kit	Whole-body, Midgut and Carcass	(30)
Miami, USA (Lab adapted)<br>BioProject accession: PRJNA386859	Wild-caught 2016<br>Sequenced: 2017<br>Deposited in SRA: 2017-10-16	Field derived strain	Total-RNA, Ribosomal-RNA depleted, Strand-specific RNA-Seq & sRNA	Soma and germline tissues	(45)
Miami, USA<br>BioProject accession: PRJNA344504	Caught between August-Oct 2016<br>Deposited in SRA: 2017-05-17	Wild pools	Total-RNA, Ribosomal-RNA depleted,	Whole mosquitoes	(46)

Orlando, Florida, USA<br>BioProject accession: PRJEB15531	Collected in Orlando, Florida/~1940s,<br>Deposited in SRA: 2016-12-29	Lab strain	TruSeq Stranded mRNA sample Prep kit	Whole non-blood fed adult females	(12)
Orlando, Florida, USA<br>BioProject accession: PRJNA487057	Collected in Orlando, Florida/~1940s<br>Deposited in SRA: Aug 21, 2018	Lab strain	Total RNA, mRNA enriched<br>TruSeq DNA Library Preparation Kit	Whole adult female mosquitoes	(47)
Orlando, Florida, USA<br>BioProject accession: PRJNA148539	Collected in Orlando, Florida/~1940s,<br>Submitted to GEO: Jul 26, 2011	Lab strain & transgenic transformed lines	sRNA sequencing	Whole adult mosquitoes	(48)
Key West, Florida, USA<br>BioProject accession: PRJNA487057	Wild-caught in Key West (24.55° N, 81.78° W), and reared in the laboratory since 2011<br>Deposited in SRA: Aug 21, 2018	Lab strain	Total RNA, mRNA enriched<br>TruSeq DNA Library Preparation Kit	Whole adult female mosquitoes	(47)
Manatee County, USA<br>BioProject accession: PRJNA547758	Wild-caught 2016-2017 (May 15, 2016, and 33 June 19, 2017) four Manatee County sites	Wild-caught mosquitoes	Inverse-rRNA sequencing	Pooled abdomens from 20 adult females	(49)
**Unknown or Ambiguous**					
Rexville D (REX-D) Red-eye strain, Puerto Rico<br>BioProject accession: PRJNA192626	Deposited in SRA: 2013-03-07	Field derived strain	**mRNA sequencing**	RNA was extracted from pools of 3 female mosquitoes using the standard Trizol (Invitrogen) protocol.	
Rexville D (RexD), Puerto Rico<br>BioProject accession: PRJNA192626	Wild-caught 1988-1989<br>Sequencing: 2011<br>Deposited in SRA: 2013-03-07	Lab strain	**mRNA polyA enriched**<br>oligo-d(T)25 magnetic beads (Dynabeads: Invitrogen, CA, USA)<br>cDNA prepared with random primers	Whole blood-fed and sugar-fed female mosquitoes	(35)
Rexville D (RexD), Puerto Rico<br>BioProject accession: PRJNA396016	Wild-caught 1988-1989<br>Deposited in SRA: Jul 27, 2017	Lab strain	**sRNA sequencing**<br>Epicentre ScriptMiner kit (Cat. No. SMMP101212)	Second, third, fourth instar larvae and adults	(3)
Rexville D (RexD) Puerto Rico<br>BioProject accession: PRJNA206074	Wild-caught 1988-1989<br>Deposited in SRA: 2013-05-31	Lab strain	**sRNA sequencing**<br>FLASHPAGE system Library prep/ Small RNA Expression kit (Applied Biosystem)	Whole mosquitoes	(50, 51)
Higgs White Eye (HWE) strain (Variant of Rex-D), Puerto Rico<br>GEO accession: GSE95378<br>BioProject accession: PRJNA376828	Wild-caught 1988-1989<br>Deposited in GEO: Feb 26, 2017	Lab strain	Total-RNA, Poly-A enriched	Adult midgut tissue	(52)
Higgs White Eye (HWE) strain (Variant of Rex-D), Puerto Rico<br>BioProject accession: PRJNA475410	Deposited in SRA: 2018-06-10	Lab strain, transgenic lab strain	**sRNA sequencing**	Wild type, transgenic non-blood fed and blood-fed midguts	(53)
Rockefeller strain (ROCK)<br>BioProject accession: PRJNA396016	Caribbean origin caught ~1930,<br>Deposited in SRA: 2017-07-27	Lab strain	**sRNA sequencing**<br>Epicentre ScriptMiner kit (Cat. No. SMMP101212)	Second, third, fourth instar larvae and adults	(3)
Rockefeller strain<br>BioProject accession: PRJNA525617	Colony location: Wageningen, Netherlands<br>Collected: May-2019	Lab strain	sRNA	Whole mosquito body, without legs and wings	(54)
Rockefeller strain (ROCK)<br>BioProject accession: PRJNA388655	Caribbean origin caught ~1930,<br>Deposited in SRA: 2017-05-31	Lab strain	sRNA	Corpora allata-corpora etagen complexes	(55)
Rockefeller strain (ROCK)<br>BioProject accession: PRJNA311123	Caribbean origin caught ~1930,<br>Deposited in SRA: 2016-12-08	Lab strain	Total-RNA	Saliva	(56)
Rockefeller strain, (ROCK)<br>BioProject accession: PRJNA379149	Caribbean origin caught ~1930,<br>Data deposited to GEO: March 14, 2017	Lab strain	Total-RNA	Adult female midguts	(57)
Rockefeller strain (ROCK)<br>(available as MRA-734)<br>BioProject accession: PRJNA54037	Caribbean origin caught ~1930,<br>Sequencing conducted in October 2009<br>Deposited in SRA: 2010-10-15	Lab strain	Poly-A	Five female fat bodies pre and post blood meal	(58)
Rockefeller strain (ROCK)<br>BioProject accession: PRJNA246607	Caribbean origin caught ~1930,<br>Deposited in SRA: 2016-06-23	Lab strain	Total-RNA	Adult and larval malpighian tubules	(59)
Rockefeller (ROCK) (Publication)<br>Liverpool (Metadata)<br>BioProject accession: PRJNA314871	Deposited in SRA: 2016-03-10	Lab strain	Unknown	Adult female midguts	(60)
Rockefeller (ROCK)<br>BioProject accession: PRJNA507369	Caribbean origin caught ~1930,<br>Samples collected: 2017-06<br>Deposited in SRA: 2018-11-29	Lab strain	Poly-A	Male and female antennae	(61)
Rockefeller<br>BioProject accession: PRJNA551490	Deposited in SRA: 2019-06-27	Lab strain	Poly-A enriched stranded mRNA library	Head and thorax of adult female mosquitoes	(62)
UGAL mGFP (transgenic strain)<br>BioProject accession: PRJNA79693	Deposited in SRA: 2010-07-02	Transgenic mosquito	Poly-A enriched	Male and female first instar larvae	(63)
UGAL/Rockefeller<br>BioProject accession: PRJNA360643	Caribbean origin caught ~1930,<br>Deposited in SRA: 2017-01-09	Lab strain	CLIP-Seq bound to AGO-1, sRNA	Fat body	(64)
UGAL/Rockefeller<br>BioProject accession: PRJNA340082	Caribbean origin caught ~1930,<br>Collection date: May-2015<br>Deposited in SRA: 2016-08-25	Lab strain	Total-RNA	Larvae	(65)
UGAL, New Mexico State University<br>BioProject accession: PRJNA246243	Caribbean origin caught ~1930,<br>Deposited in SRA: 2014-05-05	Lab strain	Total-RNA, mRNA seq	Female fat body tissue	(66)
UGA<br>BioProject accession: PRJNA421500	Caribbean origin caught ~1930,<br>Sample collection date: Jun-2017<br>Deposited in SRA: 2017-12-07	Lab strain	Total-RNA, mRNA seq	Cultures containing first instars, gnotobiotic first instars	(67)
Liverpool, kh^w (white eye)<br>BioProject accession: PRJNA214946	Deposited in GEO: 2013-08-12	Lab strain	sRNA	Head and whole mosquito samples	(68)
Liverpool strain<br>BioProject accession: PRJNA147359	Deposited in GEO: Sep 20, 2011	Lab strain	sRNA	Whole female mosquitoes	(69)
Liverpool strain<br>BioProject accession: PRJEB15531	Collected during 2014-2015<br>Deposited in SRA: 2016-12-29	Field derived strain	**mRNA -stranded**<br>TruSeq Stranded mRNA sample Prep kit	Whole non-blood fed adult females	(12)
Liverpool strain<br>GEO Accession: GSE35161<br>BioProject accession: PRJNA150789	Deposited in GEO: Jan 17, 2012	Lab strain	sRNA	Whole female mosquitoes	(70)
Liverpool strain<br>BioProject accession: PRJNA232374	Deposited in SA: 2013-12-20	Lab strain	sRNA	Various developmental stages	(71)
Liverpool strain (Tu strain)<br>GEO Accession: GSE34480<br>BioProject accession: PRJNA151475	Deposited in GEO: Dec 15, 2011	Lab strain	mRNA	Various tissues	(72)

Liverpool strain<br>BioProject accession: PRJNA319716	Deposited in SRA: 2016-04-26	Lab strain	sRNA	Whole bodies	(73)
Liverpool strain<br>Colorado State University<br>BioProject accession: PRJNA261799	October 2009<br>Deposited in SRA: 2014-09-23	Lab strain	mRNA	Whole female and male mosquitoes	(74)
Liverpool strain<br>BioProject accession: PRJNA356717	Deposited in SRA: 2016-12-08	Lab strain	Total-RNA	Whole mosquitoes	(75)
Liverpool strain<br>BioProject accession: PRJNA298592	Deposited in SRA: 2015-10-13	Lab strain	Total-RNA, mRNA	Midguts	(76)
Liverpool strain<br>BioProject accession: PRJNA445828	Deposited in SRA: 2018-03-27	Lab strain	mRNA, Total-RNA	Fat body culture	(77)
Liverpool strain<br>BioProject accession: PRJNA192626	Sequencing: 2011<br>Deposited in SRA: 2013-03-07	Lab strain	cDNA prepared with random primers	Whole blood-fed and sugar-fed female mosquitoes	(35)
Liverpool strain<br>Pasteur Institute (Paris, France)<br>BioProject accession: PRJEB23850	Deposited to SRA: 2018-05-03	Lab strain	TruSeq Stranded mRNA sample Prep kit	Larval and adult stages	(78)
Liverpool strain<br>BioProject accession: PRJNA132131	Deposited in SRA: Oct 21, 2010	Lab strain	Total-RNA	Male and female salivary glands	(79)
Liverpool strain<br>GEO accession: GSE24872<br>BioProject accession: PRJNA132131	Deposited in GEO: 2010-10-21	Lab strain	Total-RNA	Whole female mosquitoes kept on sugar-fed diet versus whole mosquitoes blood-fed	(80)
Liverpool (Black-eyed strain)<br>GEO Accession: GSE53664<br>BioProject accession: PRJNA232599	Deposited in SRA: 2013-12-26	Lab strain	Total-RNA	Filarial worm infected thorax of female adult mosquitoes	(81)
Liverpool strain<br>BioProject accession: PRJNA255467	Deposited in SRA: 2014-07-30	Lab strain	Total-RNA	Female whole mosquitoes	(82)
Liverpool strain LVP_ib12<br>BioProject accession: PRJNA209388, PRJNA419241	MR4 BEI 735<br>Deposited in SRA: 2013-06-27	Lab strain	Total-RNA	Various developmental stages	(83)
Liverpool strain (LVPIB12)<br>BioProject accession: PRJNA236239	BEI Resources/CDC/MR-4<br>Collection date: Jan-2013<br>Deposited in SRA: 2014-02-05	Lab strain	Total-RNA	Major peripheral male and female sensory tissues, the brain and (female) ovary.	(84)
Liverpool (Black-eyed strain)<br>BioProject accession: PRJNA503353, PRJNA294263, PRJNA294264, PRJNA294265 and PRJNA294281.	Before 2018<br>Deposited in SRA: 2018-11-01	Lab strain	Total-RNA,<br>Ribosomal-RNA cleared	Whole adult body	(85)
Liverpool strain (LVP-IB12)<br>BioProject accession: SRP001531	Deposited in SRA: 2009-12-15	Lab strain	Poly-A enriched	Eggs and non-blood fed male and female adults	(86)
Liverpool strain (Strain LVP-IB12, Eggs, MRA-735)<br>BioProject accession: PRJNA595990	Deposited in SRA: 2019-12-16	Lab strain	**mRNA sequencing**<br>TruSeq Stranded mRNA Library Prep Kit	Malpighian tubules were isolated at day 1, day 2, and day 3 following infection by D. immitis and from sugar-fed controls	(87)
Liverpool (Black-eyed strain)<br>NR-48921<br>BioProject accession: PRJNA595990	Deposited in SRA: 2019-12-16	Lab strain	**mRNA sequencing**<br>TruSeq Stranded mRNA Library Prep Kit	Malpighian tubules were isolated at day 1, day 2, and day 3 following infection by D. immitis and from sugar-fed controls	(87)
Liverpool, kh^{w} (white eye)<br>BioProject accession: PRJNA261751	Deposited in SRA: 2014-09-22	Reference strain	**mRNA sequencing**	Adult carcass of whole male and female mosquitoes	(88)
Bayer laboratory colony<br>(Likely Liverpool Strain)<br>BioProject accession: PRJNA383480	Unknown<br>Before 2017<br>Deposited in SRA: 2017-04-19	Lab strain	sRNA	Whole female body	(89)

* Described as wild pools of unidentified mosquitoes, we verified that *Ae. aegypti* were in these mixed samples using COX1 BLASTn similarity.

Table S2. All *Ae. albopictus* samples analysed in this study and associated publications

Colony designation, City and country of original catch	Date of original catch/ Date and generation (g) of sequencing	Wild-caught/ Field derived strain/ Reference strain	Total-RNA or sRNA sequencing	Tissues sequenced	REF
**American samples**					
Chiapas, Mexico<br>BioProject accession: PRJNA505498	Wild-caught August 2016	Wild-caught	RNAse H/RT rRNA depletion	Female and Male whole	(37)
Gainesville (MRA-804), USA<br>BioProject accession: PRJNA327857	Unknown sequencing<br>Deposited in SRA: 2016-07-05	Field derived strain	Total-RNA	Female midgut and Carcass	(90)
Gainesville (MRA-804), USA<br>BioProject accession: PRJNA232489, PRJNA279095	RNA-Sequencing: September 2012<br>Deposited in SRA: 2015-03-22	Field derived strain	Total-RNA	Malpighian tubules	(91, 92)
Manassas, USA<br>BioProject accession: PRJNA268379	Manassas, VA, in 2008:<br>Sequencing conducted on F3 laboratory generation 03-Mar-2013<br>Deposited in SRA: 2014-11-25	Field derived strain	Total RNA	Whole adult mosquitoes	(93)
Manassas, USA<br>BioProject accession: PRJNA71721	Manassas, VA, in 2008<br>Sequencing conducted on F6 generation June 2010<br>Deposited in SRA: 2011-11-01	Field derived strain	Total RNA	400 female oocysts	(94)
Manassas, USA<br>BioProject accession: PRJNA158021	Manassas, VA, in 2008<br>F9 generation 2010<br>Deposited in SRA: 2012-04-06	Field derived strain	mRNA	Embryos under different conditions	(95)
Manassas, USA<br>BioProject accession: PRJNA187045	Manassas, VA, in 2008<br>Deposited in SRA: 2012-12-31	Field derived strain	mRNA	Pharate larvae, collected at 11, 21- and 40-days post-oviposition	(96)
Manassas, USA<br>BioProject accession: PRJNA360961	Manassas, VA, in 2008<br>F7 and F12 generation (2010)<br>Deposited in SRA: 2017-01-11	Field derived strain	sRNA	Pharate Larvae	(97)
Manassas, USA<br>BioProject accession: PRJNA694122<br>SRA accessions: SRR13521590, SRR13521539	Manassas, VA, in 2008<br>Deposited in SRA: 2021-01-22	Field derived strain	Total-RNA	Adult males used for sequencing M07 and M06	(98)
New Jersey, USA<br>BioProject accession: PRJNA223166	Unknown origin<br>Deposited in SRA: 2013-10-18	Field derived strain	Total RNA	Female and male reproductive organs	(99)

San Gabriel Valley, CA, USA<br>BioProject accession: PRJNA563095	F_{10} generation used for RNA-Seq experiments<br>Deposited in SRA: 2019-10-16	Field derived strain	mRNA, poly-A enriched	All developmental stages from male and female	(100)
San Gabriel Valley, CA, USA<br>BioProject accession: PRJNA610833	Described as (100)<br>Unknown generation used for sequencing<br>Deposited in SRA: 2020-03-06	Field derived strain	sRNA sequencing	Multiple developmental stages used.	(101)
Wise County, Virginia, USA<br>BioProject accession: PRJNA147359	Wise County, VA, in 2006<br>Deposited in GEO: Sep 20, 2011	Field derived strain	sRNA	Whole female mosquitoes and head and thorax.	(69)
Wise County, Virginia, United States of America<br>BioProject accession: PRJNA214946	Wise County, VA, in 2006<br>Deposited in SRA: April 2013	Laboratory strain	sRNA	Pools of 5 females	(68)
**Australian/ Asian Samples**					
Torres Strait Islands, Australia<br>BioProject accession: PRJNA450281	Unknown wild collection date.<br>Midgut samples collected in 2017<br>Deposited in SRA: 2018-04-15	Field derived strain	Total-RNA	Head and thorax & midgut of female adults	(102, 103)
Foshan strain, Guangdong, China<br>Housed at the University of Pavia<br>BioProject accession: PRJNA607026	Foshan strain, Guangdong, China and has been established in the laboratory since 1981<br>Collection date: 2017-07<br>Submission date: 2020-08-25	Foshan Reference strain	sRNA	Adult female carcasses and ovary pools	(104, 105)
Foshan strain, Guangdong, China<br>BioProject accession: PRJNA577831	Foshan, Guangdong, China and has been established in the laboratory since 1981<br>Deposited in SRA: 2019-10-16	Foshan Reference strain	mRNA enrichment<br>Oligo (dT) beads	30 female adults per sample	(106)
Foshan strain, Guangdong, China<br>BioProject accession: PRJNA178303	CDC Guangdong, China strain<br>Wild-caught in 1981,<br>Sequencing data deposited in SRA: 2012-10-26	Foshan Reference strain	sRNA	Multiple tissues and samples; Embryo, Larvae, Pupae, Adult male, Sugar-fed Adult female, Blood-fed adult female	(107)
Foshan strain, Guangdong, China<br>BioProject accession: PRJNA275727	CDC Guangdong, China strain<br>Wild-caught in 1981,<br>Deposited in SRA: 2015-02-21	Foshan Reference strain	Total-RNA	Multiple developmental stages: L1-L2 larvae, embryo and male transcriptome	(88)
Foshan strain, Guangdong, China<br>BioProject accession: PRJNA266286	established the laboratory in 1981<br>Deposited in SRA: 2014-11-04	Foshan Reference strain	Total-RNA, sRNA	Midgut sample data	(108)
Foshan strain, Guangdong, China<br>BioProject accession: PRJNA236401	CDC Guangdong, China strain<br>Wild-caught in 1981,<br>Deposited in SRA 2014-01-25	Foshan Reference strain	sRNA	Pooled midguts and whole mosquitoes	(109, 110)
Foshan strain, Guangdong, China<br>BioProject accession: PRJNA270274	CDC Guangdong, China strain<br>Wild-caught in 1981,<br>Deposited in SRA: 2015-03-06	Foshan Reference strain	Total-RNA	Multiple developmental stages: L1-L2 larvae, embryo and male and egg transcriptome	(111)
Foshan strain, Guangdong, China<br>BioProject accession: PRJNA495114	CDC Guangdong, China strain<br>Wild-caught in 1981,<br>Collection date: 15/03/2018	Foshan Reference strain	Total-RNA	Whole-body adult male and female mosquitoes	(112)
Longgang District, Shenzhen, China<br>BioProject accession: PRJNA475859	Sample collection September 2014,<br>Deposited in SRA: 2018-06-21	Field derived strain	Total-RNA	Individual mosquitoes	(113)
Yunnan, China<br>BioProject Accession: PRJNA472635<br>SRA accession: SRR7204305	August and September 2016<br>Deposited in SRA: 2018-05-22	Wild-caught (pools) individually identified	Ribosomal depleted Total-RNA	Whole mosquitoes, "mosquito_virome_3" library annotated as *Anopheles sinensis* but BLASTn and SRA taxonomy analysis revealed majority *Ae. albopictus*	(114)
Yunnan, China<br>BioProject Accession: PRJNA474574<br>SRA accession: SRR7286979	September and October 2017<br>Deposited in SRA: 2018-06-05	Wild-caught (pools) individually identified	Ribosomal depleted Total-RNA	Whole mosquitoes	(115)
Zhejiang, China<br>BioProject accession: PRJNA271540<br>SRA accession: SRR1745767	Collected 2013<br>Deposited in SRA: 2015-01-04	Wild-caught (pools)	Ribosomal depleted Total-RNA	Whole mosquitoes: Pool of 26 mosquitoes containing:<br>*Aedes albopictus, Armigeres subalbatus, Anopheles paraliae, Anopheles sinensis, Culex pipiens, Culex sp, Culex tritaeniorhynchus*	(116)
Kawasaki, Kanagawa Prefecture Japan<br>BioProject accession: PRJNA587399<br>SRA accession: SRR11213089, SRR11213090	Wild collected in 2008<br>45–50 generations of laboratory colonization used<br>Collected: 2015-01<br>Deposited in SRA: 2020-02-29	Field derived strain	sRNA	Three pools of 20 ovaries, testes or carcasses from the ISV-infected mosquitoes were combined for the generation of small RNA libraries.	(117)
Kuala Lumpur, Malaysia<br>BioProject accession: PRJNA694122	F_0 TROP males<br>Deposited in SRA: 2021-01-22	Field derived strain	Total-RNA	Pooled male (n=9) adult mosquitos	(98)
Phu Hoa, Ben Cat District,<br>Binh Duong Province Vietnam<br>BioProject accession: PRJNA293817	Collected in 2011<br>Sequencing conducted within 12 generations<br>Deposited in SRA: 2015-08-25	Field derived strain	sRNA	Pools of five whole mosquitoes	(118)
Phu Hoa, Ben Cat District,<br>Binh Duong Province Vietnam<br>BioProject accession: PRJNA587399	Collected: 2011-01-01<br>Experiments were performed within 16–20 generations<br>Deposited in SRA: 2019-11-04	Field derived strain	sRNA	Three pools of 20 ovaries, testes or carcasses from the ISV-infected mosquitoes were combined for the generation of small RNA libraries.	(117)
**European**					
Nice, France<br>BioProject accession: PRJNA449278	Mosquitoes collected in August 2011. F3, F11 used for mosquito infections<br>Deposited in SRA: 2018-04-09	Field derived strain	Total RNA<br>Nextera XT library preparation	Oral DENV infection followed by viral amplifications of collected saliva on C6/36 cells	(119)
Athens, Greece<br>BioProject accession: PRJNA282718	collected in Athens (Greece) in 2010;<br>Deposited in SRA: 2015-04-30	Field derived strain	Poly-A	Five to ten larvae in the late third to the early fourth stage were used for RNA extraction	(120)
Rome, Italy<br>BioProject accession: PRJNA314492	Caught in 2012,<br>Collection date: Oct-2012<br>Deposited in SRA: 2016-03-07	Field derived strain	Total RNA	Female antennae, Female palps, Female bodies, Male heads	(121)
Rimni, Italy<br>BioProject accession: PRJNA493544	Established in 2004<br>Collection date for RNA-Seq: 16-Nov-2017<br>Deposited in SRA: 2018-09-27	Field derived strain	Poly-A	RNA was extracted from both the heads and the fat bodies of the pooled mosquitoes	(122)
Ticino, Muzzano Switzerland<br>BioProject accession: PRJNA638077	Collected: August and September 2019<br>Deposited in SRA: 2020-06-08	Wild-caught	Total RNA	Pool of 40 mosquitoes	(123)

Table S3. Incidence of ISVs in *Aedes aegypti* colonies and wild-caught samples

Colony designation, City and country of original catch and Accession number	AeAV	FoV	PCLV	GMQLV-1	CFAV	HTV	LTLV	RaV	AeBNV-1	ReV	APLV-1	VeV	ATLV-1	ATV	CYV	AOLV-1	TMTLV	REF
**African**																		
Bakoumba, Gabon<br>BioProject accession: PRJNA386453	+	-	-	-	-	-	-	-	-	-	-	-	+	-	-	-	-	(1)
Rabai, Kenya (K2)<br>BioProject accession: PRJNA233429	+	-	+	-	-	+	-	+	-	-	+	-	-	+	-	-	-	(2)
Rabai, Kenya (K4)<br>BioProject accession: PRJNA233429	-	-	+	-	-	+	-	+	-	-	+	-	-	+	-	-	-	(2)
Rabai, Kenya (K14)<br>BioProject accession: PRJNA233429	+	-	+	-	-	+	-	-	-	-	+	-	-	-	-	-	-	(2)
Rabai, Kenya (K18)<br>BioProject accession: PRJNA233429	-	-	-	-	-	-	-	-	-	-	-	-	-	-	-	-	-	(2)
Rabai, Kenya (K19)<br>BioProject accession: PRJNA233429	-	-	+	-	-	-	-	-	-	-	-	+	-	+	-	-	-	(2)
Rabai, Kenya (K27)<br>BioProject accession: PRJNA233429	-	-	-	-	-	-	-	-	-	-	-	-	-	-	-	-	-	(2)
Rabai, Kenya K27 x K14 cross colonies (GP, GP2, HP1, HP2)<br>BioProject accession: PRJNA233429	+	-	+	-	-	+	-	-	-	-	+	-	-	-	-	-	-	(2)
Shauri Moyo (Moyo-S/R), Mombasa, Kenya<br>BioProject accession: PRJNA396016	-	-	-	-	-	-	-	-	-	-	-	-	-	-	-	-	-	(3)
Bundibugyo (U30), Uganda<br>BioProject accession: PRJNA233429	-	+	+	-	-	-	-	-	-	-	-	-	-	+	-	-	-	(2)
Wakiso, Uganda<br>BioProject accession: PRJNA556544	-	-	-	-	+	-	-	-	-	-	-	-	-	-	-	-	-	(4)
**Australian/ Asian Samples**																		
Cairns, Queensland, Australia<br>BioProject accession: PRJNA413709	-	-	+	+	+	+	-	-	-	-	+	-	+	+	-	+	-	(5)
Cairns, Queensland, Australia<br>Available on FigShare:<br>https://doi.org/10.26180/5bb0d5ae69035	-	-	+	-	+	+	-	-	-	-	+	-	+	+	-	+	-	(6)
Innisfail, North Queensland, Australia<br>BioProject accession: PRJNA630779	-	-	+	+	-	+	-	-	-	-	+	-	+	+	-	+	-	(8)
Innisfail, Australia<br>BioProject accession: PRJNA545086	-	-	+	+	-	+	-	-	-	-	+	-	+	+	-	+	-	(9)
North Queensland Strain, Australia<br>GEO accession: PRJNA255495	-	-	+	+	+	+	-	-	-	-	+	-	+	+	-	+	-	(7)
Townsville, Queensland, Australia<br>GEO accession: GSE100917<br>BioProject accession: PRJNA393424	-	-	+	-	+	+	-	-	-	-	+	-	+	+	-	-	-	(10)
Kampong Cham, Cambodia<br>BioProject: PRJNA556544	-	-	+	-	+	+	-	-	-	-	-	-	-	+	-	-	-	(4)
Phnom Penh, Cambodia<br>BioProject: PRJNA556544<br>SRA accession: SRR9830742	-	-	+	-	+	+	-	-	-	-	-	-	-	+	-	-	-	(4)
Fuzhou, Fujian province, China<br>BioProject accession: PRJNA476690	-	-	-	-	-	-	-	-	-	-	-	-	-	-	-	-	-	(11)
Bora-Bora, French Polynesia<br>BioProject accession: PRJEB15531	+	-	-	-	+	-	-	-	-	-	-	-	+	-	-	-	-	(12)
Bora-Bora, French Polynesia<br>BioProject accession: PRJEB1859	+	-	-	-	+	-	-	-	-	-	-	-	+	-	-	-	-	(13, 14)

Bora-Bora, French Polynesia<br>BioProject accession: PRJEB32622	+	-	-	-	+	-	-	-	-	-	-	-	+	-	-	-	-	(15)
**Colony designation, City and country of original catch and Accession number**	**AeAV**	**FoV**	**PCLV**	**GMQLV-1**	**CFAV**	**HTV**	**LTLV**	**RaV**	**AeBNV-1**	**ReV**	**APLV-1**	**VeV**	**ATLV-1**	**ATV**	**CYV**	**AOLV-1**	**TMTLV**	**REF**
Toahotu, Tahiti, French Polynesia<br>BioProject accession: PRJNA382618	-	-	-	-	-	-	-	-	-	-	-	-	-	-	-	-	-	(16)
New Delhi, India	-	-	+	-	-	-	-	-	-	-	+	-	+	+	+	-	-	(17)
Xayaboury, Luang Prabang, Vientiane Capital, Saravane, Attapeu Laos<br>BioProject accession: PRJEB37991	-	-	-	-	+	-	-	-	-	-	+	-	-	-	-	-	-	(18)
Jinjang, Selangor, Malaysia<br>BioProject accession: PRJNA320356	+	-	-	-	-	-	-	-	-	-	-	-	+	-	-	-	-	(19)
Bangkok, Thailand<br>BioProject accession: PRJNA413709	+	-	+	+	+	+	-	-	+	+	-	+	+	+	-	-	-	(5)
Bangkok, Thailand<br>BioProject accession: PRJNA612326	+	-	-	-	-	-	-	-	+	-	-	-	+	-	-	-	-	(20)
Kamphaeng Phet, Thailand<br>BioProject accession: PRJNA310013	+	-	+	+	-	+	-	-	+	-	-	-	+	-	-	-	-	(21)
Kamphaeng Phet, Thailand<br>BioProject accession: PRJNA487089	+	-	+	+	+	+	-	-	-	-	+	+	+	-	-	-	-	(22)
Kamphaeng Phet Province, Thailand<br>BioProject accession: PRJNA482553	+	-	-	-	-	+	-	-	-	-	+	-	-	-	-	-	-	(23)
Nakhonsawan, Thailand<br>BioProject accession: PRJEB15531	-	-	+	-	-	+	-	-	+	+	-	-	+	-	-	-	-	(12)
Nakhon Nayok Province, Thailand<br>BioProject accession: PRJNA276135	+	-	+	-	-	+	-	-	-	+	-	+	+	+	-	-	-	(24)
Phetchaburi, Thailand<br>BioProject accession: PRJEB15531	-	-	+	+	+	+	-	-	+	-	-	-	+	-	-	-	-	(12)
Thep Na Korn (KPP), Thailand<br>BioProject accession: PRJNA386455	+	-	+	-	+	+	-	-	-	+	+	+	-	+	-	-	-	(25)
Thep Na Korn (KPP), Thailand<br>BioProject accession: PRJNA540058	-	-	-	-	+	-	-	-	-	-	-	-	-	-	+	-	-	(26)
Rayong (T32), Thailand<br>BioProject accession: PRJNA233429	+	-	-	+	-	+	-	-	-	-	-	-	-	+	-	-	-	(2)
Ang Mo Kio, Singapore	-	-	+	-	+	+	-	-	-	-	+	-	+	-	-	-	-	(27)
Ang Mo Kio, Singapore<br>BioProject accession: PRJNA533031	-	-	+	-	+	+	-	-	-	-	+	-	+	-	-	-	-	(28)
**American**																		
Tapachula, Mexico<br>BioProject accession: PRJNA615972	+	-	+	-	-	+	-	-	-	-	+	-	-	-	-	-	-	(40)
Humaita, Tubiacanga and Belford Roxo, Rio de Janeiro, Brazil<br>BioProject accession: PRJNA277466	-	-	+	-	-	+	-	-	-	-	-	-	-	-	-	-	-	(29)
Humaita, Tubiacanga and Belford Roxo, Rio de Janeiro, Brazil<br>BioProject accession: PRJNA401335	-	-	+	-	-	+	-	-	-	-	-	-	-	-	-	-	-	(30)
Nova Iguaçu, Rio de Janeiro, Brazil<br>BioProject accession: PRJNA327841	-	-	+	-	-	+	-	-	-	-	-	-	-	-	-	-	-	(31)
St-Georges & Cayenne, French Guiana<br>BioProject accession: PRJEB15531	+	-	+	-	+	+	-	-	+	+	-	-	+	+	-	-	-	(12)
Les Abymes & Petit-Bourg, Guadeloupe<br>BioProject accession: PRJNA515586	+	-	+	+	+	+	-	-	-	+	+	-	+	+	-	-	-	(32)

Colony designation, City and country of original catch and Accession number	AeAV	FoV	PCLV	GMQLV-1	CFAV	HTV	LTLV	RaV	AeBNV-1	ReV	APLV-1	VeV	ATLV-1	ATV	CYV	AOLV-1	TMTLV	REF
Chetumal (CTM), Mexico<br>BioProject accession: PRJNA192626	+	-	-	-	-	-	-	-	-	+	-	-	-	-	-	-	-	(33)
Chetumal (CTM), Mexico<br>BioProject accession: PRJNA174376	+	-	-	-	-	-	+	-	-	+	-	-	+	-	-	-	-	(34)
Chetumal (CTM), Mexico<br>BioProject accession: PRJNA147583	+	-	-	-	-	-	-	-	-	+	-	-	-	-	-	-	-	(35)
Chetumal (CTM), Mexico<br>BioProject accession: PRJNA533980	-	-	-	-	-	-	-	-	-	-	-	-	-	-	-	-	-	(36)
Chiapas, Mexico<br>BioProject accession: PRJNA505498	+	-	+	-	-	+	-	-	-	+	+	-	-	-	-	-	-	(37)
Cuernavaca, Mexico<br>BioProject accession: PRJNA301038	+	-	-	+	+	-	-	-	-	-	-	-	-	-	-	-	-	(38)
Poza Rica, Mexico<br>BioProject accession: PRJNA645199	+	-	-	-	-	-	+	-	-	+	-	+	-	-	-	-	-	(39)
Puerto Rico, (wild pools)*<br>BioProject accession: PRJNA307706	+	-	+	-	-	+	-	-	-	+	-	+	+	-	-	-	-	(41)
Curepe, Trinidad<br>BioProject accession: PRJNA476553	+	-	+	-	+	-	-	-	+	+	+	+	-	-	-	-	-	(42)
Curepe, Trinidad<br>BioProject accession: PRJNA396016	+	-	+	-	-	+	-	-	-	+	+	+	-	-	-	-	-	(3)
Galveston, Texas, USA<br>BioProject accession: PRJNA382162, PRJNA399504	-	-	-	-	+	-	-	-	-	-	+	-	-	-	-	-	-	(43, 44)
Miami, USA (Lab adapted)<br>BioProject accession: PRJNA386859	+	-	-	+	-	+	-	-	+	+	+	+	+	+	-	-	-	(45)
Miami, USA<br>BioProject accession: PRJNA344504	+	-	+	+	+	+	-	-	+	+	+	+	+	+	-	-	-	(46)
Orlando, Florida, USA<br>BioProject accession: PRJEB15531	-	-	-	-	-	-	-	-	-	-	-	-	-	-	-	-	-	(12)
Orlando, Florida, USA<br>BioProject accession: PRJNA487057	-	-	-	-	-	-	-	-	+	-	-	-	+	-	-	-	-	(47)
Orlando, Florida, USA<br>BioProject accession: PRJNA148539	-	-	-	-	-	-	-	-	-	-	-	-	-	-	-	-	-	(48)
Key West, Florida, USA<br>BioProject accession: PRJNA487057	-	-	-	-	-	-	-	-	+	-	-	-	+	-	-	-	-	(47)
Manatee County, USA<br>BioProject accession: PRJNA547758	+	-	+	+	+	+	-	-	+	+	+	-	+	-	-	-	-	(49)
**Reference Ambiguous or Unknown**																		
Rexville D (REX-D) Red-eye strain, Puerto Rico<br>BioProject accession: PRJNA401335	-	-	+	-	-	+	-	-	-	-	-	-	-	-	-	-	-	(30)
Rexville D (REX-D) Red-eye strain, Puerto Rico:<br>BioProject accession: PRJNA192626	-	-	-	-	-	-	-	-	-	-	-	-	-	-	-	-	-	(33)
Rexville D (RexD), Puerto Rico<br>BioProject accession: PRJNA192626	-	-	-	-	-	-	-	-	-	-	-	-	-	-	-	-	-	(35)
Rexville D (RexD), Puerto Rico<br>BioProject accession: PRJNA396016	-	-	-	-	-	-	-	-	-	-	-	-	-	-	-	-	-	(3)
Rexville D (RexD) Puerto Rico<br>BioProject accession: PRJNA206074	-	-	-	-	-	-	-	-	-	-	-	-	-	-	-	-	-	(50, 51)
Higgs White Eye (HWE) strain (Variant of Rex-D), Puerto Rico<br>GEO accession: GSE95378<br>BioProject accession: PRJNA376828	-	-	-	-	-	-	-	-	-	-	-	-	-	-	-	-	-	(52)

**Colony designation, City and country of original catch and Accession number**	**AeAV**	**FoV**	**PCLV**	**GMQLV-1**	**CFAV**	**HTV**	**LTLV**	**RaV**	**AeBNV-1**	**ReV**	**APLV-1**	**VeV**	**ATLV-1**	**ATV**	**CYV**	**AOLV-1**	**TMTLV**	**REF**
Higgs White Eye (HWE) strain (Variant of Rex-D), Puerto Rico<br>BioProject accession: PRJNA475410	-	-	-	-	-	-	+	-	-	-	-	-	-	-	-	-	-	(53)
Rockefeller strain (ROCK)<br>BioProject accession: PRJNA396016	-	-	-	-	-	-	-	-	-	-	-	-	-	-	-	-	-	(3)
Rockefeller strain<br>BioProject accession: PRJNA525617	-	-	-	-	-	-	-	-	-	-	-	-	-	-	-	-	-	(54)
Rockefeller strain (ROCK)<br>BioProject accession: PRJNA388655	-	-	-	-	-	-	-	-	-	-	-	-	-	-	-	-	-	(55)
Rockefeller strain (ROCK)<br>BioProject accession: PRJNA311123	-	-	-	-	-	-	-	-	-	-	-	-	-	-	-	-	-	(56)
Rockefeller strain, (ROCK)<br>BioProject accession: PRJNA379149	-	-	-	-	-	-	-	-	-	-	-	-	-	-	-	-	-	(57)
Rockefeller strain (ROCK)<br>BioProject accession: PRJNA54037	-	-	-	-	-	-	-	-	-	-	-	-	-	-	-	-	-	(58)
Rockefeller strain (ROCK)<br>BioProject accession: PRJNA246607	-	-	-	-	-	-	-	-	-	-	-	-	-	-	-	-	-	(59)
Rockefeller (ROCK) (Publication)<br>Liverpool (Metadata)<br>BioProject accession: PRJNA314871	-	-	-	-	-	-	-	-	-	-	-	-	+	-	-	-	-	(60)
Rockefeller (ROCK)<br>BioProject accession: PRJNA507369	-	-	-	-	-	-	-	-	-	-	-	-	-	-	-	-	+	(61)
Rockefeller (ROCK)<br>BioProject accession: PRJNA551490	-	-	-	-	-	-	-	-	-	-	-	-	-	-	-	-	-	(62)
UGAL mGFP (transgenic strain)<br>BioProject accession: PRJNA79693	-	-	-	-	-	-	-	-	-	-	-	-	-	-	-	-	-	(63)
UGAL/Rockefeller<br>BioProject accession: PRJNA360643	-	-	-	-	-	-	-	-	-	-	-	-	-	-	-	-	-	(64)
UGAL/Rockefeller<br>BioProject accession: PRJNA340082	-	-	-	-	-	-	-	-	-	-	-	-	-	-	-	-	-	(65)
UGAL New Mexico State University<br>BioProject accession: PRJNA246243	-	-	-	-	-	-	-	-	-	-	-	-	-	-	-	-	-	(66)
UGA<br>BioProject accession: PRJNA421500	-	-	-	-	-	-	-	-	-	-	-	-	-	-	-	-	-	(67)
UGAL<br>BioProject accession: PRJNA598930	-	-	-	-	-	-	-	-	-	-	-	-	-	-	-	-	-	(124)
Liverpool, kh^w (white eye)<br>BioProject accession: PRJNA261751	-	-	-	-	-	-	+	-	-	-	-	-	+	-	-	-	-	(88)
Liverpool, kh^w (white eye)<br>BioProject accession: PRJNA214946	-	-	-	-	-	-	-	-	-	-	-	-	+	-	-	-	-	(68)
Liverpool strain<br>BioProject accession: PRJNA147359	-	-	-	-	-	-	-	-	-	-	-	-	+	-	-	-	-	(69)
Liverpool strain<br>BioProject accession: PRJEB15531	-	-	-	-	+	-	-	-	-	+	-	-	+	-	-	-	-	(12)
Liverpool strain<br>GEO Accession: GSE35161<br>BioProject accession: PRJNA150789	-	-	-	-	-	-	+	-	-	-	-	-	+	-	-	-	-	(70)
Liverpool strain<br>BioProject accession: PRJNA232374	-	-	-	-	-	-	+	-	-	-	-	-	+	-	-	-	-	(71)
Liverpool strain (Tu strain)<br>GEO Accession: GSE34480<br>BioProject accession: PRJNA151475	-	-	-	-	-	-	+	-	-	-	-	-	+	-	-	-	-	(72)
Liverpool strain<br>BioProject accession: PRJNA319716	-	-	-	-	-	-	-	-	-	-	-	-	+	-	-	-	-	(73)
Liverpool strain Colorado State University<br>BioProject accession: PRJNA261799	-	-	-	-	-	-	+	-	-	-	-	-	+	-	-	-	-	(74)
Liverpool strain<br>BioProject accession: PRJNA356717	-	-	-	-	-	-	-	-	-	-	-	-	+	-	-	-	-	(75)

Colony designation, City and country of original catch and Accession number	AeAV	FoV	PCLV	GMQLV-1	CFAV	HTV	LTLV	RaV	AeBNV-1	ReV	APLV-1	VeV	ATLV-1	ATV	CYV	AOLV-1	TMTLV	REF
Liverpool strain<br>BioProject accession: PRJNA298592	-	-	-	-	-	-	-	-	+	-	-	-	+	-	-	-	-	(76)
Liverpool strain<br>BioProject accession: PRJNA445828	-	-	-	-	-	-	+	-	-	-	-	-	+	-	-	-	-	(77)
Liverpool strain<br>BioProject accession: PRJNA192626	-	-	-	-	-	-	-	-	-	-	-	-	-	-	-	-	-	(35)
Liverpool strain<br>Pasteur Institute (Paris, France)<br>BioProject accession: PRJEB23850	-	-	-	-	+	-	-	-	-	-	-	-	+	-	-	-	-	(78)
Liverpool strain<br>BioProject accession: PRJNA132131	-	-	-	-	-	-	-	-	+	-	-	-	-	-	-	-	-	(79)
Liverpool strain<br>GEO accession: GSE24872<br>BioProject accession: PRJNA132131	-	-	-	-	-	-	-	-	-	-	-	-	-	-	-	-	-	(80)
Liverpool (Black-eyed strain)<br>GEO Accession: GSE53664<br>BioProject accession: PRJNA232599	-	-	-	-	-	-	+	-	-	-	-	-	+	-	-	-	-	(81)
Liverpool strain<br>BioProject accession: PRJNA255467	-	-	-	-	-	-	-	-	-	-	-	-	+	-	-	-	-	(82)
Liverpool strain LVP_ib12<br>BioProject accession: PRJNA209388, PRJNA419241	-	-	-	-	-	-	-	-	-	-	-	-	-	-	-	-	-	(83)
Liverpool strain (LVPIB12)<br>BioProject accession: PRJNA236239	-	-	-	-	-	-	-	-	-	-	-	-	-	-	-	-	-	(84)
Liverpool (Black-eyed strain)<br>BioProject accession: PRJNA503353, PRJNA294263, PRJNA294264, PRJNA294265 and PRJNA294281.	-	-	-	-	-	-	-	-	-	-	-	-	+	-	-	-	-	(85)
Liverpool strain (LVP-IB12)<br>BioProject accession: SAMN00006233	-	-	-	-	-	-	-	-	-	-	-	-	-	-	-	-	-	(86)
Liverpool strain (Strain LVP-IB12, Eggs, MRA-735) Liverpool (Black-eyed strain) NR-48921<br>BioProject accession: PRJNA595990	-	-	-	-	-	-	-	-	-	-	-	-	+	-	-	-	-	(87)
Bayer laboratory colony (Likely Liverpool Strain)<br>BioProject accession: PRJNA383480	-	-	-	-	-	-	-	-	-	-	-	-	+	-	-	-	-	(89)

Legend: AeAV: Aedes aegypti anphevirus, **FoV:** Formosus virus, **GMQLV-1:** Guadeloupe mosquito quaranja-like virus 1, **PCLV:** Phasi Charoen Like virus, **CFAV:** Cell fusing agent virus, **HTV:** Humaita-Tubiacanga virus, **LTLV:** Liverpool tombus-like virus, **RaV:** Rabai virus, **AeBNV-1**: Aedes bi-negev like virus 1, **VeV:** Verdadero virus, **ATLV-1:** Aedes aegypti toti-like virus 1, **ATV:** Aedes aegypti toti virus, **CYV:** Culex Y virus, **AOLV-1:** Aedes orbi-like virus 1

Table S4. Incidence of ISVs in *Aedes albopictus* colonies and wild-caught samples

Colony designation, City and country of original catch	AaAV	SGM	LMCV	APV	AOMV-2	UsV	JMTV	AeFV	AaNLV	AeBNV-2	TMTLV	LTLV	FTBLV	WBLV-4	DANV	NeV	AalDNV-2	CpDNV	REF
**American samples**																			
Chiapas, Mexico<br>BioProject accession: PRJNA505498	-	-	-	-	-	-	-	-	-	-	-	-	-	+	-	-	-	-	(37)
Gainesville (MRA-804), USA<br>BioProject accession: PRJNA327857	-	-	-	-	-	-	-	-	-	-	+	-	-	-	-	-	-	-	(90)
Gainesville (MRA-804), USA<br>BioProject accession: PRJNA232489, PRJNA279095	+	-	-	-	-	-	-	-	-	-	+	-	-	-	-	-	-	-	(91, 92)
Manassas, USA<br>BioProject accession: PRJNA268379	+	-	-	+	-	-	-	-	-	+	+	-	-	+	-	+	-	-	(93)
Manassas, United States of America<br>BioProject accession: PRJNA71721	+	-	-	-	-	-	-	-	-	-	+	-	-	-	-	+	-	-	(94)
Manassas, USA<br>BioProject accession: PRJNA158021	+	-	-	+	-	-	-	-	-	-	+	-	-	+	-	-	+	-	(95)
Manassas, USA<br>BioProject accession: PRJNA187045	+	-	-	+	-	-	-	-	-	-	+	-	-	+	-	-	+	-	(96)
Manassas, USA<br>BioProject accession: PRJNA360961	+	-	-	+	-	-	-	-	-	-	+	-	-	+	-	-	-	-	(97)
Manassas, USA<br>BioProject accession: PRJNA694122	-	-	-	-	-	-	-	-	-	-	+	-	-	+	-	-	-	-	(98)
New Jersey, USA<br>BioProject accession: PRJNA223166	+	-	-	+	-	-	-	-	-	-	-	-	-	+	-	-	-	-	(99)
San Gabriel Valley, CA, USA<br>BioProject accession: PRJNA563095	+	+	-	+	-	+	-	-	-	-	-	-	-	-	-	+	-	-	(100)
San Gabriel Valley, CA, USA<br>BioProject accession: PRJNA610833	+	+	-	+	-	-	-	-	-	-	-	-	-	-	+	+	-	-	(101)
Wise County, Virginia, USA<br>BioProject accession: PRJNA147359	+	-	-	-	-	-	-	-	-	-	-	-	-	-	-	+	-	-	(69)
Wise County, Virginia, USA<br>BioProject accession: PRJNA214946	-	-	-	-	-	-	-	-	-	-	+	+	+	-	-	-	-	-	(68)
**Australian/ Asian Samples**																			
Torres Strait Islands, Australia<br>BioProject accession: PRJNA450281	-	-	-	-	+	-	-	-	-	-	-	-	-	-	-	-	-	-	(102, 103)
Foshan strain, Guangdong, China<br>Housed at the University of Pavia<br>BioProject accession:: PRJNA607026	-	-	-	-	-	-	-	-	-	-	+	-	-	-	-	+	-	-	(104, 105)
Foshan strain, Guangdong, China<br>BioProject accession: PRJNA577831	-	-	-	-	-	-	-	-	-	-	-	-	+	-	-	-	-	-	(106)
Foshan strain, Guangdong, China<br>BioProject accession: PRJNA178303	-	-	-	-	-	-	-	-	-	-	-	-	-	-	-	+	-	-	(107)
Foshan strain, Guangdong, China<br>BioProject accession: PRJNA275727	-	-	-	-	+	-	-	-	-	-	-	-	-	-	-	-	-	+	(88)
Foshan strain, Guangdong, China<br>BioProject accession: PRJNA266286	-	-	-	-	-	-	-	-	-	-	-	-	-	-	-	-	-	-	(108)
Foshan strain, Guangdong, China<br>BioProject accession: PRJNA236401	-	-	-	-	-	-	-	-	-	-	-	-	+	-	-	+	-	+	(109, 110)
Foshan strain, Guangdong, China<br>BioProject accession: PRJNA270274	-	-	-	-	+	-	-	-	-	-	-	-	-	+	-	-	-	+	(111)
Foshan strain, Guangdong, China<br>BioProject accession: PRJNA495114	-	-	-	-	-	-	-	-	-	-	+	-	+	-	-	-	-	+	(112)

Longgang District, Shenzhen, China<br>BioProject accession: PRJNA475859	+	-	+	+	-	-	-	-	+	+	-	-	-	+	-	-	-	-	(113)
**Colony designation, City and country of original catch**	**AaAV**	**SGM**	**LMCV**	**APV**	**AOMV-2**	**UsV**	**JMTV**	**AeFV**	**AaNLV**	**AeBNV-2**	**TMTLV**	**LTLV**	**FTBLV**	**WBLV -4**	**DANV**	**NeV**	**AalDNV -2**	**CpDNV**	**REF**
Yunnan, China<br>BioProject accession: PRJNA472635<br>SRA accession: SRR7204305	+	+	-	+	-	+	-	-	-	-	-	-	-	+	-	-	-	+	(114)
Yunnan, China<br>BioProject accession: PRJNA474574<br>SRA accession: SRR7286979	-	-	-	-	-	-	-	-	+	-	-	-	-	+	-	-	-	+	(115)
Zhejiang, China<br>BioProject accession: PRJNA271540<br>SRA accession: SRR1745767	+	-	-	+	+	+	-	-	+	-	-	-	-	+	-	+	-	+	(125)
Kawasaki, Kanagawa Prefecture, Japan<br>BioProject accession: PRJNA587399	+	+	-	-	-	-	-	-	-	-	+	-	-	+	+	-	-	-	(117)
Kuala Lumpur, Malaysia<br>BioProject accession: PRJNA694122	+	-	-	-	-	-	-	-	-	-	+	-	-	+	-	-	-	-	(98)
Phu Hoa, Binh Duong Province Vietnam<br>BioProject accession: PRJNA293817	-	-	-	+	-	-	-	-	-	-	+	-	-	+	+	-	-	-	(118)
Phu Hoa, Binh Duong Province Vietnam<br>BioProject accession: PRJNA587399	-	-	-	+	-	-	-	-	-	-	+	-	-	+	+	+	-	-	(117)
**European**																			
Nice, France<br>BioProject accession: PRJNA449278	-	-	-	-	-	-	-	+	-	-	+	-	-	-	-	-	-	-	(119)
Athens, Greece<br>BioProject accession: PRJNA282718	-	-	-	-	-	-	-	-	-	-	-	-	-	-	-	-	-	-	(120)
Rome, Italy<br>BioProject accession: PRJNA314492	+	-	-	+	+	-	-	-	-	-	-	-	-	+	-	-	-	-	(121)
Rimini, Italy<br>BioProject accession: PRJNA493544	-	-	-	-	-	-	+	-	-	-	-	-	-	-	-	-	-	-	(122)
Ticino, Muzzano Switzerland<br>SRA accession: PRJNA638077	+	+	-	+	+	+	-	-	-	-	-	-	-	+	-	+	-	-	(123)

Legend: SGM: San Gabriel Mononegavirus, **AaAV**: Aedes albopictus Anphevirus (126), **LMCV**: Longgang mosquito chu-like virus, **APV**: Aedes phasma-virus (127), **AOMV-2**: Aedes orthomyxo-like virus 2, **UsV:** Usinis virus, **JMTV:** Jingmen tick virus, **AeFV**: Aedes flavivirus, **AaNLV**: Aedes albopictus negev-like virus (128), **AeBNV-2**: Aedes bi-negev like virus 2, **TMTLV:** Tiger mosquito bi-segmented tombus-like virus, **LTLV:** Liverpool tombus-like virus, **FTBLV:** Foshan tombus-like virus, **WBLV-4:** Wenzhou sobemo-like virus 4, **DANV:** Drosophila American nodavirus, **AalDNV-2:** Aedes albopictus densovirus 2, **CpDNV:** Culex pipiens densovirus

Table S5. Incidence of ISVs in *Aedes aegypti* and *Aedes albopictus* cell lines

Cell lines	REF	AeAV	SGM	PCLV	AaNLV	CFAV	LTLV	FTLV	TMTLV	AalDNV-2	WSBLV-4	DANV	CYV	CppDNV
Ae. aegypti														
Aag2 PRJEB13078 Total-RNA	(129)	-	-	+	-	+	-	-	-	+	-	-	-	-
Ae. aegypti Aag2 PRJNA238976	(130, 131)	-	-	+	-	+	-	-	-	+	-	-	-	-
Aag2 PRJNA516662 sRNA	(132)	-	-	+	-	+	-	-	-	+	-	-	-	-
Aag2 PRJNA493127 sRNA	(133)	-	-	+	-	+	-	-	-	+	-	-	-	-
Aag2 PRJNA464394 Total-RNA	(134)	-	-	+	-	+	-	-	-	+	-	-	-	-
Aag2 Arthropod Cell Line Initiative (Broad Institute) mRNA		-	-	+	-	+	-	-	-	+	-	-	-	+
Aag2 PRJNA435568	(135)	-	-	+	-	+	-	-	-	+	-	-	-	-
Aag2 PRJNA396680 sRNA	(136)	-	-	+	-	+	-	-	-	+	-	-	-	-
Aag2 PRJNA525601 sRNA	(137)	-	-	+	-	+	-	-	-	+	-	-	-	-
Aag2 PRJNA482553 mRNA	(23)	-	-	+	-	+	-	-	-	+	-	-	+	-
Aag2 PRJEB15203 sRNA	(138)	-	-	+	-	+	-	-	-	+	-	-	+	-
CCL-125 PRJNA147359 sRNA	(69)	+	-	+	-	-	-	-	-	-	-	-	+	-
CCL-125 Arthropod Cell Line Initiative (Broad Institute) mRNA		+	-	-	-	-	-	-	-	-	-	-	-	+
CCL-125 PRJNA610833 sRNA	(101)	+	-	+	-	-	-	-	-	+	+	+	-	-
Ae. albopictus														
C7-10 PRJNA147359 sRNA	(69, 139)	-	-	-	-	-	-	-	-	-	+	-	-	-
C7-10 Arthropod Cell Line Initiative (Broad Institute) mRNA		-	-	-	-	-	-	-	-	+	+	-	-	-
C7-10 PRJNA610833 SRR11252295	(101)	-	-	+	-	-	-	-	-	-	-	+	-	-
C7-10 PRJNA147359 sRNA	(69)	-	-	-	-	-	-	-	-	-	+	-	-	-
U4.4 Arthropod Cell Line Initiative (Broad Institute) mRNA		-	-	-	-	-	-	-	-	-	-	-	-	+
U4.4 PRJNA464394 Total-RNA	(134)	-	-	-	-	-	-	-	-	-	-	-	+	-
U4.4 PRJNA610833 SRR11252301 sRNA	(101)	-	-	-	-	-	-	-	-	-	-	-	+	-
U4.4 PRJNA559096 sRNA	(140)	-	-	-	-	-	-	-	-	-	-	-	+	-
U4.4 PRJEB15203 sRNA	(138)	-	-	-	-	-	-	-	-	-	-	-	+	-
U4.4 SRP062828 sRNA	(118)	-	-	-	-	-	-	-	-	-	-	+	+	-

U4.4 PRJNA525601	(137)	-	-	+	-	-	-	-	-	-	-	-	+	-
**Cell lines**	**REF**	**AeAV**	**SGM**	**PCLV**	**AaNLV**	**CFAV**	**LTLV**	**FTLV**	**TMTLV**	**AalDNV-2**	**WSBLV-4**	**DANV**	**CYV**	**CppDNV**
C6/36 PRJNA464394 Total-RNA	(134)	-	-	-	-	-	-	-	-	-	-	-	-	-
C6/36 PRJNA436549 mRNA	(141)	-	-	-	-	-	-	-	-	-	-	-	-	-
C6/36 PRJNA610833: SRR11252294 sRNA	(101)	-	-	+	-	-	-	-	-	+	+	+	-	-
C6/36 PRJNA610833: SRR11252293 sRNA	(101)	-	-	+	-	-	-	-	-	+	-	-	-	-
C6/36 PRJNA556544 vRNA	(4)	-	-	-	-	-	-	-	-	+	-	+	-	-
C6/36 PRJNA703391 mRNA	(142)	-	-	-	-	-	-	-	-	-	-	-	-	-
C6/36 PRJNA516868 mRNA	(143)	-	-	-	-	-	-	-	-	-	-	-	-	-
C6/36 PRJNA503588 vRNA	(26)	-	-	-	-	-	-	-	+	+	-	+	-	-
C6/36 PRJNA540058 vRNA	(26)	-	-	-	-	-	-	-	+	+	-	+	-	-
C6/36 PRJNA565571 mRNA	(144)	-	-	-	-	-	-	-	-	-	-	-	+	-
C6/36 PRJNA449278 mRNA	(119)	-	-	-	-	-	-	-	+	+	-	+	-	-
C6/36 PRJNA430004 mRNA	(145)	-	-	-	-	-	-	-	-	-	-	+	-	-
C6/36 PRJNA345486 mRNA	(146)	-	-	-	-	-	-	-	-	-	-	-	-	-
C6/36 PRJNA525601	(137)	-	-	-	-	-	-	-	-	-	-	-	-	-
C6/36 PRJNA374578 sRNA	(147)	-	-	-	-	-	-	-	-	-	-	-	-	+
C6/36 SRP062828 sRNA	(118)	-	-	-	-	-	-	-	-	-	-	+	+	-
C6/36 PRJNA147359 sRNA	(69)	-	-	-	-	-	+	+	-	+	-	-	-	-
***Wolbachia* infected *Aedes* cells**														
Ae. albopictus wAlbB Aa23 PRJNA670506	(148)	+	+	-	+	-	-	-	-	+	+	-	-	-
Ae. albopictus RML-12.wMelPop PRJNA227164	(149)	+	+	+	-	-	-	-	-	-	+	-	-	-
Ae. albopictus C6/36.wMelPop PRJNA516868, PRJNA266744	(143, 150)	+	+	+	-	-	-	-	-	-	+	-	-	-
Ae. aegypti Aag2.wMelPop PRJNA238976	(130, 131)	+	+	+	-	-	-	-	-	+	+	-	-	-
Ae. aegypti wAlbB PRJNA670506	(148)	+	+	+	+	-	-	-	-	+	-	-	-	-

Legend: **AeAV:** Aedes aegypti anphevirus, **SGM:** San Gabriel Mononegavirus **PCLV:** Phasi Charoen Like virus, **AaNLV:** Aedes albopictus negev-like virus, **CFAV:** Cell fusing agent virus, **LTLV:** Liverpool tombus-like virus, **FTBLV:** Foshan tombus-like virus, **TMTLV:** Tiger mosquito bi-segmented tombus-like virus, **AalDNV-2**: Aedes albopictus densovirus, **WSBL-4:** Wenzhou-Sobemo like virus 4, **DANV:** Drosophila American nodavirus, **CYV:** Culex Y virus, **CpDNV:** Culex pipiens densovirus

Supplementary Tables: References

1. Suzuki Y, Frangeul L, Dickson LB, Blanc H, Verdier Y, Vinh J, Lambrechts L, Saleh MC. 2017. Uncovering the Repertoire of Endogenous Flaviviral Elements in *Aedes* Mosquito Genomes. J Virol 91:e00571-17.
2. McBride CS, Baier F, Omondi AB, Spitzer SA, Lutomiah J, Sang R, Ignell R, Vosshall LB. 2014. Evolution of mosquito preference for humans linked to an odorant receptor. Nature 515:222-7.
3. Eng MW, Clemons A, Hill C, Engel R, Severson DW, Behura SK. 2018. Multifaceted functional implications of an endogenously expressed tRNA fragment in the vector mosquito *Aedes aegypti*. PLoS Negl Trop Dis 12:e0006186.
4. Baidaliuk A, Miot EF, Lequime S, Moltini-Conclois I, Delaigue F, Dabo S, Dickson LB, Aubry F, Merkling SH, Cao-Lormeau VM, Lambrechts L. 2019. Cell-Fusing Agent Virus Reduces Arbovirus Dissemination in *Aedes aegypti* Mosquitoes In Vivo. J Virol 93:JVI.00705-19.
5. Zakrzewski M, Rasic G, Darbro J, Krause L, Poo YS, Filipovic I, Parry R, Asgari S, Devine G, Suhrbier A. 2018. Mapping the virome in wild-caught *Aedes aegypti* from Cairns and Bangkok. Sci Rep 8:4690.
6. Koh C, Allen SL, Herbert RI, McGraw EA, Chenoweth SF. 2018. The Transcriptional Response of *Aedes aegypti* with Variable Extrinsic Incubation Periods for Dengue Virus. Genome Biol Evol 10:3141-3151.
7. Etebari K, Osei-Amo S, Blomberg SP, Asgari S. 2015. Dengue virus infection alters post-transcriptional modification of microRNAs in the mosquito vector *Aedes aegypti*. Sci Rep 5:15968.
8. Wimalasiri-Yapa B, Barrero RA, Stassen L, Hafner LM, McGraw EA, Pyke AT, Jansen CC, Suhrbier A, Yakob L, Hu W, Devine GJ, Frentiu FD. 2021. Temperature modulates immune gene expression in mosquitoes during arbovirus infection. Open Biol 11:200246.
9. Slonchak A, Hugo LE, Freney ME, Hall-Mendelin S, Amarilla AA, Torres FJ, Setoh YX, Peng NYG, Sng JDJ, Hall RA, van den Hurk AF, Devine GJ, Khromykh AA. 2020. Zika virus noncoding RNA suppresses apoptosis and is required for virus transmission by mosquitoes. Nat Commun 11:2205.
10. Lee M, Etebari K, Hall-Mendelin S, van den Hurk AF, Hobson-Peters J, Vatipally S, Schnettler E, Hall R, Asgari S. 2017. Understanding the role of microRNAs in the interaction of *Aedes aegypti* mosquitoes with an insect-specific flavivirus. J Gen Virol 98:1892-1903.
11. Batool K, Alam I, Wu S, Liu W, Zhao G, Chen M, Wang J, Xu J, Huang T, Pan X, Yu X, Guan X, Xu L, Zhang L. 2018. Transcriptomic Analysis of *Aedes aegypti* in Response to Mosquitocidal Bacillus thuringiensis LLP29 Toxin. Sci Rep 8:12650.
12. Faucon F, Gaude T, Dusfour I, Navratil V, Corbel V, Juntarajumnong W, Girod R, Poupardin R, Boyer F, Reynaud S, David JP. 2017. In the hunt for genomic markers of metabolic resistance to pyrethroids in the mosquito *Aedes aegypti:* An integrated next-generation sequencing approach. PLoS Negl Trop Dis 11:e0005526.
13. Tetreau G, Bayyareddy K, Jones CM, Stalinski R, Riaz MA, Paris M, David JP, Adang MJ, Despres L. 2012. Larval midgut modifications associated with Bti resistance in the yellow fever mosquito using proteomic and transcriptomic approaches. BMC Genomics 13:248.
14. David JP, Faucon F, Chandor-Proust A, Poupardin R, Riaz MA, Bonin A, Navratil V, Reynaud S. 2014. Comparative analysis of response to selection with three insecticides in the dengue mosquito *Aedes aegypti* using mRNA sequencing. BMC Genomics 15:174.
15. Stalinski R, Laporte F, Tetreau G, Despres L. 2016. Receptors are affected by selection with each *Bacillus thuringiensis israelensis* Cry toxin but not with the full Bti mixture in *Aedes aegypti*. Infect Genet Evol 44:218-227.
16. Lequime S, Richard V, Cao-Lormeau VM, Lambrechts L. 2017. Full-genome dengue virus sequencing in mosquito saliva shows lack of convergent positive selection during transmission by *Aedes aegypti*. Virus Evol 3:vex031.
17. Shrinet J, Srivastava P, Sunil S. 2017. Transcriptome analysis of Aedes aegypti in response to mono-infections and co-infections of dengue virus-2 and chikungunya virus. Biochem Biophys Res Commun 492:617-623.
18. Cattel J, Haberkorn C, Laporte F, Gaude T, Cumer T, Renaud J, Sutherland IW, Hertz JC, Bonneville J, Arnaud V, Fustec B, Boyer S, Marcombe S, David J. 2021. A genomic amplification affecting a carboxylesterase gene cluster confers organophosphate resistance in the mosquito *Aedes aegypti*: From genomic characterization to high-throughput field detection. Evol Appl DOI: 10.1111/eva.13177.
19. Sutton ER, Yu Y, Shimeld SM, White-Cooper H, Alphey AL. 2016. Identification of genes for engineering the male germline of *Aedes aegypti* and *Ceratitis capitata*. BMC Genomics 17:948.
20. Camargo C, Ahmed-Braimah YH, Amaro IA, Harrington LC, Wolfner MF, Avila FW. 2020. Mating and blood-feeding induce transcriptome changes in the spermathecae of the yellow fever mosquito *Aedes aegypti*. Sci Rep 10:14899.
21. Alfonso-Parra C, Ahmed-Braimah YH, Degner EC, Avila FW, Villarreal SM, Pleiss JA, Wolfner MF, Harrington LC. 2016. Mating-Induced Transcriptome Changes in the Reproductive Tract of Female *Aedes aegypti*. PLoS Negl Trop Dis 10:e0004451.
22. Degner EC, Ahmed-Braimah YH, Borziak K, Wolfner MF, Harrington LC, Dorus S. 2019. Proteins, Transcripts, and Genetic Architecture of Seminal Fluid and Sperm in the Mosquito *Aedes aegypti*. Mol Cell Proteomics 18:S6-S22.
23. Halbach R, Miesen P, Joosten J, Taskopru E, Rondeel I, Pennings B, Vogels CBF, Merkling SH, Koenraadt CJ, Lambrechts L, van Rij RP. 2020. A satellite repeat-derived piRNA controls embryonic development of *Aedes*. Nature 580:274-277.

24. Chandler JA, Thongsripong P, Green A, Kittayapong P, Wilcox BA, Schroth GP, Kapan DD, Bennett SN. 2014. Metagenomic shotgun sequencing of a Bunyavirus in wild-caught *Aedes aegypti* from Thailand informs the evolutionary and genomic history of the Phleboviruses. Virology 464-465:312-319.
25. Raquin V, Merkling SH, Gausson V, Moltini-Conclois I, Frangeul L, Varet H, Dillies MA, Saleh MC, Lambrechts L. 2017. Individual co-variation between viral RNA load and gene expression reveals novel host factors during early dengue virus infection of the *Aedes aegypti* midgut. PLoS Negl Trop Dis 11:e0006152.
26. Baidaliuk A, Lequime S, Moltini-Conclois I, Dabo S, Dickson LB, Prot M, Duong V, Dussart P, Boyer S, Shi C, Matthijnssens J, Guglielmini J, Gloria-Soria A, Simon-Loriere E, Lambrechts L. 2020. Novel genome sequences of cell-fusing agent virus allow comparison of virus phylogeny with the genetic structure of *Aedes aegypti* populations. Virus Evol 6:veaa018.
27. Choy MM, Sessions OM, Gubler DJ, Ooi EE. 2015. Production of Infectious Dengue Virus in *Aedes aegypti* Is Dependent on the Ubiquitin Proteasome Pathway. PLoS Negl Trop Dis 9:e0004227.
28. Chowdhury A, Modahl CM, Tan ST, Wong Wei Xiang B, Misse D, Vial T, Kini RM, Pompon JF. 2020. JNK pathway restricts DENV2, ZIKV and CHIKV infection by activating complement and apoptosis in mosquito salivary glands. PLoS Pathog 16:e1008754.
29. Aguiar ER, Olmo RP, Paro S, Ferreira FV, de Faria IJ, Todjro YM, Lobo FP, Kroon EG, Meignin C, Gatherer D, Imler JL, Marques JT. 2015. Sequence-independent characterization of viruses based on the pattern of viral small RNAs produced by the host. Nucleic Acids Res 43:6191-206.
30. Olmo RP, Ferreira AGA, Izidoro-Toledo TC, Aguiar E, de Faria IJS, de Souza KPR, Osorio KP, Kuhn L, Hammann P, de Andrade EG, Todjro YM, Rocha MN, Leite T, Amadou SCG, Armache JN, Paro S, de Oliveira CD, Carvalho FD, Moreira LA, Marois E, Imler JL, Marques JT. 2018. Control of dengue virus in the midgut of *Aedes aegypti* by ectopic expression of the dsRNA-binding protein Loqs2. Nat Microbiol 3:1385-1393.
31. Bona AC, Chitolina RF, Fermino ML, de Castro Poncio L, Weiss A, Lima JB, Paldi N, Bernardes ES, Henen J, Maori E. 2016. Larval application of sodium channel homologous dsRNA restores pyrethroid insecticide susceptibility in a resistant adult mosquito population. Parasit Vectors 9:397.
32. Shi C, Beller L, Deboutte W, Yinda KC, Delang L, Vega-Rua A, Failloux AB, Matthijnssens J. 2019. Stable distinct core eukaryotic viromes in different mosquito species from Guadeloupe, using single mosquito viral metagenomics. Microbiome 7:121.
33. Bonizzoni M, Dunn WA, Campbell CL, Olson KE, Marinotti O, James AA. 2012. Strain Variation in the Transcriptome of the Dengue Fever Vector, *Aedes aegypti*. G3 (Bethesda) 2:103-14.
34. Bonizzoni M, Dunn WA, Campbell CL, Olson KE, Marinotti O, James AA. 2012. Complex modulation of the *Aedes aegypti* transcriptome in response to dengue virus infection. PLoS One 7:e50512.
35. Bonizzoni M, Britton M, Marinotti O, Dunn WA, Fass J, James AA. 2013. Probing functional polymorphisms in the dengue vector, *Aedes aegypti*. BMC Genomics 14:739.
36. Ruckert C, Prasad AN, Garcia-Luna SM, Robison A, Grubaugh ND, Weger-Lucarelli J, Ebel GD. 2019. Small RNA responses of Culex mosquitoes and cell lines during acute and persistent virus infection. Insect Biochem Mol Biol 109:13-23.
37. Fauver JR, Akter S, Morales AIO, Black WCt, Rodriguez AD, Stenglein MD, Ebel GD, Weger-Lucarelli J. 2019. A reverse-transcription/RNase H based protocol for depletion of mosquito ribosomal RNA facilitates viral intrahost evolution analysis, transcriptomics and pathogen discovery. Virology 528:181-197.
38. Canton PE, Cancino-Rodezno A, Gill SS, Soberon M, Bravo A. 2015. Transcriptional cellular responses in midgut tissue of *Aedes aegypti* larvae following intoxication with Cry11Aa toxin from Bacillus thuringiensis. BMC Genomics 16:1042.
39. Cross ST, Maertens BL, Dunham TJ, Rodgers CP, Brehm AL, Miller MR, Williams AM, Foy BD, Stenglein MD. 2020. Partitiviruses Infecting *Drosophila melanogaster* and *Aedes aegypti* Exhibit Efficient Biparental Vertical Transmission. J Virol 94: e01070-20.
40. Ferreira PG, Tesla B, Horacio ECA, Nahum LA, Brindley MA, de Oliveira Mendes TA, Murdock CC. 2020. Temperature Dramatically Shapes Mosquito Gene Expression With Consequences for Mosquito-Zika Virus Interactions. Front Microbiol 11:901.
41. Frey KG, Biser T, Hamilton T, Santos CJ, Pimentel G, Mokashi VP, Bishop-Lilly KA. 2016. Bioinformatic Characterization of Mosquito Viromes within the Eastern United States and Puerto Rico: Discovery of Novel Viruses. Evol Bioinform Online 12:1-12.
42. Kang DS, Barron MS, Lovin DD, Cunningham JM, Eng MW, Chadee DD, Li J, Severson DW. 2018. A transcriptomic survey of the impact of environmental stress on response to dengue virus in the mosquito, *Aedes aegypti*. PLoS Negl Trop Dis 12:e0006568.
43. Saldana MA, Etebari K, Hart CE, Widen SG, Wood TG, Thangamani S, Asgari S, Hughes GL. 2017. Zika virus alters the microRNA expression profile and elicits an RNAi response in *Aedes aegypti* mosquitoes. PLoS Negl Trop Dis 11:e0005760.
44. Etebari K, Hegde S, Saldana MA, Widen SG, Wood TG, Asgari S, Hughes GL. 2017. Global Transcriptome Analysis of *Aedes aegypti* Mosquitoes in Response to Zika Virus Infection. mSphere 2: e00456-17.
45. Lewis SH, Quarles KA, Yang Y, Tanguy M, Frézal L, Smith SA, Sharma PP, Cordaux R, Gilbert C, Giraud I. 2017. Pan-arthropod analysis reveals somatic piRNAs as an ancestral defence against transposable elements. Nat Ecol Evol:1: 174-181.
46. Metsky HC, Matranga CB, Wohl S, Schaffner SF, Freije CA, Winnicki SM, West K, Qu J, Baniecki ML, Gladden-Young A, Lin AE, Tomkins-Tinch CH, Ye SH, Park DJ, Luo CY, Barnes KG, Shah RR, Chak B, Barbosa-Lima G, Delatorre E, Vieira YR, Paul LM, Tan AL, Barcellona CM, Porcelli MC, Vasquez C, Cannons AC, Cone MR,

Hogan KN, Kopp EW, Anzinger JJ, Garcia KF, Parham LA, Ramirez RMG, Montoya MCM, Rojas DP, Brown CM, Hennigan S, Sabina B, Scotland S, Gangavarapu K, Grubaugh ND, Oliveira G, Robles-Sikisaka R, Rambaut A, Gehrke L, Smole S, Halloran ME, Villar L, Mattar S, et al. 2017. Zika virus evolution and spread in the Americas. Nature 546:411-415.

47. Zhao L, Alto BW, Shin D, Yu F. 2018. The Effect of Permethrin Resistance on *Aedes aegypti* Transcriptome Following Ingestion of Zika Virus Infected Blood. Viruses 10: 470.
48. Arensburger P, Hice RH, Wright JA, Craig NL, Atkinson PW. 2011. The mosquito *Aedes aegypti* has a large genome size and high transposable element load but contains a low proportion of transposon-specific piRNAs. BMC Genomics 12:606.
49. Boyles SM, Mavian CN, Finol E, Ukhanova M, Stephenson CJ, Hamerlinck G, Kang S, Baumgartner C, Geesey M, Stinton I, Williams K, Mathias DK, Prosperi M, Mai V, Salemi M, Buckner EA, Lednicky JA, Rivers AR, Dinglasan RR. 2020. Under-the-Radar Dengue Virus Infections in Natural Populations of *Aedes aegypti* Mosquitoes. mSphere 5.
50. Campbell CL, Harrison T, Hess AM, Ebel GD. 2014. MicroRNA levels are modulated in *Aedes aegypti* after exposure to Dengue-2. Insect Mol Biol 23:132-9.
51. Hess AM, Prasad AN, Ptitsyn A, Ebel GD, Olson KE, Barbacioru C, Monighetti C, Campbell CL. 2011. Small RNA profiling of Dengue virus-mosquito interactions implicates the PIWI RNA pathway in anti-viral defense. BMC Microbiol 11:45.
52. Dong S, Behura SK, Franz AWE. 2017. The midgut transcriptome of *Aedes aegypti* fed with saline or protein meals containing chikungunya virus reveals genes potentially involved in viral midgut escape. BMC Genomics 18:382.
53. Buchman A, Gamez S, Li M, Antoshechkin I, Li HH, Wang HW, Chen CH, Klein MJ, Duchemin JB, Paradkar PN, Akbari OS. 2019. Engineered resistance to Zika virus in transgenic *Aedes aegypti* expressing a polycistronic cluster of synthetic small RNAs. Proc Natl Acad Sci U S A 116:3656-3661.
54. Goertz GP, van Bree JWM, Hiralal A, Fernhout BM, Steffens C, Boeren S, Visser TM, Vogels CBF, Abbo SR, Fros JJ, Koenraadt CJM, van Oers MM, Pijlman GP. 2019. Subgenomic flavivirus RNA binds the mosquito DEAD/H-box helicase ME31B and determines Zika virus transmission by *Aedes aegypti*. Proc Natl Acad Sci U S A 116:19136-19144.
55. Nouzova M, Etebari K, Noriega FG, Asgari S. 2018. A comparative analysis of corpora allata-corpora cardiaca microRNA repertoires revealed significant changes during mosquito metamorphosis. Insect Biochem Mol Biol 96:10-18.
56. Grubaugh ND, Weger-Lucarelli J, Murrieta RA, Fauver JR, Garcia-Luna SM, Prasad AN, Black WCt, Ebel GD. 2016. Genetic Drift during Systemic Arbovirus Infection of Mosquito Vectors Leads to Decreased Relative Fitness during Host Switching. Cell Host Microbe 19:481-92.
57. Anglero-Rodriguez YI, MacLeod HJ, Kang S, Carlson JS, Jupatanakul N, Dimopoulos G. 2017. *Aedes aegypti* Molecular Responses to Zika Virus: Modulation of Infection by the Toll and Jak/Stat Immune Pathways and Virus Host Factors. Front Microbiol 8:2050.
58. Price DP, Nagarajan V, Churbanov A, Houde P, Milligan B, Drake LL, Gustafson JE, Hansen IA. 2011. The fat body transcriptomes of the yellow fever mosquito *Aedes aegypti*, pre- and post- blood meal. PLoS One 6:e22573.
59. Li Y, Piermarini PM, Esquivel CJ, Price DP, Drumm HE, Schilkey FD, Hansen IA. 2017. RNA-Seq Comparison of Larval and Adult Malpighian Tubules of the Yellow Fever Mosquito *Aedes aegypti* Reveals Life Stage-Specific Changes in Renal Function. Front Physiol 8:283.
60. Xiao X, Yang L, Pang X, Zhang R, Zhu Y, Wang P, Gao G, Cheng G. 2017. A Mesh-Duox pathway regulates homeostasis in the insect gut. Nat Microbiol 2:17020.
61. Tallon AK, Hill SR, Ignell R. 2019. Sex and age modulate antennal chemosensory-related genes linked to the onset of host seeking in the yellow-fever mosquito, *Aedes aegypti*. Sci Rep 9:43.
62. Alonso DP, Campos M, Troca H, Kunii R, Tripet F, Ribolla PEM. 2019. Gene expression profile of *Aedes aegypti* females in courtship and mating. Sci Rep 9:15492.
63. Neira-Oviedo M, Tsyganov-Bodounov A, Lycett GJ, Kokoza V, Raikhel AS, Krzywinski J. 2011. The RNA-Seq approach to studying the expression of mosquito mitochondrial genes. Insect Mol Biol 20:141-52.
64. Zhang X, Aksoy E, Girke T, Raikhel AS, Karginov FV. 2017. Transcriptome-wide microRNA and target dynamics in the fat body during the gonadotrophic cycle of *Aedes aegypti*. Proc Natl Acad Sci U S A 114:E1895-E1903.
65. Vogel KJ, Valzania L, Coon KL, Brown MR, Strand MR. 2017. Transcriptome Sequencing Reveals Large-Scale Changes in Axenic *Aedes aegypti* Larvae. PLoS Negl Trop Dis 11:e0005273.
66. Price DP, Schilkey FD, Ulanov A, Hansen IA. 2015. Small mosquitoes, large implications: crowding and starvation affects gene expression and nutrient accumulation in *Aedes aegypti*. Parasit Vectors 8:252.
67. Valzania L, Coon KL, Vogel KJ, Brown MR, Strand MR. 2018. Hypoxia-induced transcription factor signaling is essential for larval growth of the mosquito *Aedes aegypti*. Proc Natl Acad Sci U S A 115:457-465.
68. Adelman ZN, Anderson MA, Wiley MR, Murreddu MG, Samuel GH, Morazzani EM, Myles KM. 2013. Cooler temperatures destabilize RNA interference and increase susceptibility of disease vector mosquitoes to viral infection. PLoS Negl Trop Dis 7:e2239.
69. Morazzani EM, Wiley MR, Murreddu MG, Adelman ZN, Myles KM. 2012. Production of virus-derived ping-pong-dependent piRNA-like small RNAs in the mosquito soma. PLoS Pathog 8:e1002470.
70. Adelman ZN, Anderson MA, Liu M, Zhang L, Myles KM. 2012. Sindbis virus induces the production of a novel class of endogenous siRNAs in *Aedes aegypti* mosquitoes. Insect Mol Biol 21:357-68.

71. Hu W, Criscione F, Liang S, Tu Z. 2015. MicroRNAs of two medically important mosquito species: *Aedes aegypti* and *Anopheles stephensi.* Insect Mol Biol 24:240-52.
72. Biedler JK, Hu W, Tae H, Tu Z. 2012. Identification of early zygotic genes in the yellow fever mosquito *Aedes aegypti* and discovery of a motif involved in early zygotic genome activation. PLoS One 7:e33933.
73. Samuel GH, Wiley MR, Badawi A, Adelman ZN, Myles KM. 2016. Yellow fever virus capsid protein is a potent suppressor of RNA silencing that binds double-stranded RNA. Proc Natl Acad Sci U S A 113:13863-13868.
74. Jiang X, Biedler JK, Qi Y, Hall AB, Tu Z. 2015. Complete Dosage Compensation in *Anopheles stephensi* and the Evolution of Sex-Biased Genes in Mosquitoes. Genome Biol Evol 7:1914-24.
75. Zhu Y, Zhang R, Zhang B, Zhao T, Wang P, Liang G, Cheng G. 2017. Blood meal acquisition enhances arbovirus replication in mosquitoes through activation of the GABAergic system. Nat Commun 8:1262.
76. Liu J, Liu Y, Nie K, Du S, Qiu J, Pang X, Wang P, Cheng G. 2016. Flavivirus NS1 protein in infected host sera enhances viral acquisition by mosquitoes. Nat Microbiol 1:16087.
77. Liu P, Fu X, Zhu J. 2018. Juvenile hormone-regulated alternative splicing of the taiman gene primes the ecdysteroid response in adult mosquitoes. Proc Natl Acad Sci U S A 115:E7738-E7747.
78. Prud'homme SM, Renault D, David JP, Reynaud S. 2018. Multiscale Approach to Deciphering the Molecular Mechanisms Involved in the Direct and Intergenerational Effect of Ibuprofen on Mosquito *Aedes aegypti*. Environ Sci Technol 52:7937-7950.
79. Ribeiro JM, Martin-Martin I, Arca B, Calvo E. 2016. A Deep Insight into the Sialome of Male and Female *Aedes aegypti* Mosquitoes. PLoS One 11:e0151400.
80. Bonizzoni M, Dunn WA, Campbell CL, Olson KE, Dimon MT, Marinotti O, James AA. 2011. RNA-seq analyses of blood-induced changes in gene expression in the mosquito vector species, *Aedes aegypti*. BMC Genomics 12:82.
81. Choi YJ, Aliota MT, Mayhew GF, Erickson SM, Christensen BM. 2014. Dual RNA-seq of parasite and host reveals gene expression dynamics during filarial worm-mosquito interactions. PLoS Negl Trop Dis 8:e2905.
82. Juneja P, Ariani CV, Ho YS, Akorli J, Palmer WJ, Pain A, Jiggins FM. 2015. Exome and transcriptome sequencing of *Aedes aegypti* identifies a locus that confers resistance to Brugia malayi and alters the immune response. PLoS Pathog 11:e1004765.
83. Matthews BJ, Dudchenko O, Kingan SB, Koren S, Antoshechkin I, Crawford JE, Glassford WJ, Herre M, Redmond SN, Rose NH, Weedall GD, Wu Y, Batra SS, Brito-Sierra CA, Buckingham SD, Campbell CL, Chan S, Cox E, Evans BR, Fansiri T, Filipovic I, Fontaine A, Gloria-Soria A, Hall R, Joardar VS, Jones AK, Kay RGG, Kodali VK, Lee J, Lycett GJ, Mitchell SN, Muehling J, Murphy MR, Omer AD, Partridge FA, Peluso P, Aiden AP, Ramasamy V, Rasic G, Roy S, Saavedra-Rodriguez K, Sharan S, Sharma A, Smith ML, Turner J, Weakley AM, Zhao Z, Akbari OS, Black WCt, Cao H, et al. 2018. Improved reference genome of *Aedes aegypti* informs arbovirus vector control. Nature 563:501-507.
84. Matthews BJ, McBride CS, DeGennaro M, Despo O, Vosshall LB. 2016. The neurotranscriptome of the *Aedes aegypti* mosquito. BMC Genomics 17:32.
85. Chung M, Teigen L, Libro S, Bromley RE, Kumar N, Sadzewicz L, Tallon LJ, Foster JM, Michalski ML, Dunning Hotopp JC. 2018. Multispecies Transcriptomics Data Set of *Brugia malayi*, Its *Wolbachia* Endosymbiont wBm, and *Aedes aegypti* across the B. malayi Life Cycle. Microbiol Resour Announc 7: e01306-18.
86. Gibbons JG, Janson EM, Hittinger CT, Johnston M, Abbot P, Rokas A. 2009. Benchmarking next-generation transcriptome sequencing for functional and evolutionary genomics. Mol Biol Evol 26:2731-44.
87. Edgerton EB, McCrea AR, Berry CT, Kwok JY, Thompson LK, Watson B, Fuller EM, Nolan TJ, Lok JB, Povelones M. 2020. Activation of mosquito immunity blocks the development of transmission-stage filarial nematodes. Proc Natl Acad Sci U S A 117:3711-3717.
88. Hall AB, Basu S, Jiang X, Qi Y, Timoshevskiy VA, Biedler JK, Sharakhova MV, Elahi R, Anderson MA, Chen XG, Sharakhov IV, Adelman ZN, Tu Z. 2015. SEX DETERMINATION. A male-determining factor in the mosquito *Aedes aegypti.* Science 348:1268-70.
89. Dietrich I, Jansen S, Fall G, Lorenzen S, Rudolf M, Huber K, Heitmann A, Schicht S, Ndiaye EH, Watson M, Castelli I, Brennan B, Elliott RM, Diallo M, Sall AA, Failloux AB, Schnettler E, Kohl A, Becker SC. 2017. RNA Interference Restricts Rift Valley Fever Virus in Multiple Insect Systems. mSphere 2:e00090.
90. Tsujimoto H, Hanley KA, Sundararajan A, Devitt NP, Schilkey FD, Hansen IA. 2018. Correction: Dengue virus serotype 2 infection alters midgut and carcass gene expression in the Asian tiger mosquito, *Aedes albopictus*. PLoS One 13:e0192128.
91. Esquivel CJ, Cassone BJ, Piermarini PM. 2016. A de novo transcriptome of the Malpighian tubules in non-blood-fed and blood-fed Asian tiger mosquitoes *Aedes albopictus*: insights into diuresis, detoxification, and blood meal processing. PeerJ 4:e1784.
92. Esquivel CJ, Cassone BJ, Piermarini PM. 2014. Transcriptomic evidence for a dramatic functional transition of the malpighian tubules after a blood meal in the Asian tiger mosquito *Aedes albopictus*. PLoS Negl Trop Dis 8:e2929.
93. Huang X, Poelchau MF, Armbruster PA. 2015. Global transcriptional dynamics of diapause induction in non-blood-fed and blood-fed *Aedes albopictus*. PLoS Negl Trop Dis 9:e0003724.
94. Poelchau MF, Reynolds JA, Denlinger DL, Elsik CG, Armbruster PA. 2011. A de novo transcriptome of the Asian tiger mosquito, *Aedes albopictus*, to identify candidate transcripts for diapause preparation. BMC Genomics 12:619.
95. Poelchau MF, Reynolds JA, Elsik CG, Denlinger DL, Armbruster PA. 2013. Deep sequencing reveals complex mechanisms of diapause preparation in the invasive mosquito, *Aedes albopictus*. Proc Biol Sci 280:20130143.

96. Poelchau MF, Reynolds JA, Denlinger DL, Elsik CG, Armbruster PA. 2013. Transcriptome sequencing as a platform to elucidate molecular components of the diapause response in the Asian tiger mosquito, *Aedes albopictus*. Physiol Entomol 38:173-181.
97. Batz ZA, Goff AC, Armbruster PA. 2017. MicroRNAs are differentially abundant during *Aedes albopictus* diapause maintenance but not diapause induction. Insect Mol Biol 26:721-733.
98. Boyle JH, Rastas PMA, Huang X, Garner AG, Vythilingam I, Armbruster PA. 2021. A Linkage-Based Genome Assembly for the Mosquito *Aedes albopictus* and Identification of Chromosomal Regions Affecting Diapause. Insects 12: 167.
99. Boes KE, Ribeiro JM, Wong A, Harrington LC, Wolfner MF, Sirot LK. 2014. Identification and characterization of seminal fluid proteins in the Asian tiger mosquito, *Aedes albopictus*. PLoS Negl Trop Dis 8:e2946.
100. Gamez S, Antoshechkin I, Mendez-Sanchez SC, Akbari OS. 2020. The Developmental Transcriptome of *Aedes albopictus*, a Major Worldwide Human Disease Vector. G3 (Bethesda) 10:1051-1062.
101. Ma Q, Srivastav SP, Gamez S, Dayama G, Feitosa-Suntheimer F, Patterson EI, Johnson RM, Matson EM, Gold AS, Brackney DE, Connor JH, Colpitts TM, Hughes GL, Rasgon JL, Nolan T, Akbari OS, Lau NC. 2021. A mosquito small RNA genomics resource reveals dynamic evolution and host responses to viruses and transposons. Genome Res 31:512-528.
102. Vedururu RK, Neave MJ, Tachedjian M, Klein MJ, Gorry PR, Duchemin JB, Paradkar PN. 2019. RNASeq Analysis of *Aedes albopictus* Mosquito Midguts after Chikungunya Virus Infection. Viruses 11: 513.
103. Vedururu RK, Neave MJ, Sundaramoorthy V, Green D, Harper JA, Gorry PR, Duchemin JB, Paradkar PN. 2019. Whole Transcriptome Analysis of *Aedes albopictus* Mosquito Head and Thorax Post-Chikungunya Virus Infection. Pathogens 8: 132.
104. Palatini U, Masri RA, Cosme LV, Koren S, Thibaud-Nissen F, Biedler JK, Krsticevic F, Johnston JS, Halbach R, Crawford JE, Antoshechkin I, Failloux AB, Pischedda E, Marconcini M, Ghurye J, Rhie A, Sharma A, Karagodin DA, Jenrette J, Gamez S, Miesen P, Masterson P, Caccone A, Sharakhova MV, Tu Z, Papathanos PA, Van Rij RP, Akbari OS, Powell J, Phillippy AM, Bonizzoni M. 2020. Improved reference genome of the arboviral vector *Aedes albopictus*. Genome Biol 21:215.
105. Marconcini M, Pischedda E, Houé V, Palatini U, Lozada-Chávez N, Sogliani D, Failloux AB, Bonizzoni M. 2021. Profile of Small RNAs, vDNA Forms and Viral Integrations in Late Chikungunya Virus Infection of *Aedes albopictus* Mosquitoes. Viruses 13:553.
106. Jin B, Zhao Y, Liu P, Sun Y, Li X, Zhang X, Chen XG, Gu J. 2021. The direct regulation of Aalbdsx on AalVgR is indispensable for ovarian development in *Aedes albopictus*. Pest Manag Sci 77:1654-1667.
107. Gu J, Hu W, Wu J, Zheng P, Chen M, James AA, Chen X, Tu Z. 2013. miRNA genes of an invasive vector mosquito, *Aedes albopictus*. PLoS One 8:e67638.
108. Liu YX, Li FX, Liu ZZ, Jia ZR, Zhou YH, Zhang H, Yan H, Zhou XQ, Chen XG. 2016. Integrated analysis of miRNAs and transcriptomes in *Aedes albopictus* midgut reveals the differential expression profiles of immune-related genes during dengue virus serotype-2 infection. Insect Sci 23:377-85.
109. Wang Y, Jin B, Liu P, Li J, Chen X, Gu J. 2018. piRNA Profiling of Dengue Virus Type 2-Infected Asian Tiger Mosquito and Midgut Tissues. Viruses 10: 213.
110. Liu Y, Zhou Y, Wu J, Zheng P, Li Y, Zheng X, Puthiyakunnon S, Tu Z, Chen XG. 2015. The expression profile of Aedes albopictus miRNAs is altered by dengue virus serotype-2 infection. Cell Biosci 5:16.
111. Chen XG, Jiang X, Gu J, Xu M, Wu Y, Deng Y, Zhang C, Bonizzoni M, Dermauw W, Vontas J, Armbruster P, Huang X, Yang Y, Zhang H, He W, Peng H, Liu Y, Wu K, Chen J, Lirakis M, Topalis P, Van Leeuwen T, Hall AB, Jiang X, Thorpe C, Mueller RL, Sun C, Waterhouse RM, Yan G, Tu ZJ, Fang X, James AA. 2015. Genome sequence of the Asian Tiger mosquito, Aedes albopictus, reveals insights into its biology, genetics, and evolution. Proc Natl Acad Sci U S A 112:E5907-15.
112. Xu Y, Dong Y, Xu Y, Lai Z, Jin B, Hao Y, Gao Y, Sun Y, Chen XG, Gu J. 2019. Differentiation of Long Non-Coding RNA and mRNA Expression Profiles in Male and Female *Aedes albopictus*. Front Genet 10:975.
113. Xu J, Su X, Bonizzoni M, Zhong D, Li Y, Zhou G, Nguyen H, Tong S, Yan G, Chen XG. 2018. Comparative transcriptome analysis and RNA interference reveal CYP6A8 and SNPs related to pyrethroid resistance in *Aedes albopictus*. PLoS Negl Trop Dis 12:e0006828.
114. Xiao P, Han J, Zhang Y, Li C, Guo X, Wen S, Tian M, Li Y, Wang M, Liu H, Ren J, Zhou H, Lu H, Jin N. 2018. Metagenomic Analysis of Flaviviridae in Mosquito Viromes Isolated From Yunnan Province in China Reveals Genes From Dengue and Zika Viruses. Front Cell Infect Microbiol 8:359.
115. Xiao P, Li C, Zhang Y, Han J, Guo X, Xie L, Tian M, Li Y, Wang M, Liu H, Ren J, Zhou H, Lu H, Jin N. 2018. Metagenomic Sequencing From Mosquitoes in China Reveals a Variety of Insect and Human Viruses. Front Cell Infect Microbiol 8:364.
116. Shi M, Lin XD, Tian JH, Chen LJ, Chen X, Li CX, Qin XC, Li J, Cao JP, Eden JS, Buchmann J, Wang W, Xu J, Holmes EC, Zhang YZ. 2016. Redefining the invertebrate RNA virosphere. Nature 540:539-543.
117. Frangeul L, Blanc H, Saleh MC, Suzuki Y. 2020. Differential Small RNA Responses against Co-Infecting Insect-Specific Viruses in *Aedes albopictus* Mosquitoes. Viruses 12: 468.
118. Goic B, Stapleford KA, Frangeul L, Doucet AJ, Gausson V, Blanc H, Schemmel-Jofre N, Cristofari G, Lambrechts L, Vignuzzi M, Saleh MC. 2016. Virus-derived DNA drives mosquito vector tolerance to arboviral infection. Nat Commun 7:12410.
119. Bellone R, Lequime S, Jupille H, Goertz GP, Aubry F, Mousson L, Piorkowski G, Yen PS, Gabiane G, Vazeille M, Sakuntabhai A, Pijlman GP, de Lamballerie X, Lambrechts L, Failloux AB. 2020. Experimental adaptation of dengue virus 1 to *Aedes albopictus* mosquitoes by in vivo selection. Sci Rep 10:18404.

120. Grigoraki L, Lagnel J, Kioulos I, Kampouraki A, Morou E, Labbe P, Weill M, Vontas J. 2015. Transcriptome Profiling and Genetic Study Reveal Amplified Carboxylesterase Genes Implicated in Temephos Resistance, in the Asian Tiger Mosquito *Aedes albopictus*. PLoS Negl Trop Dis 9:e0003771.
121. Lombardo F, Salvemini M, Fiorillo C, Nolan T, Zwiebel LJ, Ribeiro JM, Arca B. 2017. Deciphering the olfactory repertoire of the tiger mosquito *Aedes albopictus*. BMC Genomics 18:770.
122. Dittmer J, Alafndi A, Gabrieli P. 2019. Fat body-specific vitellogenin expression regulates host-seeking behaviour in the mosquito *Aedes albopictus*. PLoS Biol 17:e3000238.
123. Kubacki J, Flacio E, Qi W, Guidi V, Tonolla M, Fraefel C. 2020. Viral Metagenomic Analysis of *Aedes albopictus* Mosquitos from Southern Switzerland. Viruses 12: 929.
124. Sharma A, Reyes J, Borgmeyer D, Ayala-Chavez C, Snow K, Arshad F, Nuss A, Gulia-Nuss M. 2020. The sugar substitute erythritol shortens the lifespan of *Aedes aegypti* potentially by N-linked protein glycosylation. Sci Rep 10:6195.
125. Shi M, Lin XD, Tian JH, Chen LJ, Chen X, Li CX, Qin XC, Li J, Cao JP, Eden JS, Buchmann J, Wang W, Xu J, Holmes EC, Zhang YZ. 2016. Redefining the invertebrate RNA virosphere. Nature 540:539–543.
126. Manni M, Zdobnov EM. 2020. A Novel Anphevirus in *Aedes albopictus* Mosquitoes Is Distributed Worldwide and Interacts with the Host RNA Interference Pathway. Viruses 12: 1264.
127. Shi C, Zhao L, Atoni E, Zeng W, Hu X, Matthijnssens J, Yuan Z, Xia H. 2020. Stability of the Virome in Lab- and Field-Collected Aedes albopictus Mosquitoes across Different Developmental Stages and Possible Core Viruses in the Publicly Available Virome Data of *Aedes* Mosquitoes. mSystems 5: e00640-20.
128. Bishop C, Parry R, Asgari S. 2020. Effect of *Wolbachia* wAlbB on a positive-sense RNA negev-like virus: a novel virus persistently infecting *Aedes albopictus* mosquitoes and cells. J Gen Virol 101:216-225.
129. Maringer K, Yousuf A, Heesom KJ, Fan J, Lee D, Fernandez-Sesma A, Bessant C, Matthews DA, Davidson AD. 2017. Proteomics informed by transcriptomics for characterising active transposable elements and genome annotation in *Aedes aegypti*. BMC Genomics 18:101.
130. Mayoral JG, Etebari K, Hussain M, Khromykh AA, Asgari S. 2014. *Wolbachia* infection modifies the profile, shuttling and structure of microRNAs in a mosquito cell line. PLoS One 9:e96107.
131. Mayoral JG, Hussain M, Joubert DA, Iturbe-Ormaetxe I, O'Neill SL, Asgari S. 2014. *Wolbachia* small noncoding RNAs and their role in cross-kingdom communications. Proc Natl Acad Sci U S A 111:18721-6.
132. Xu YP, Qiu Y, Zhang B, Chen G, Chen Q, Wang M, Mo F, Xu J, Wu J, Zhang RR, Cheng ML, Zhang NN, Lyu B, Zhu WL, Wu MH, Ye Q, Zhang D, Man JH, Li XF, Cui J, Xu Z, Hu B, Zhou X, Qin CF. 2019. Zika virus infection induces RNAi-mediated antiviral immunity in human neural progenitors and brain organoids. Cell Res 29:265-273.
133. Tassetto M, Kunitomi M, Whitfield ZJ, Dolan PT, Sanchez-Vargas I, Garcia-Knight M, Ribiero I, Chen T, Olson KE, Andino R. 2019. Control of RNA viruses in mosquito cells through the acquisition of vDNA and endogenous viral elements. eLife 8:e05378.
134. Weger-Lucarelli J, Ruckert C, Grubaugh ND, Misencik MJ, Armstrong PM, Stenglein MD, Ebel GD, Brackney DE. 2018. Adventitious viruses persistently infect three commonly used mosquito cell lines. Virology 521:175-180.
135. Varjak M, Dietrich I, Sreenu VB, Till BE, Merits A, Kohl A, Schnettler E. 2018. Spindle-E Acts Antivirally Against Alphaviruses in Mosquito Cells. Viruses 10: 88.
136. Varjak M, Donald CL, Mottram TJ, Sreenu VB, Merits A, Maringer K, Schnettler E, Kohl A. 2017. Characterization of the Zika virus induced small RNA response in *Aedes aegypti* cells. PLoS Negl Trop Dis 11:e0006010.
137. Goertz GP, Miesen P, Overheul GJ, van Rij RP, van Oers MM, Pijlman GP. 2019. Mosquito Small RNA Responses to West Nile and Insect-Specific Virus Infections in *Aedes* and *Culex* Mosquito Cells. Viruses 11: 271.
138. Dietrich I, Shi X, McFarlane M, Watson M, Blomstrom AL, Skelton JK, Kohl A, Elliott RM, Schnettler E. 2017. The Antiviral RNAi Response in Vector and Non-vector Cells against Orthobunyaviruses. PLoS Negl Trop Dis 11:e0005272.
139. Skalsky RL, Vanlandingham DL, Scholle F, Higgs S, Cullen BR. 2010. Identification of microRNAs expressed in two mosquito vectors, *Aedes albopictus* and *Culex quinquefasciatus*. BMC Genomics 11:119.
140. Hermanns K, Marklewitz M, Zirkel F, Overheul GJ, Page RA, Loaiza JR, Drosten C, van Rij RP, Junglen S. 2020. Agua Salud alphavirus defines a novel lineage of insect-specific alphaviruses discovered in the New World. J Gen Virol 101:96-104.
141. Du J, Gao S, Tian Z, Guo Y, Kang D, Xing S, Zhang G, Liu G, Luo J, Chang H, Yin H. 2019. Transcriptome analysis of responses to bluetongue virus infection in *Aedes albopictus* cells. BMC Microbiol 19:121.
142. Li M, Xing D, Su D, Wang D, Gao H, Lan C, Gu Z, Zhao T, Li C. 2021. Transcriptome Analysis of Responses to Dengue Virus 2 Infection in *Aedes albopictus* (Skuse) C6/36 Cells. Viruses 13: 343.
143. Teramoto T, Huang X, Armbruster PA, Padmanabhan R. 2019. Infection of *Aedes albopictus* Mosquito C6/36 Cells with the wMelpop Strain of *Wolbachia* Modulates Dengue Virus-Induced Host Cellular Transcripts and Induces Critical Sequence Alterations in the Dengue Viral Genome. J Virol 93.
144. Azlan A, Obeidat SM, Theva Das K, Yunus MA, Azzam G. 2021. Genome-wide identification of *Aedes albopictus* long noncoding RNAs and their association with dengue and Zika virus infection. PLoS Negl Trop Dis 15:e0008351.

145. Peccoud J, Lequime S, Moltini-Conclois I, Giraud I, Lambrechts L, Gilbert C. 2018. A Survey of Virus Recombination Uncovers Canonical Features of Artificial Chimeras Generated During Deep Sequencing Library Preparation. G3 (Bethesda) 8:1129-1138.
146. Miller JR, Koren S, Dilley KA, Puri V, Brown DM, Harkins DM, Thibaud-Nissen F, Rosen B, Chen XG, Tu Z, Sharakhov IV, Sharakhova MV, Sebra R, Stockwell TB, Bergman NH, Sutton GG, Phillippy AM, Piermarini PM, Shabman RS. 2018. Analysis of the *Aedes albopictus* C6/36 genome provides insight into cell line utility for viral propagation. Gigascience 7:1-13.
147. Avila-Bonilla RG, Yocupicio-Monroy M, Marchat LA, De Nova-Ocampo MA, Del Angel RM, Salas-Benito JS. 2017. Analysis of the miRNA profile in C6/36 cells persistently infected with dengue virus type 2. Virus Res 232:139-151.
148. Bishop C, Asgari S. 2020. Altered gene expression profile of Wolbachia pipientis wAlbB strain following transinfection from its native host *Aedes albopictus* to *Aedes aegypti* cells. Mol Microbiol doi:10.1111/mmi.14668.
149. Darby AC, Gill AC, Armstrong SD, Hartley CS, Xia D, Wastling JM, Makepeace BL. 2014. Integrated transcriptomic and proteomic analysis of the global response of *Wolbachia* to doxycycline-induced stress. ISME J 8:925-37.
150. Woolfit M, Algama M, Keith JM, McGraw EA, Popovici J. 2015. Discovery of putative small non-coding RNAs from the obligate intracellular bacterium *Wolbachia pipientis*. PLoS One 10:e0118595.
